# Design and Synthesis of Planar Chiral Bisphosphine Ligands Based on Diphenyl [2.2]-Paracyclophane

**DOI:** 10.3390/molecules31030494

**Published:** 2026-01-31

**Authors:** Shaoying Huang, Yingjie Huang, Jiaping Jin, Haorui Gu, Xufeng Lin

**Affiliations:** Department of Chemistry, Zhejiang University, Hangzhou 310058, China; 12137005@zju.edu.cn (S.H.); 22337015@zju.edu.cn (Y.H.); jinjiaping@cirs-group.com (J.J.)

**Keywords:** planar, bisphosphine ligands, chiral, paracyclophane

## Abstract

Planar chiral bisphosphine ligands based on diphenyl [2.2]paracyclophane (PhPhanePHOS) were successfully synthesized in a practical manner in four steps from commercially available 4,12-bisbromo-[2.2]paracyclophane as a new family of bisphosphine ligands. The novel PhPhanePHOS ligands provide high catalytic activity in Pd-catalyzed asymmetric allylic alkylation reactions in preliminary experiments.

## 1. Introduction

**Figure 1:**
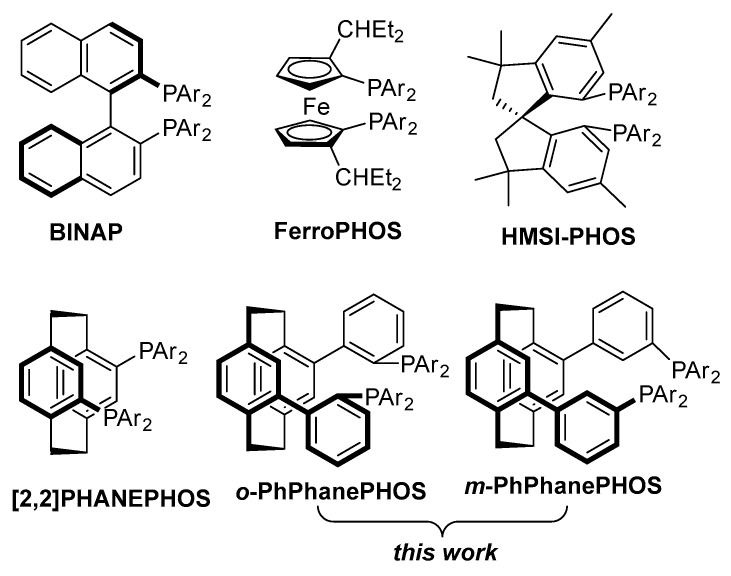
Chiral bisphosphine ligands.

The development of chiral ligands plays a pivotal role in the development of highly efficient transition metal-catalyzed asymmetric reactions [1,2,3,4,5,6], in which chiral bisphosphine ligands are particularly important due to their widespread application in many areas [7,8,9]. The design of innovative frameworks was the basis for the development of chiral ligands. In the last few decades, various types of backbones for chiral bisphosphines (Figure 1), such as binaphthyl [10,11,12,13,14,15], biphenyl [16,17,18], heteroaryl [19,20], cyclophane [21,22], ferrocenyl [23,24,25,26,27], and spiro [28,29,30] backbones, have been developed and have proven to be effective and useful in the field of metal-catalyzed asymmetric reactions. Despite the impressive and extensive advances in this field, the development of chiral bisphosphines bearing novel scaffolds that support novel catalysis strategies [31,32,33] and are characterized by excellent efficiency and ready accessibility is still a major challenge, and their availability would be valuable in meeting the need for excellent enantioselective transformations with potential industrial applications.

In recent years, planar chirality has been increasingly used in the preparation of catalysts [34,35,36,37,38,39,40,41,42,43]. Some research groups, such as Pye and Rossen, Knoll and Zippel, Falk and Fröhlich, Schwartz and Holmes, Nguyen and Herkommer, and Grasa and Zanotti-Gerosa, have successfully used planar chiral catalysts in a variety of organometallic enantioselective reactions, including hydrosilylation [44], hydrogenations [45,46,47], hydroxymethyl-ations [48,49], hydroaminations [50], and coupling [51] reactions. These new chiral catalysts, derived from [2.2]paracyclophane, exhibit special properties. Recently, our group has also developed a new type of planar chiral phosphoric acid (PCPA) based on [2.2]paracyclophane [52], which has been successfully used in asymmetric Aza–Friedel–Crafts reactions as a strong Brønsted acid organocatalyst.

Inspired by these pioneering studies, herein we report the design and synthesis of planar chiral bisphosphine ligands based on diphenyl [2.2]paracyclophane (PhPhanePHOS) and some preliminary catalytic experiments. Our strategy involved the strategic design of ortho- and meta-substituted ligand architectures to precisely control the chiral pocket geometry. We hypothesize that the introduction of a phenyl substitution into the planar framework of PhPhanePHOS freely alters the dihedral angle of the conformationally planar chiral bisphosphine. Chiral bisphosphines with phenyl substitutions in different positions can be readily diversified, exhibiting their different natures, which may significantly influence the enantioselectivity of catalytic reactions in some cases.

## 2. Results and Discussion

**Scheme 1:**
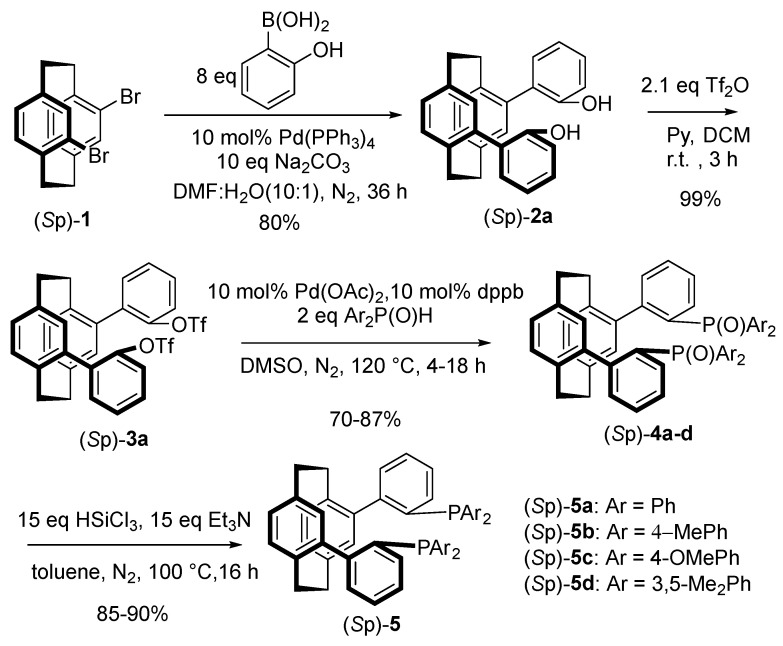
Synthesis of *o*-PhPhanePHOS **5a**–**d**.

**Scheme 2:**
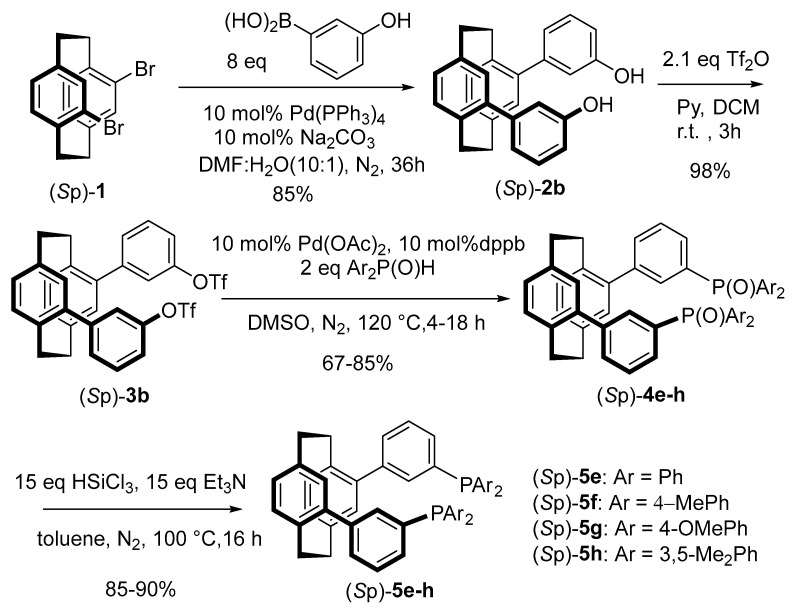
Synthesis of *m*-PhPhanePHOS **5e**–**h**.

Four planar chiral bisphosphines, **5a**–**d** (*o*-PhPhanePHOS), were synthesized via a four-step protocol outlined in Scheme 1. The synthetic route is initiated via a Suzuki–Miyaura cross-coupling reaction between commercially available (*S_p_*)-**1** ((*S_p_*)-4,12-dibromo[2.2]paracyclophane) and 2-hydroxyphenylboronic acid. The target compound (*S_p_*)-**2a** was obtained in an 80% yield catalyzed by 10 mol% Pd(PPh_3_)_4_. Compound (*S_p_*)-**2a** was treated with trifluoromethanesulfonic anhydride (Tf_2_O) in the presence of pyridine to afford the triflate derivative (*S_p_*)-**3a** in a 99% yield. Subsequently, (*S_p_*)-**3a** was subjected to a coupling reaction with Ar_2_P(O)H in the presence of a 10 mol% Pd(OAc)_2_/dppb catalytic system, affording the corresponding phosphine oxides, (*S_p_*)-**4a**–**d**, in yields ranging from 70% to 87%. Finally, *o*-PhPhanePHOS (*S_p_*)-**5a**–**d** was obtained in an 85–90% yield by reduction of phosphine oxide with HSiCl_3_. Thus, *o*-PhPhanePHOS was prepared in four steps with an overall yield of about 47–63%. Similarly, four other planar chiral bisphosphines, **5e**–**h** (*m*-PhPhanePHOS), were also prepared with good overall yields according to the above route, as shown in Scheme 2. We hypothesize that these ligands, *o*-PhPhanePHOS and m-PhPhanePHOS, may exhibit an intriguing effect on metal-catalyzed asymmetric reactions.

**Scheme 3:**
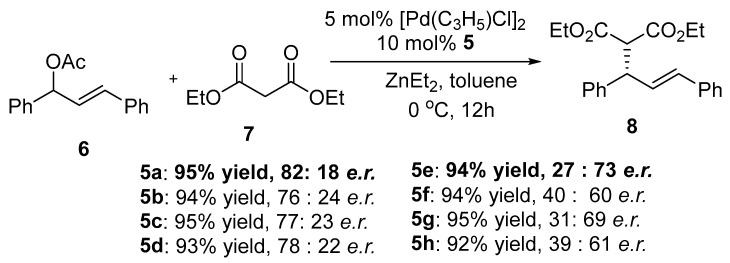
Asymmetric allylic alkylation reactions.

Furthermore, the efficiency of PhPhanePHOS in catalytic asymmetric allylic alkylation was evaluated [36,53,54,55,56,57,58,59,60]. As shown in Scheme 3, 1,3-diphenylpropen-1-yl acetate was treated with diethyl malonate using 5 mol% [Pd(C_3_H_5_)Cl]_2_ and 10 mol% of *o*-PhPhanePHOS **5a** in toluene, and the desired chiral product, **8**, was obtained in a 95% yield with an 82:18 *e.r*. The ligands *o*-PhPhanePHOS **5b**–**d** all gave similar results. On the other hand, the product, **8**, with **5e**–**h** as the chiral ligand was obtained in an excellent yield with a slightly low *e.r*., and the position of the phosphine group in PhPhanePHOS can greatly affect the enantioselectivity of the product, which had a completely opposite configuration. The initial application of these ligands, while not yet matching the excellent results obtained by predecessors in asymmetric allylic alkylation [59,60,61], revealed a remarkable inversion effect that demonstrates their potential in the field of metal catalysis.

**Figure 2:**
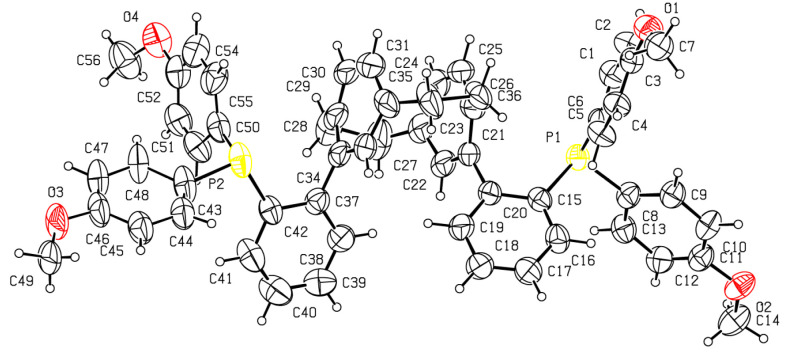
X-ray crystal structures of **5c** (CCDC 2526231).

**Figure 3:**
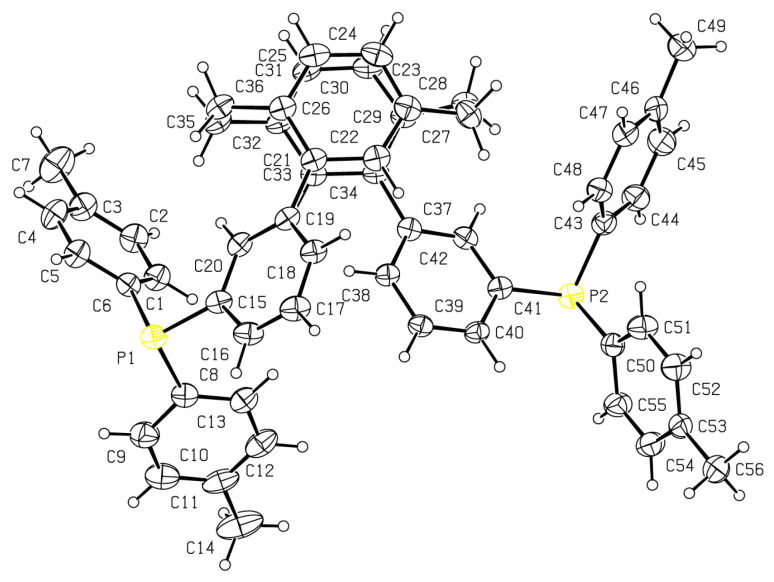
X-ray crystal structures of **5f** (CCDC 2526232).

**Figure 4:**
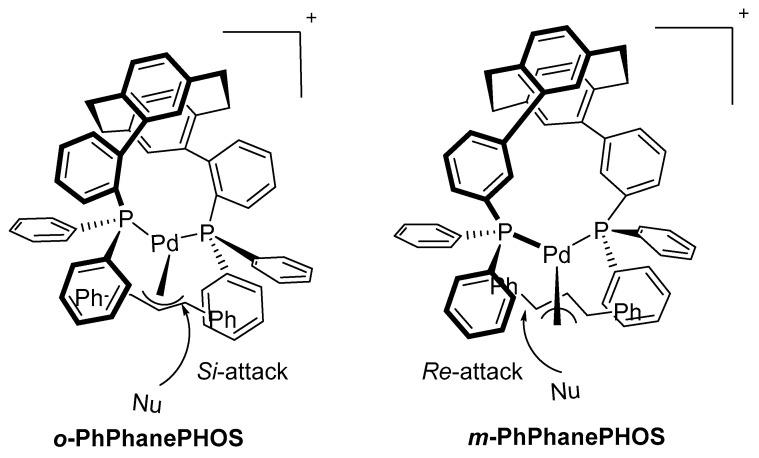
Catalytic model for Pd-PhPhanePHOS complex.

We failed to obtain an X-ray crystal structure analysis of the complex for [Pd(C_3_H_5_)Cl]_2_ and PhPhanePHOS. However, the X-ray crystal structures of ligands **5c** (CCDC 2526231) and **5f** (CCDC 2526232) were obtained, as shown in Figure 2 and Figure 3. Here, we proposed a catalytic model for these ligands via an intermediate Pd-complex featuring an η^3^-coordinated π-allylic system [54] (Figure 4). In this conformation, the phenyl groups adopt a propeller-like arrangement to minimize steric repulsion, creating a chiral environment in which the back-right and front-left quadrants are effectively shielded. Meanwhile, the substituent position of the bisdiphenylphosphine influences both the steric contour and the electronic environment of the complex. Upon binding with the π-allylic intermediate, the π-allyl palladium complexes form different transition states. Subsequently, the π-allyl palladium complexes are attacked by the nucleophile via opposite paths, yielding the product with an inverted configuration.

## 3. Materials and Methods

### 3.1. General Experimental Procedures

All reagents and solvents were purchased from Bidepharm (Shanghai, China) or Energy Chemical (Shanghai, China) and used without any further purification. ^1^H NMR, ^31^P and ^13^C NMR spectra were recorded with a Bruker AVANCE III 400M superconducting NMR spectrometer at 25 °C, with n CDCl_3_ or DMSO-*d_6_* as the lock solvent. The working solution concentration for NMR was 20 mg/mL. The chemical shifts (δ) were quoted in parts per million (ppm) downfield relative to the internal standard TMS (0.0 ppm) and referenced to solvent peaks in the NMR solvent (CDCl_3_ = δ 7.26 ppm; δ 77.16 ppm; DMSO-*d_6_* = δ 2.50 ppm; δ 39.5 ppm). Spin multiplicity was reported using the following abbreviations: s = singlet, d = doublet, t = triplet, dd = doublet of doublet, td = triplet of doublet, m = multiplet. Infrared spectra were recorded on an ATR-FTIR spectrometer. HRMS spectra were determined using a Waters GCT Premier (Waters Corporation, Milford, MA, USA). Optical rotations were determined using a Perkin Elmer Model 341 polarimeter (PerkinElmer, Inc., Waltham, MA, USA) at 20 °C. Enantiomeric excesses (ees) were determined by chiral high-performance liquid chromatography. HPLC analysis was performed using Chiralcel columns.

### 3.2. General Procedure for the Synthesis and Application of Planar Chiral Bisphosphine Ligands

#### 3.2.1. Synthesis of Compound (*S_p_*)-**2**

A 100 mL three-necked round-bottom flask with a condenser was charged with (*S*)-4,12-dibromo[2.2]paracyclophane ((*S_p_*)-**1**) (3 mmol, 1.0 equiv), 2-hydroxyphenylboronic acid or 3-hydroxyphenylboronic acid (24 mmol, 8 equiv), Pd(PPh_3_)_4_ (0.3 mmol, 0.1 equiv), and Na_2_CO_3_ (30 mmol, 10.0 equiv) in the mixed solvent DMSO:H_2_O (10:1) (30 mL) under a nitrogen atmosphere, and then the mixture was stirred at 100 °C for 36 h under oil bath. After cooling to room temperature, the resulting mixture was diluted with EtOAc (100 mL) and 1 M HCl (100 mL). The resulting mixture was then extracted three times with EtOAc (3 × 50 mL). The organic layer was washed with brine and dried over anhydrous Na_2_SO_4_. The organic layer was filtered, and the filtrate was concentrated under reduced pressure. The residue was purified by flash column chromatography (petroleum ether/ethyl acetate = 10/1) to give the product, **2**, as a white solid.

*(S_p_)-2,2′-(1,4(1,4)-dibenzenacyclohexaphane-1^2^,4^3^-diyl)diphenol [(S_p_)-***2a***]*. White solid (940 mg, 80% yield); M.p. 218–219 °C; [α${]}_{D}^{20}$ = 88.2° (c 1.00, DCM); ^1^H NMR (400 MHz, CDCl_3_) δ 7.63 (dd, *J* = 7.6, 1.4 Hz, 2H), 7.33–7.25 (m, 2H), 7.03 (m, 2H), 6.95 (d, *J* = 8.1 Hz, 2H), 6.83 (d, *J* = 7.4 Hz, 2H), 6.72 (m, 4H), 5.21 (s, 2H), 3.22 (m, 2H), 3.17–3.07 (m, 2H), 2.90 (m, 2H), 2.65 (m, 2H) ppm; ^13^C NMR (101 MHz, CDCl_3_) δ 152.64, 141.20, 138.11, 136.14, 133.41, 132.96, 131.36, 129.27, 128.40, 126.95, 120.45, 115.49, 34.53, 34.32 ppm; IR (film): γ = 3457, 3015, 2922, 2885, 2851, 1903, 1583, 1489, 1473, 1446, 1404, 1344, 1270, 1226, 1196, 1094, 1038, 1023, 952, 916, 811, 760, 736, 649, 510 cm^−1^; HRMS (GC-TOF): calculated for C_28_H_23_O_2_^−^([M-H]^−^): *m*/*z* 391.1698, found: 391.1703.

*(S_p_)-3,3′-(1,4(1,4)-dibenzenacyclohexaphane-1^2^,4^3^-diyl)diphenol [(S_p_)-***2b***]*. White solid (998 mg, 85% yield); M.p. 214–215 °C; [α${]}_{D}^{20}$ = 526.8° (c 1.00, DCM); ^1^H NMR (400 MHz, DMSO) δ 9.42 (s, 2H), 7.17 (t, *J* = 7.8 Hz, 2H), 6.81–6.59 (m, 10H), 6.53 (d, *J* = 1.3 Hz, 2H), 3.41 (d, *J* = 12.4 Hz, 1H), 3.37 (s, 1H), 3.12–3.01 (m, 2H), 2.93 (m, 2H), 2.67 (m, 2H) ppm; ^13^C NMR (101 MHz, DMSO) δ 157.11, 142.02, 139.82, 139.06, 136.39, 135.42, 132.05, 129.43, 129.37, 119.19, 115.77, 113.67, 33.85, 33.73 ppm; IR (film): γ = 3306, 3206, 2923, 2852, 1655, 1579, 1468, 1405, 1340, 1300, 1210, 1160, 1047, 1024, 1004, 882, 789, 736, 722, 703 cm^−1^; HRMS (GC-TOF): calculated for C_28_H_23_O_2_^−^([M-H]^−^): *m*/*z* 391.1698, found: 391.1705.

#### 3.2.2. Synthesis of Compound (*S_p_*)-**3**

To a solution of (*S_p_*)-**2** (1.3 g, 2.6 mmol) and pyridine (0.4 mL, 5.5 mmol) in CH_2_Cl_2_ (25 mL), Tf_2_O (0.4 mL, 5.5 mmol) was added at 0 °C. The resulting mixture was stirred under a nitrogen atmosphere for 3 h. After completion of the reaction, 1 M HCl (10 mL) was added to the mixture. The resulting mixture was then extracted three times with CH_2_Cl_2_ (3 × 25 mL). The organic phase was separated and washed with saturated NaHCO_3_ and brine, then dried over anhydrous Na_2_SO_4_. *T*he organic layer was filtered, and the filtrate was concentrated under reduced pressure. The residue was purified by flash column chromatography (petroleum ether/ethyl acetate = 30/1) to afford (*S_p_*)-**3**.

*(S_p_)-1,4(1,4)-dibenzenacyclohexaphane-1^2^,4^3^-diylbis(2,1-phenylene) bis(trifluoromethanesulfonate) [(S_p_)-***3a***]*. 1.69 g, 99% yield; white solid; M.p. 129–130 °C; [α${]}_{D}^{20}$ = 18.0° (c 1.00, DCM); ^1^H NMR (400 MHz, CDCl_3_) δ 7.78–7.67 (m, 2H), 7.52–7.41 (m, 4H), 7.39–7.28 (m, 2H), 6.80 (d, *J* = 7.7 Hz, 2H), 6.75 (dd, *J* = 7.8, 1.5 Hz, 2H), 6.62 (d, *J* = 1.0 Hz, 2H), 3.21–3.12 (m, 2H), 3.12–3.03 (m, 2H), 3.02–2.91 (m, 2H), 2.70–2.55 (m, 2H) ppm; ^13^C NMR (101 MHz, CDCl_3_) δ 147.24, 139.17, 138.57, 135.40, 134.93, 134.09, 131.40, 130.57, 130.15, 128.99, 128.22, 122.21, 119.78, 116.59, 34.51, 33.94 ppm; IR (film): γ = 3010, 2933, 2858, 1589, 1475, 1422, 1245, 1209, 1145, 1090, 1045, 949, 916, 885, 856, 824, 779, 767, 741, 650, 627, 595, 572, 503 cm^−1^; HRMS (GC-TOF): calculated for C_30_H_22_F_6_NaO_6_S_2_^+^ ([M+Na]^+^): *m*/*z* 679.0660, found: 679.0656.

*(S_p_)-1,4(1,4)-dibenzenacyclohexaphane-1^2^,4^3^-diylbis(3,1-phenylene) bis(trifluoromethanesulfonate) [(S_p_)-***3b***]*. 1.67 g, 98% yield; colorless oil; [α${]}_{D}^{20}$ = −266.7° (c 1.00, DCM); ^1^H NMR (400 MHz, CDCl_3_) δ 7.57 (d, *J* = 7.6 Hz, 1H), 7.51–7.42 (m, 2H), 7.30 (dd, *J* = 17.9, 7.6 Hz, 3H), 7.23 (d, *J* = 0.9 Hz, 2H), 6.76 (d, *J* = 7.7 Hz, 2H), 6.70 (dd, *J* = 7.7, 1.5 Hz, 2H), 6.60 (d, *J* = 1.6 Hz, 2H), 3.62–3.36 (m, 2H), 3.22–3.08 (m, 2H), 3.00 (m, 2H), 2.75 (m, 2H) ppm; ^13^C NMR (101 MHz, CDCl_3_) δ 149.64, 143.47, 139.90, 138.28, 137.12, 135.99, 133.23, 130.30, 129.84, 129.01, 121.73, 119.41, 34.44, 34.00 ppm; IR (film): γ = 3007, 2934, 2860, 1890, 1609, 1573, 1476, 1424, 1245, 1212, 1140, 1044, 943, 893, 863, 847, 796, 759, 734, 696, 606, 573, 514 cm^−1^; HRMS (GC-TOF): calculated for C_30_H_22_F_6_NaO_6_S_2_^+^ ([M+Na]^+^): *m*/*z* 679.0660, found: 679.0653.

#### 3.2.3. Synthesis of Compound (*S_p_*)-**4**

A 25 mL three-necked round-bottom flask with a condenser was charged with (*S_p_*)-**3** (1 mmol, 1.0 equiv), Ar_2_P(O)H (4 mmol, 2.0 equiv), palladium acetate (0.1 mmol, 0.1 equiv), 1,4-bis(diphenylphosphino)butane (dppb, 0.1 mmol, 0.1 equiv), and N,N-diisopropylethylamine (0.7 mL, 4.0 equiv), and the resulting mixture was stirred in degassed DMSO (10 mL) in an oil bath at 120 °C under a dry nitrogen atmosphere. The progress of the reaction was monitored by TLC until complete conversion. After cooling to room temperature, the reaction solution was diluted with water and the aqueous phase was extracted three times with EtOAc (3 × 20 mL). The organic layer was washed sequentially with 5% aqueous HCl, saturated NaHCO_3_ and brine; dried over anhydrous Na_2_SO_4_; and then the mixture was filtered, and the filtrate was concentrated under reduced pressure. The resulting residue was purified by flash chromatography (ethyl acetate/petroleum ether = 2/1) to give the target product.

*(S_p_)-(1,4(1,4)-dibenzenacyclohexaphane-1^2^,4^3^-diylbis(2,1-phenylene))bis(diphenylphosphine oxide) [(S_p_)-***4a***]*. 661 mg, 87% yield; white solid; M.p. 164–165 °C; [α${]}_{D}^{20}$ = −354.0° (c 1.00, DCM); ^1^H NMR (400 MHz, CDCl_3_) δ 7.67 (dd, *J* = 7.2, 4.2 Hz, 2H), 7.54 (t, *J* = 6.4 Hz, 2H), 7.41–7.31 (m, 12H), 7.29 (d, *J* = 3.6 Hz, 3H), 7.26–7.20 (m, 5H), 7.20–7.12 (m, 4H), 6.37 (s, 2H), 6.26 (d, *J* = 7.6 Hz, 2H), 6.12 (d, *J* = 7.7 Hz, 2H), 2.90–2.65 (m, 4H), 2.55–2.38 (m, 4H) ppm; ^13^C NMR (101 MHz, CDCl_3_) δ 146.61 (d, *J* = 8.5 Hz), 138.56, 137.51, 134.70 (d, *J* = 4.1 Hz), 134.25 (t, *J* = 6.2 Hz), 133.54 (d, *J* = 7.4 Hz), 133.23, 132.46, 132.20, 131.45, 131.36, 131.27, 130.81, 129.55 (d, *J* = 9.6 Hz), 127.89 (t, *J* = 12.3 Hz), 126.29 (d, *J* = 12.6 Hz), 34.23, 34.00 ppm; ^31^P NMR (162 MHz, CDCl_3_) δ 26.78 (s) ppm; IR (film): γ = 3386, 3054, 2956, 2926, 2853, 1717, 1588, 1562, 1483, 1459, 1437, 1403, 1378, 1263, 1194, 1115, 1030, 896, 863, 733, 720, 706, 542 cm^−1^; HRMS (GC-TOF): calculated for C_52_H_42_NaO_2_P_2_^+^([M+Na]^+^): *m*/*z* 783.2558, found: 783.2553.

*(S_p_)-(1,4(1,4)-dibenzenacyclohexaphane-1^2^,4^3^-diylbis(2,1-phenylene))bis(di-p-tolylphosphine oxide) [(S_p_)-***4b***]*. 694 mg, 85% yield; white solid; M.p. 134–135 °C; [α${]}_{D}^{20}$ = −393.3° (c 1.00, DCM); ^1^H NMR (400 MHz, CDCl_3_) δ 7.65 (d, *J* = 3.0 Hz, 2H), 7.51 (t, *J* = 7.1 Hz, 2H), 7.37–7.28 (m, 3H), 7.25–7.16 (m, 9H), 7.03 (d, *J* = 7.4 Hz, 4H), 6.97 (d, *J* = 7.6 Hz, 4H), 6.35 (s, 2H), 6.28 (d, *J* = 7.5 Hz, 2H), 6.12 (d, *J* = 7.2 Hz, 2H), 2.82 (t, *J* = 11.2 Hz, 2H), 2.71 (t, *J* = 12.0 Hz, 2H), 2.50–2.39 (m, 4H), 2.31 (s, 6H), 2.25 (s, 6H) ppm; ^13^C NMR (101 MHz, CDCl_3_) δ 146.55 (d, *J* = 8.4 Hz), 140.90, 138.44, 137.41, 134.80, 134.17 (d, *J* = 11.5 Hz), 133.78, 133.47, 131.28 (dd, *J* = 20.8, 11.2 Hz), 130.62, 129.55, 128.60 (t, *J* = 11.9 Hz), 126.15 (d, *J* = 12.7 Hz), 34.22, 34.05, 21.47, 21.39 ppm; ^31^P NMR (162 MHz, CDCl_3_) δ 27.00 (s) ppm; IR (film): γ = 3366, 3021, 2955, 2925, 2854, 1916, 1716, 1602, 1563, 1500, 1459, 1434, 1400, 1378, 1264, 1215, 1184, 1114, 1031, 1021, 895, 863, 808, 769, 733, 660, 620, 538, 517 cm^−1^; HRMS (GC-TOF): calculated for C_56_H_50_NaO_2_P_2_^+^([M+Na]^+^): *m*/*z* 839.3184, found: 839.3185.

*(S_p_)-(1,4(1,4)-dibenzenacyclohexaphane-1^2^,4^3^-diylbis(2,1-phenylene))bis(bis(4-methoxyphenyl)phosphine oxide) [(S_p_)-***4c***]*. 704 mg, 80% yield; white solid; M.p. 158–159 °C; [α${]}_{D}^{20}$ = −325.2° (c 1.00, DCM); ^1^H NMR (400 MHz, CDCl_3_) δ 7.65 (s, 2H), 7.52 (t, *J* = 6.7 Hz, 2H), 7.39–7.27 (m, 6H), 7.23 (d, *J* = 9.5 Hz, 6H), 6.75 (d, *J* = 7.7 Hz, 4H), 6.70 (d, *J* = 7.7 Hz, 4H), 6.36 (s, 2H), 6.30 (d, *J* = 7.2 Hz, 2H), 6.18 (d, *J* = 7.4 Hz, 2H), 3.78 (s, 6H), 3.73 (s, 6H), 2.83 (t, *J* = 11.1 Hz, 2H), 2.71 (t, *J* = 11.6 Hz, 2H), 2.55–2.38 (m, 4H) ppm; ^13^C NMR (101 MHz, CDCl_3_) δ 161.60 (d, *J* = 11.0 Hz), 146.43 (d, *J* = 8.4 Hz), 138.44, 137.47, 134.86, 134.17, 133.84, 133.56, 133.07 (d, *J* = 10.5 Hz), 132.81, 131.23 (d, *J* = 20.6 Hz), 129.54 (d, *J* = 9.5 Hz), 126.23 (d, *J* = 12.2 Hz), 125.14 (d, *J* = 11.0 Hz), 124.04 (d, *J* = 10.7 Hz), 113.55 (dd, *J* = 13.0, 7.1 Hz), 55.28 (d, *J* = 3.4 Hz), 34.25, 34.04 ppm; ^31^P NMR (162 MHz, CDCl_3_) δ 27.11 (s) ppm; IR (film): γ = 3378, 3004, 2928, 2840, 1907, 1716, 1597, 1570, 1502, 1461, 1405, 1293, 1253,1178, 1116, 1027, 863, 830, 801, 769, 734, 683, 664, 622, 549 cm^−1^; HRMS (GC-TOF): calculated for C_56_H_50_NaO_6_P_2_^+^([M+Na]^+^): *m*/*z* 903.2980, found: 903.2981.

*(S_p_)-(1,4(1,4)-dibenzenacyclohexaphane-1^2^,4^3^-diylbis(2,1-phenylene))bis(bis(3,5-dimethylphenyl)phosphine oxide) [(S_p_)-***4d***]*. 610 mg, 70% yield; yellow solid; M.p. 124–125 °C; [α${]}_{D}^{20}$ = −305.8° (c 1.00, DCM); ^1^H NMR (400 MHz, CDCl_3_) δ 7.64 (dd, *J* = 7.4, 4.2 Hz, 2H), 7.52 (t, *J* = 7.4 Hz, 2H), 7.39–7.28 (m, 6H), 7.00 (s, 2H), 6.97 (s, 2H), 6.94 (s, 4H), 6.87 (s, 2H), 6.39 (s, 2H), 6.27 (d, *J* = 7.6 Hz, 2H), 6.07 (d, *J* = 7.6 Hz, 2H), 2.90–2.80 (m, 2H), 2.79–2.68 (m, 2H), 2.54–2.41 (m, 4H), 2.17 (d, *J* = 11.6 Hz, 24H) ppm; ^13^C NMR (101 MHz, CDCl_3_) δ 138.28, 137.48, 137.32 (d, *J* = 6.3 Hz), 137.20 (d, *J* = 7.0 Hz), 133.99, 133.03, 132.45, 131.52, 131.03, 129.37, 129.32, 129.28, 129.05, 128.93 (d, *J* = 5.6 Hz), 128.81, 126.25, 34.32, 33.84, 21.15 ppm; ^31^P NMR (162 MHz, CDCl_3_) δ 26.82 (s) ppm; IR (film): γ = 3391, 2954, 2925, 2855, 1730, 1601, 1460, 1434, 1377, 1272, 1181, 1165, 1126, 1081, 1044, 870, 850, 769, 700, 577, 530,477 cm^−1^; HRMS (GC-TOF): calculated for C_60_H_58_NaO_2_P_2_^+^([M+Na]^+^): *m*/*z* 895.3810, found: 895.3812.

*(Sp)-(1,4(1,4)-dibenzenacyclohexaphane-1^2^,4^3^-diylbis(3,1-phenylene))bis(diphenylphosphine oxide) [(Sp)-***4e***]*. 646 mg, 85% yield; white solid; M.p. 95–96 °C; [α${]}_{D}^{20}$ = −627.7° (c 1.00, DCM); ^1^H NMR (400 MHz, CDCl_3_) δ 7.77–7.63 (m, 11H), 7.61–7.48 (m, 5H), 7.48–7.37 (m, 9H), 7.24 (d, *J* = 7.6 Hz, 2H), 6.98 (s, 1H), 6.64 (d, *J* = 7.7 Hz, 2H), 6.57 (dd, *J* = 7.7, 1.4 Hz, 2H), 6.34 (s, 2H), 3.30–3.10 (m, 2H), 3.02–2.91 (m, 2H), 2.88–2.71 (m, 2H), 2.42–2.33 (m, 2H); ^13^C NMR (101 MHz, CDCl_3_) δ 141.11 (d, *J* = 12.1 Hz), 139.64, 139.06, 137.01, 135.70, 133.24, 132.98, 132.68 (t, *J* = 5.4 Hz), 132.40 (d, *J* = 2.2 Hz), 132.18, 132.15, 132.10, 132.08, 132.05, 130.46 (d, *J* = 9.5 Hz), 129.80, 129.08 (d, *J* = 12.6 Hz), 128.64 (d, *J* = 12.1 Hz), 34.25, 34.06 ppm; ^31^P NMR (162 MHz, CDCl_3_) δ 29.74 (s) ppm; IR (film): γ = 3390, 3054, 2929, 2858, 1967, 1899, 1717, 1589, 1466, 1437, 1384, 1265, 1194, 1119, 1029, 998, 871, 800, 749, 724, 701, 596, 542 cm^−1^; HRMS (GC-TOF): calculated for C_52_H_42_NaO_2_P_2_^+^([M+Na]^+^): *m*/*z* 783.2558, found: 783.2549.

*(S_p_)-(1,4(1,4)-dibenzenacyclohexaphane-1^2^,4^3^-diylbis(3,1-phenylene))bis(di-p-tolylphosphine oxide) [(S_p_)-***4f***]*. 677 mg, 83% yield; white solid; M.p. 85–86 °C; [α${]}_{D}^{20}$ = −491.1° (c 1.00, DCM); ^1^H NMR (400 MHz, CDCl_3_) δ 7.73–7.52 (m, 12H), 7.39 (t, *J* = 6.2 Hz, 2H), 7.22 (d, *J* = 7.8 Hz, 10H), 6.64 (d, *J* = 7.7 Hz, 2H), 6.57 (dd, *J* = 7.7, 1.4 Hz, 2H), 6.33 (s, 2H), 3.28–3.18 (m, 2H), 3.04–2.92 (m, 2H), 2.88–2.76 (m, 2H), 2.40 (d, *J* = 7.0 Hz, 2H), 2.37 (s, 6H), 2.34 (s, 6H) ppm; ^13^C NMR (101 MHz, CDCl_3_) δ 142.52, 142.50, 142.48, 141.06, 140.94, 139.64, 139.18, 137.02, 135.64, 132.62, 132.17 (d, *J* = 2.9 Hz), 132.07 (d, *J* = 2.8 Hz), 130.45 (d, *J* = 9.5 Hz), 129.81, 129.04, 128.91, 34.18, 34.06, 21.58 ppm; ^31^P NMR (162 MHz, CDCl_3_) δ 29.85 (s) ppm; IR (film): γ = 3378, 3043, 2924, 2860, 1916, 1720, 1601, 1500, 1465, 1399, 1382, 1310, 1265, 1214, 1186, 1116, 1020, 871, 808, 763, 733, 712, 662, 621, 536, 518 cm^−1^; HRMS (GC-TOF): calculated for C_56_H_50_NaO_2_P_2_^+^([M+Na]^+^): *m*/*z* 839.3184, found: 839.3181.

*(S_p_)-(1,4(1,4)-dibenzenacyclohexaphane-1^2^,4^3^-diylbis(3,1-phenylene))bis(bis(4-methoxyphenyl)phosphine oxide) [(S_p_)-***4g***]*. 660 mg, 75% yield; white solid; M.p. 84–85 °C; [α${]}_{D}^{20}$ = −364.9° (c 1.00, DCM); ^1^H NMR (400 MHz, CDCl_3_) δ 7.73–7.57 (m, 12H), 7.46–7.39 (m, 2H), 7.22 (d, *J* = 7.6 Hz, 2H), 6.96–6.87 (m, 8H), 6.65 (d, *J* = 7.7 Hz, 2H), 6.58 (d, *J* = 7.7 Hz, 2H), 6.37 (s, 2H), 3.81 (s, 6H), 3.77 (s, 6H), 3.31–3.18 (m, 2H), 3.05–2.95 (m, 2H), 2.90–2.77 (m, 2H), 2.47–2.36 (m, 2H) ppm; ^13^C NMR (101 MHz, CDCl_3_) δ 162.49, 141.02 (d, *J* = 12.1 Hz), 139.64, 139.19, 137.02, 135.67, 133.92 (dd, *J* = 11.3, 3.0 Hz), 132.64, 132.14, 130.35 (d, *J* = 9.9 Hz), 129.83, 128.97 (d, *J* = 12.6 Hz), 124.44 (d, *J* = 19.3 Hz), 123.42, 114.17 (d, *J* = 13.2 Hz), 77.36, 77.05, 76.73, 55.36, 34.17 (d, *J* = 14.0 Hz) ppm; ^31^P NMR (162 MHz, CDCl_3_) δ 29.40 (s) ppm; IR (film): γ = 3367, 3005, 2927, 2841, 2553, 1908, 1717, 1597, 1569, 1504, 1463, 1406, 1382, 1294, 1256, 1179, 1119, 1026, 831, 801, 763, 733, 704, 667, 623, 547 cm^−1^; HRMS (GC-TOF): calculated for C_56_H_50_NaO_6_P_2_^+^([M+Na]^+^): *m*/*z* 903.2980, found: 903.2984.

*(S_p_)-(1,4(1,4)-dibenzenacyclohexaphane-1^2^,4^3^-diylbis(3,1-phenylene))bis(bis(3,5-dimethylphenyl)phosphine oxide) [(S_p_)-***4h***]*. 584 mg, 67% yield; yellow solid; M.p. 127–128 °C; [α${]}_{D}^{20}$ = −472.2° (c 1.00, DCM); ^1^H NMR (400 MHz, CDCl_3_) δ 7.80–7.71 (m, 2H), 7.66 (d, *J* = 12.4 Hz, 2H), 7.36–7.29 (m, 9H), 7.17–7.07 (m, 1H), 6.68–6.54 (m, 4H), 6.23 (s, 2H), 3.27 (t, *J* = 11.3 Hz, 2H), 3.00 (t, *J* = 11.4 Hz, 2H), 2.90–2.79 (m, 2H), 2.46–2.36 (m, 2H), 2.26 (s, 12H), 2.22 (s, 12H) ppm; ^13^C NMR (101 MHz, CDCl_3_) δ 140.93 (d, *J* = 12.1 Hz), 139.63, 139.29, 138.30 (d, *J* = 12.6 Hz), 137.03, 135.66, 133.82, 132.96, 132.75, 132.64, 132.14 (d, *J* = 42.1 Hz), 131.60, 130.75, 130.51 (d, *J* = 9.2 Hz), 129.87, 129.70 (d, *J* = 9.7 Hz), 129.12 (d, *J* = 12.5 Hz), 34.18, 33.98, 21.31, 21.26. ppm; ^31^P NMR (162 MHz, CDCl_3_) δ 30.18 (s) ppm; IR (film): γ = 3378, 2954, 2924, 2856, 1736, 1599, 1465, 1379, 1273, 1191, 1129, 1043, 873, 852, 800, 733, 693, 577, 524 cm^−1^; HRMS (GC-TOF): calculated for C_60_H_58_NaO_2_P_2_^+^([M+Na]^+^): *m*/*z* 895.3810, found: 895.3805.

#### 3.2.4. Synthesis of Compound (*S_p_*)-**5**

A 25 mL three-necked round-bottom flask with a condenser was charged with (*S_p_*)-**4** (0.5 mmol, 1.0 equiv), trichlorosilane (7.5 mmol, 15 equiv), and triethylamine (7.5 mmol, 15 equiv) in degassed toluene (5 mL), and the resulting mixture was stirred at 100 °C under a dry nitrogen atmosphere for 12h. After cooling to room temperature, the reaction solution was diluted with water and the aqueous phase was extracted three times with EtOAc (3 × 10 mL). The organic layer was washed with brine and dried over anhydrous Na_2_SO_4_. The organic layer was filtered, and the filtrate was concentrated under reduced pressure. The resulting residue was purified by flash chromatography (ethyl acetate/petroleum ether = 1/50) to give the target product, **5**.

*(S_p_)-1^2^,4^3^-bis(2-(diphenylphosphanyl)phenyl)-1,4(1,4)-dibenzenacyclohexaphane [(S_p_)-***5a***]*. 328 mg, 90% yield; white solid; M.p. 105–106 °C; [α${]}_{D}^{20}$ = 677.2° (c 1.00, DCM); ^1^H NMR (400 MHz, CDCl_3_) δ 7.82–7.69 (m, 2H), 7.46–7.38 (m, 2H), 7.33–7.22 (m, 8H), 7.21–7.06 (m, 10H), 7.02–6.87 (m, 6H), 6.56–6.48 (m, 4H), 6.45 (s, 2H), 3.01–2.81 (m, 2H), 2.79–2.64 (m, 2H), 2.59–2.35 (m, 4H) ppm; ^13^C NMR (101 MHz, CDCl_3_) δ 146.92 (d, *J* = 27.1 Hz), 138.58 (d, *J* = 3.4 Hz), 138.50 (d, *J* = 6.4 Hz), 137.64 (d, *J* = 12.3 Hz), 137.42 (d, *J* = 6.5 Hz), 137.15 (d, *J* = 12.9 Hz), 134.76, 134.41 (d, *J* = 20.8 Hz), 133.53 (d, *J* = 19.7 Hz), 133.33 (d, *J* = 35.8 Hz), 130.77 (d, *J* = 1.6 Hz), 128.43, 128.24 (d, *J* = 3.3 Hz), 128.15, 128.10 (d, *J* = 4.2 Hz), 127.96 (d, *J* = 4.8 Hz), 127.87, 127.03, 34.43, 34.31 (d, *J* = 4.0 Hz) ppm; ^31^P NMR (162 MHz, CDCl_3_) δ −13.24 (s) ppm; IR (film): γ = 3050, 2959, 2926, 2853, 1958, 1738, 1583, 1456, 1434, 1378, 1263, 1091, 1026, 862, 801, 743, 696, 498 cm^−1^; HRMS (GC-TOF): calculated for C_52_H_43_P_2_^+^([M+H]^+^): *m*/*z* 729.2840, found: 729.2839.

*(S_p_)-1^2^,4^3^-bis(2-(di-p-tolylphosphanyl)phenyl)-1,4(1,4)-dibenzenacyclohexaphane [(S_p_)-***5b***]*. 349 mg, 89% yield; white solid; M.p. 111–112 °C; [α${]}_{D}^{20}$ = −489.1° (c 1.00, DCM); ^1^H NMR (400 MHz, CDCl_3_) δ 7.75 (dd, *J* = 6.8, 4.2 Hz, 2H), 7.39 (td, *J* = 7.5, 1.3 Hz, 2H), 7.26 (s, 1H), 7.23 (dd, *J* = 7.5, 0.9 Hz, 1H), 7.09–7.01 (m, 8H), 7.00–6.95 (m, 2H), 6.93 (d, *J* = 7.5 Hz, 4H), 6.82 (t, *J* = 7.6 Hz, 4H), 6.56–6.49 (m, 4H), 6.46 (d, *J* = 1.2 Hz, 2H), 2.98–2.86 (m, 2H), 2.76–2.65 (m, 2H), 2.50–2.39 (m, 4H), 2.33 (s, 6H), 2.23 (s, 6H) ppm; ^13^C NMR (101 MHz, CDCl_3_) δ 146.88 (d, *J* = 27.1 Hz), 139.04 (d, *J* = 12.7 Hz), 138.79, 138.60, 137.95 (d, *J* = 53.0 Hz), 137.51 (d, *J* = 6.5 Hz), 134.72, 134.45 (d, *J* = 4.9 Hz), 134.29 (d, *J* = 5.1 Hz), 133.81 (d, *J* = 11.5 Hz), 133.56, 133.40 (d, *J* = 5.8 Hz), 133.09, 130.72, 128.97 (d, *J* = 7.3 Hz), 128.72 (d, *J* = 6.8 Hz), 128.06, 126.93, 34.43, 34.36 (d, *J* = 4.0 Hz), 21.29 (d, *J* = 13.6 Hz) ppm; ^31^P NMR (162 MHz, CDCl_3_) δ −15.33 (s) ppm; IR (film): γ = 3012, 2954, 2924, 2853, 1907, 1743, 1582, 1496, 1457, 1398, 1307, 1263, 1185, 1155, 1091, 1020, 862, 805, 767, 746, 627, 511 cm^−1^; HRMS (GC-TOF): calculated for C_56_H_51_P_2_^+^([M+H]^+^): *m*/*z* 785.3466, found: 785.3455.

*(S_p_)-1^2^,4^3^-bis(2-(bis(4-methoxyphenyl)phosphanyl)phenyl)-1,4(1,4)-dibenzenacyclohexaphane [(S_p_)-***5c***]*. 373 mg, 88% yield; white solid; M.p. 233–234 °C; [α${]}_{D}^{20}$ = −143.1° (c 1.00, DCM); ^1^H NMR (400 MHz, CDCl_3_) δ 7.78–7.68 (m, 2H), 7.38 (t, *J* = 7.3 Hz, 2H), 7.25–7.19 (m, 2H), 7.07 (t, *J* = 7.6 Hz, 4H), 6.97–6.90 (m, 2H), 6.86 (t, *J* = 7.5 Hz, 4H), 6.80 (d, *J* = 8.1 Hz, 4H), 6.66 (d, *J* = 8.1 Hz, 4H), 6.51 (q, *J* = 7.7 Hz, 4H), 6.43 (s, 2H), 3.79 (s, 6H), 3.70 (s, 6H), 2.98–2.84 (m, 2H), 2.79–2.64 (m, 2H), 2.53–2.38 (m, 4H) ppm; ^13^C NMR (101 MHz, CDCl_3_) δ 159.82 (d, *J* = 37.2 Hz), 139.62 (d, *J* = 12.9 Hz), 138.82, 138.54, 137.46 (d, *J* = 6.1 Hz), 135.88 (d, *J* = 22.2 Hz), 135.00 (d, *J* = 21.1 Hz), 134.73, 133.11, 130.74, 128.82 (d, *J* = 9.2 Hz), 128.35 (d, *J* = 9.9 Hz), 128.13, 127.46 (d, *J* = 105.7 Hz), 113.89 (d, *J* = 7.9 Hz), 113.67 (d, *J* = 7.5 Hz), 55.17 (d, *J* = 8.9 Hz), 34.51, 34.44 ppm; ^31^P NMR (162 MHz, CDCl_3_) δ −16.23 (s) ppm; IR (film): γ = 3048, 3002, 2956, 2927, 2835, 2043, 1893, 1593, 1567, 1497, 1456, 1441, 1402, 1284, 1246, 1177, 1094, 1030, 862, 825, 797, 768, 737, 532 cm^−1^; HRMS (GC-TOF): calculated for C_56_H_51_O_4_P_2_^+^([M+H]^+^): *m*/*z* 849.3263, found: 849.3249.

*(S_p_)-1^2^,4^3^-bis(2-(bis(3,5-dimethylphenyl)phosphanyl)phenyl)-1,4(1,4)-dibenzenacyclohexaphane [(S_p_)-***5d***]*. 357 mg, 85% yield; white solid; M.p. 95–96 °C; [α${]}_{D}^{20}$ = −388.2° (c 1.00, DCM); ^1^H NMR (400 MHz, CDCl_3_) δ 7.71 (dd, *J* = 7.0, 4.2 Hz, 2H), 7.39 (t, *J* = 7.1 Hz, 2H), 7.30–7.21 (m, 2H), 6.99 (dd, *J* = 7.2, 3.1 Hz, 2H), 6.90 (s, 2H), 6.76 (d, *J* = 8.8 Hz, 6H), 6.58 (d, *J* = 7.7 Hz, 4H), 6.53 (d, *J* = 7.5 Hz, 2H), 6.47 (d, *J* = 9.9 Hz, 4H), 2.92 (m, 2H), 2.78–2.63 (m, 2H), 2.58–2.38 (m, 4H), 2.22 (s, 12H), 2.12 (s, 12H) ppm; ^13^C NMR (101 MHz, CDCl_3_) δ 145.80 (d, *J* = 26.8 Hz), 138.03 (d, *J* = 13.4 Hz), 137.62, 137.45 (d, *J* = 2.3 Hz), 136.35 (d, *J* = 3.5 Hz), 136.30, 136.22, 136.01 (d, *J* = 7.1 Hz), 135.74 (d, *J* = 11.8 Hz), 133.62, 132.17 (d, *J* = 48.7 Hz), 131.14 (d, *J* = 21.0 Hz), 130.33 (d, *J* = 20.0 Hz), 129.72, 128.88 (d, *J* = 37.0 Hz), 126.92 (d, *J* = 4.8 Hz), 125.83, 33.49, 33.26, 20.22 (d, *J* = 8.3 Hz) ppm; ^31^P NMR (162 MHz, CDCl_3_) δ −12.80 (m) ppm; IR (film): γ = 3022, 2923, 2855, 2729, 1930, 1780, 1738, 1598, 1581, 1456, 1434, 1376, 1264, 1124, 1100, 1038, 946, 894, 847, 802, 767, 737, 694, 654, 553, 523, 492 cm^−1^; HRMS (GC-TOF): calculated for C_60_H_59_P_2_^+^([M+H]^+^): *m*/*z* 841.4092, found: 841.4092.

*(S_p_)-1^2^,4^3^-bis(3-(diphenylphosphanyl)phenyl)-1,4(1,4)-dibenzenacyclohexaphane [(S_p_)-***5e***]*. 328 mg, 90% yield; white solid; M.p. 91–92 °C; [α${]}_{D}^{20}$ = −107.5° (c 1.00, DCM) ppm; ^1^H NMR (400 MHz, CDCl_3_) δ 7.49–7.21 (m, 26H), 6.98 (d, *J* = 6.8 Hz, 2H), 6.58 (d, *J* = 7.7 Hz, 2H), 6.52 (dd, *J* = 7.7, 1.2 Hz, 2H), 6.29 (d, *J* = 1.3 Hz, 2H), 3.28–3.15 (m, 2H), 3.01–2.85 (m, 2H), 2.81–2.68 (m, 2H), 2.38–2.21 (m, 2H) ppm; ^13^C NMR (101 MHz, CDCl_3_) δ 141.16 (d, *J* = 6.3 Hz), 139.82, 139.61, 137.38 (d, *J* = 11.3 Hz), 137.17 (d, *J* = 11.4 Hz), 136.94, 135.40, 134.11, 133.95 (d, *J* = 2.7 Hz), 133.80 (d, *J* = 6.3 Hz), 133.64, 132.20, 132.01 (d, *J* = 21.1 Hz), 129.90 (d, *J* = 22.5 Hz), 128.84, 128.73, 128.68, 128.62 (d, *J* = 1.7 Hz), 128.56, 34.22, 34.08 ppm; ^31^P NMR (162 MHz, CDCl_3_) δ −5.12 (s) ppm; IR (film): γ = 3051, 2927, 2856, 1954, 1888, 1815, 1731, 1661, 1584, 1477, 1464, 1434, 1405, 1381, 1307, 1264, 1200, 1156, 1091, 1027, 998, 949, 912, 893, 872, 850, 797, 742, 696, 657, 593, 508 cm^−1^; HRMS (GC-TOF): calculated for C_52_H_43_P_2_^+^([M+H]^+^): *m*/*z* 729.2840, found: 729.2832.

*(S_p_)-1^2^,4^3^-bis(3-(di-p-tolylphosphanyl)phenyl)-1,4(1,4)-dibenzenacyclohexaphane [(S_p_)-***5f***]*. 353 mg, 90% yield; white solid; M.p. 75–76 °C; [α${]}_{D}^{20}$ = 163.6° (c 1.00, DCM); ^1^H NMR (400 MHz, CDCl_3_) δ 7.33 (d, *J* = 7.5 Hz, 2H), 7.30–7.19 (m, 12H), 7.11 (dd, *J* = 13.9, 7.6 Hz, 8H), 6.95 (d, *J* = 6.9 Hz, 2H), 6.59 (d, *J* = 7.7 Hz, 2H), 6.52 (d, *J* = 7.6 Hz, 2H), 6.31 (s, 2H), 3.31–3.15 (m, 2H), 3.00–2.87 (m, 2H), 2.83–2.70 (m, 2H), 2.30 (d, *J* = 15.2 Hz, 14H) ppm; ^13^C NMR (101 MHz, CDCl_3_) δ 141.17 (d, *J* = 6.1 Hz), 140.03, 139.74, 138.83 (d, *J* = 12.3 Hz), 137.95 (d, *J* = 11.4 Hz), 137.07, 135.48, 134.09, 133.97, 133.89, 133.78, 132.25, 131.89 (d, *J* = 20.9 Hz), 130.13, 129.63, 129.59, 129.53 (d, *J* = 2.8 Hz), 129.48, 128.80 (d, *J* = 7.6 Hz), 34.23, 21.43 (d, *J* = 3.0 Hz) ppm; ^31^P NMR (162 MHz, CDCl_3_) δ −6.80 (m) ppm; IR (film): γ = 3013, 2923, 2860, 1908, 1800, 1748, 1647, 1585, 1496, 1463, 1395, 1380, 1307, 1264, 1186, 1092, 1040, 1020, 948, 893, 872, 806, 767, 733, 710, 657, 641, 627, 612, 512 cm^−1^; HRMS (GC-TOF): calculated for C_56_H_51_P_2_^+^([M+H]^+^): *m*/*z* 785.3466, found: 785.3456.

*(S_p_)-1^2^,4^3^-bis(3-(bis(4-methoxyphenyl)phosphanyl)phenyl)-1,4(1,4)-dibenzenacyclohexaphane [(S_p_)-***5g***]*. 369 mg, 87% yield; white solid; M.p. 87–88 °C; [α${]}_{D}^{20}$ = 188.4° (c 1.00, DCM); ^1^H NMR (400 MHz, CDCl_3_) δ 7.35–7.19 (m, 14H), 6.96 (d, *J* = 6.9 Hz, 2H), 6.85 (t, *J* = 8.6 Hz, 8H), 6.59 (d, *J* = 7.7 Hz, 2H), 6.53 (d, *J* = 7.4 Hz, 2H), 6.32 (s, 2H), 3.76 (s, 6H), 3.73 (s, 6H), 3.30–3.17 (m, 2H), 3.00–2.87 (m, 2H), 2.84–2.71 (m, 2H), 2.41–2.27 (m, 2H); ^13^C NMR (101 MHz, CDCl_3_) δ 160.27 (d, *J* = 8.3 Hz), 141.07, 139.93, 139.59, 138.61 (d, *J* = 11.0 Hz), 136.93, 135.50, 135.39 (d, *J* = 3.2 Hz), 135.24 (d, *J* = 9.1 Hz), 133.45 (d, *J* = 18.2 Hz), 132.16, 131.32 (d, *J* = 19.6 Hz), 129.67 (d, *J* = 68.2 Hz), 128.63 (d, *J* = 7.0 Hz), 128.48, 114.36 (d, *J* = 4.6 Hz), 114.28 (d, *J* = 4.7 Hz), 55.21 (d, *J* = 2.4 Hz), 34.14 (d, *J* = 6.7 Hz) ppm; ^31^P NMR (162 MHz, CDCl_3_) δ −8.28 (m) ppm; IR (film): γ = 3004, 2939, 2835, 2531, 2044, 1893, 1593, 1567, 1497, 1463, 1441, 1402, 1381, 1285, 1247, 1177, 1095, 1030, 950, 893, 872, 826, 796, 733, 706, 657, 530 cm^−1^; HRMS (GC-TOF): calculated for C_56_H_51_O_4_P_2_^+^([M+H]^+^): *m*/*z* 849.3263, found: 849.3257.

*(S_p_)-1^2^,4^3^-bis(3-(bis(3,5-dimethylphenyl)phosphanyl)phenyl)-1,4(1,4)-dibenzenacyclohexaphane [(S_p_)-***5h***]*. 357 mg, 85% yield; white solid; M.p. 69–70 °C; [α${]}_{D}^{20}$ = 166.8° (c 1.00, DCM); ^1^H NMR (400 MHz, CDCl_3_) δ 7.30 (dd, *J* = 9.3, 7.5 Hz, 4H), 7.20 (dd, *J* = 7.6, 1.6 Hz, 2H), 6.98 (dd, *J* = 17.5, 8.1 Hz, 10H), 6.91–6.82 (m, 4H), 6.59 (d, *J* = 7.7 Hz, 2H), 6.52 (dd, *J* = 7.7, 1.5 Hz, 2H), 6.15 (d, *J* = 1.6 Hz, 2H), 3.33–3.18 (m, 2H), 3.02–2.88 (m, 2H), 2.82–2.70 (m, 2H), 2.42–2.33 (m, 2H), 2.21 (s, 12H), 2.15 (s, 12H) ppm; ^13^C NMR (101 MHz, CDCl_3_) δ 140.94 (d, *J* = 5.1 Hz), 140.08, 139.56, 137.98 (d, *J* = 5.8 Hz), 137.91 (d, *J* = 5.6 Hz), 137.59 (d, *J* = 11.5 Hz), 137.21 (d, *J* = 10.6 Hz), 136.91, 136.76, 135.29, 133.82 (d, *J* = 14.2 Hz), 132.31, 131.89 (d, *J* = 34.0 Hz), 131.50 (d, *J* = 4.0 Hz), 131.29, 130.57 (d, *J* = 13.6 Hz), 129.81 (d, *J* = 39.6 Hz), 128.59 (d, *J* = 8.6 Hz), 34.03 (d, *J* = 9.6 Hz), 21.26 (d, *J* = 5.2 Hz) ppm; ^31^P NMR (162 MHz, CDCl_3_) δ −4.82 (dt, *J* = 15.6, 7.8 Hz) ppm; IR (film): γ = 3024, 2921, 2857, 2730, 1889, 1784, 1742, 1598, 1582, 1464, 1415, 1379, 1264, 1168, 1125, 1105, 1040, 995, 893, 872, 847, 796, 733, 707, 693, 657, 579557, 503, 480 cm^−1^; HRMS (GC-TOF): calculated for C_60_H_59_P_2_^+^([M+H]^+^): *m*/*z* 841.4092, found: 841.4088.

#### 3.2.5. Synthesis of Compound (*S_p_*)-**8**

Under a nitrogen atmosphere, the solution of ligand (*S_p_*)-**5** (7.3 mg, 10 mol%) and [Pd(C_3_H_5_)Cl]_2_ (1.7 mg, 5 mol%) in toluene (1 mL) was stirred at room temperature for 1 h, and a solution of 1,3-diphenyl-2-propyl acetate (**6**) (0.1 mmol) in toluene (1.0 mL) was added. After 10 min, dialkyl malonate **7** (0.3 mmol) in toluene (1.0 mL) and Et_2_Zn (0.3 mmol, 1 M in hexane) were added to the mixture and the resulting solution was stirred at 0 °C for 12 h. The reaction mixture was diluted with ethyl acetate (10 mL) and washed with saturated aqueous ammonium chloride. The organic phase was separated and dried over anhydrous Na_2_SO_4_. *T*he organic layer was filtered, and the filtrate was concentrated under reduced pressure. The residue was purified by flash chromatography with petroleum ether/ethyl acetate = 20/1 to afford the corresponding product, **8**, as a colorless oil.

*diethyl (S,E)-2-(1,3-diphenylallyl)malonate (***8***)*. HPLC analysis: 64% *ee* (with (*S_p_*)-**5a**), Chiralpak IA, *i*-PrOH/*n*-hexane = 15/85, 1.0 mL/min, 254 nm; t_1_ = 6.8 min; t_2_ = 8.4 min; ^1^H NMR (400 MHz, CDCl_3_) δ 7.32–7.26 (m, 7H), 7.22 (dd, *J* = 16.4, 8.6 Hz, 3H), 6.48 (d, *J* = 15.8 Hz, 1H), 6.34 (dd, *J* = 15.7, 8.5 Hz, 1H), 4.30–4.22 (m, 1H), 4.17 (q, *J* = 7.1 Hz, 2H), 4.01–3.90 (m, 3H), 1.20 (t, *J* = 7.1 Hz, 3H), 1.01 (t, *J* = 7.1 Hz, 3H) ppm; ^13^C NMR (101 MHz, CDCl_3_) δ 167.87, 167.45, 140.32, 136.86, 131.69, 129.36, 128.67, 128.48, 128.01, 127.54, 127.12, 126.37, 61.61, 61.40, 57.80, 49.25, 14.16, 13.80; IR (film): γ = 3060, 2981, 1755, 1732, 1600, 1495, 1454, 1390, 1368, 1309, 1255, 1174, 1154, 1095, 1031, 966, 746, 698 cm^−1^; HRMS (GC-TOF): calculated for C_22_H_24_O_4_^+^([M]^+^): *m*/*z* 352.1675, found: 352.1674.

## 4. Conclusions

In summary, we developed a simple and scalable route for a new species of planar chiral bisphosphine ligands based on diphenyl [2.2]paracyclophane (PhPhanePHOS) in four steps and with good overall yields. PhPhanePHOS was tested in Pd-catalyzed asymmetric allylic alkylation reactions, with good yields and moderate enantioselectivity. Moreover, different positions of the phosphines, such as *o*-PhPhanePHOS and *m*-PhPhanePHOS, showed a great influence on the enantioselectivity of the reaction. Further application of these new planar chiral ligands in asymmetric catalysis is currently in progress.

## Data Availability

The original contributions presented in the study are included in the article/Supplementary Materials; further inquiries can be directed to the corresponding author.

## Supplementary Materials

The following supporting information can be downloaded at: https://www.mdpi.com/article/10.3390/molecules31030494/s1, ^1^H NMR, ^31^P NMR and ^13^C NMR spectra are included in Figures S1–S58.
