# Supplementary material for: Design and Synthesis of Planar Chiral Bisphosphine Ligands Based on Diphenyl [2.2]-Paracyclophane
Source: Molecules. 2026 Jan 31;31(3):494. doi: 10.3390/molecules31030494 (PMC12899815; doi:10.3390/molecules31030494)
Supplement: Supplementary file 1 [file molecules-31-00494-s001.zip › molecules-4085481-supplementary.pdf]

Electronic Supplementary Information

Design and Synthesis of Planar Chiral Bisphosphine Ligands Based on Diphenyl [2.2]-Paracyclophane

Shaoying Huang, Yingjie Huang, Jiaping Jin, Haorui Gu* and Xufeng Lin *

Department of Chemistry, Zhejiang University, Hangzhou 310058, China; 12137005@zju.edu.cn (S. H.); 22337015@zju.edu.cn (Y.H.); jinjiaping@cirs-group.com (J.J.)
*Correspondence: 0019529@zju.edu.cn (H.G.); lxfok@zju.edu.cn (X.L.)

Table of Contents

1. ^{1}H, ^{13}C and ^{31}P NMR spectra S2
2. HPLC data S31
3. references S32

Academic Editors: Antonio Massa and Jacek Skarzewski

Received: 21 December 2025
Revised: 27 January 2026
Accepted: 29 January 2026
Published: 31 January 2026

Copyright: © 2026 by the authors. Licensee MDPI, Basel, Switzerland. This article is an open access article distributed under the terms and conditions of the Creative Commons Attribution (CC BY) license.

1. ^{1}H, ^{13}C and ^{31}P NMR spectra

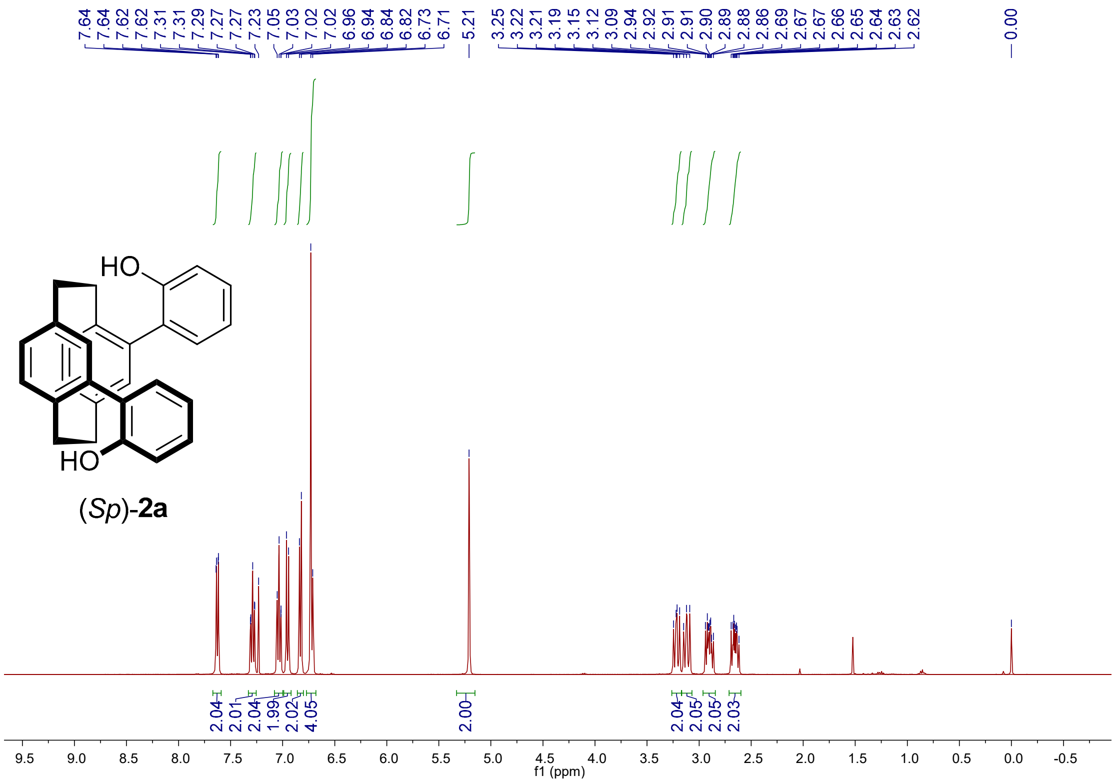

Figure S1. ^{1}H NMR (400 MHz, $CDCl_3$) spectrum of **(S_p)-2a**.

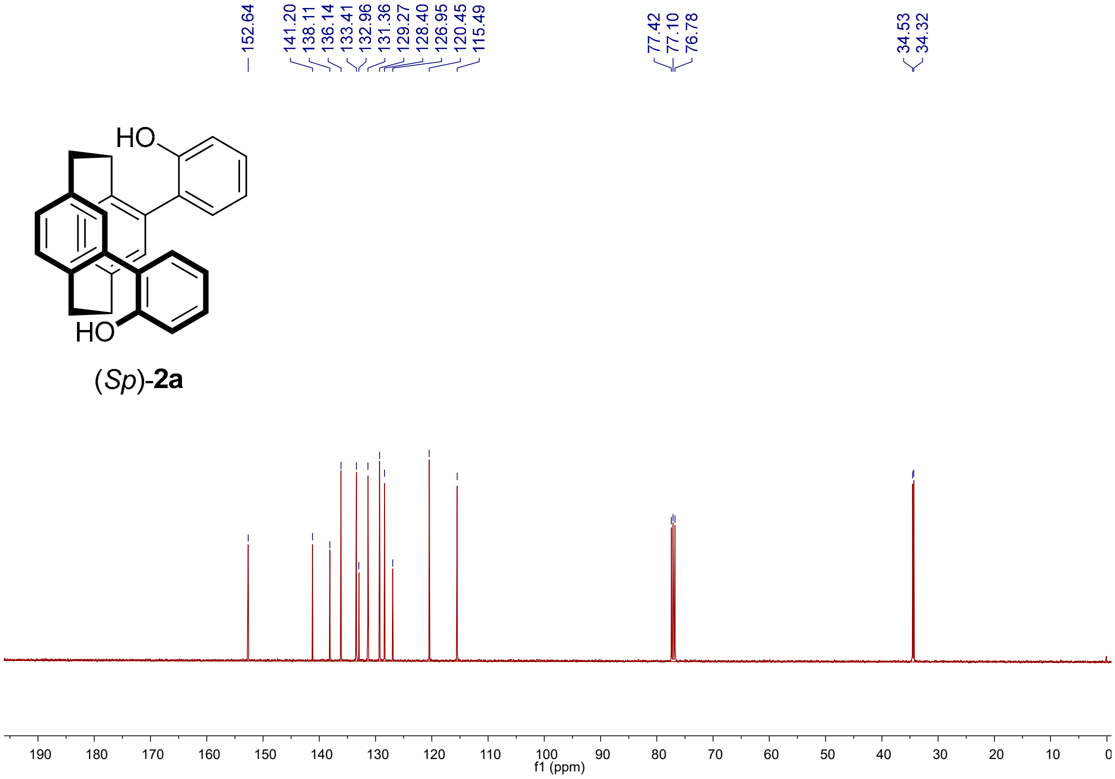

Figure S2. ^{13}C NMR (101 MHz, $CDCl_3$) spectrum of **(S_p)-2a**.

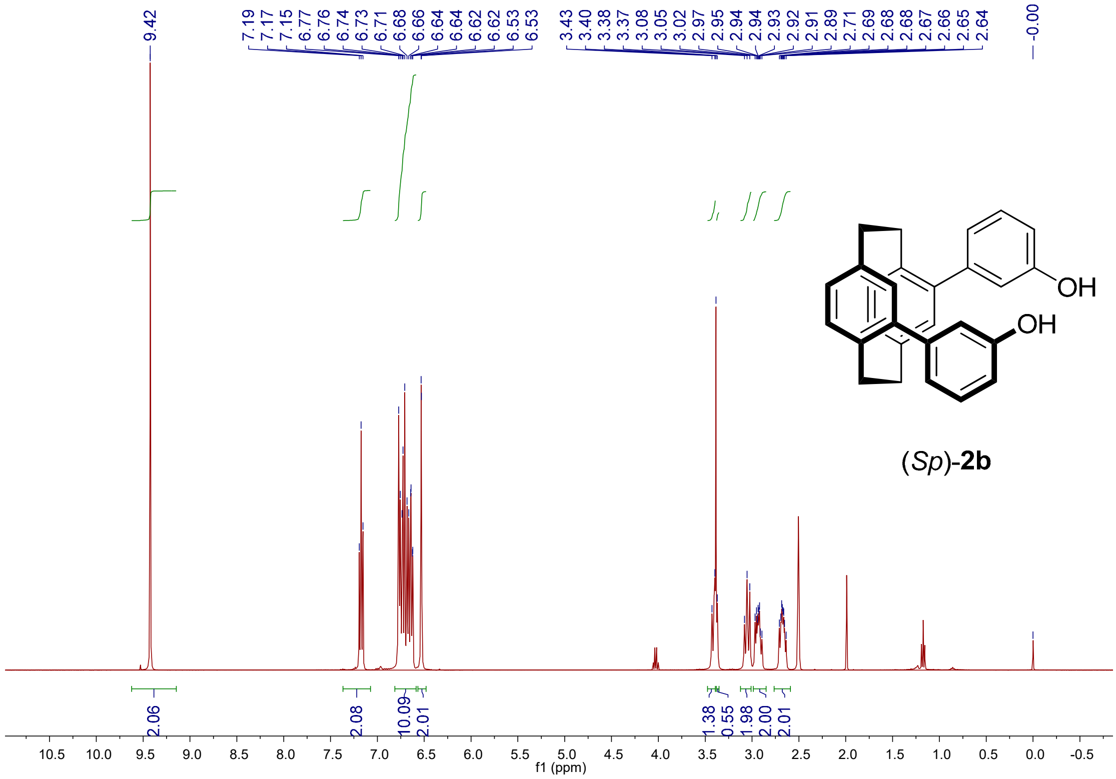

Figure S3. ^{1}H NMR (400 MHz, $CDCl_3$) spectrum of **(S_p)-2b**.

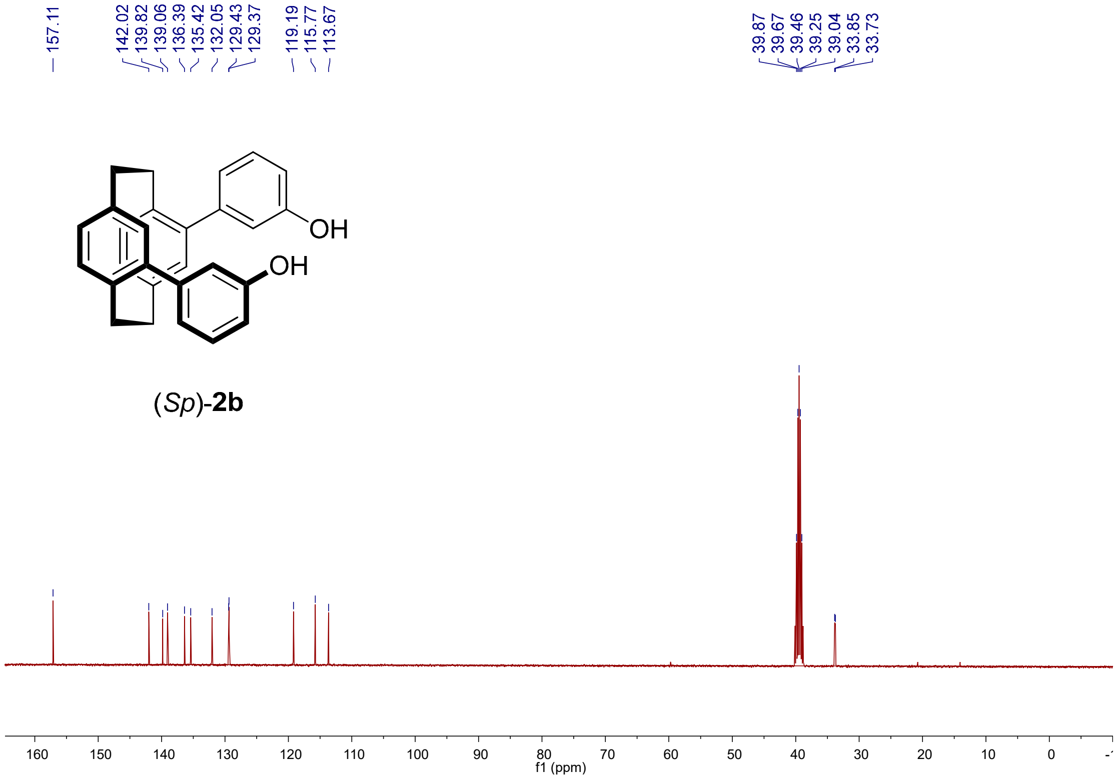

Figure S4. ^{13}C NMR (101 MHz, $CDCl_3$) spectrum of **(S_p)-2b**.

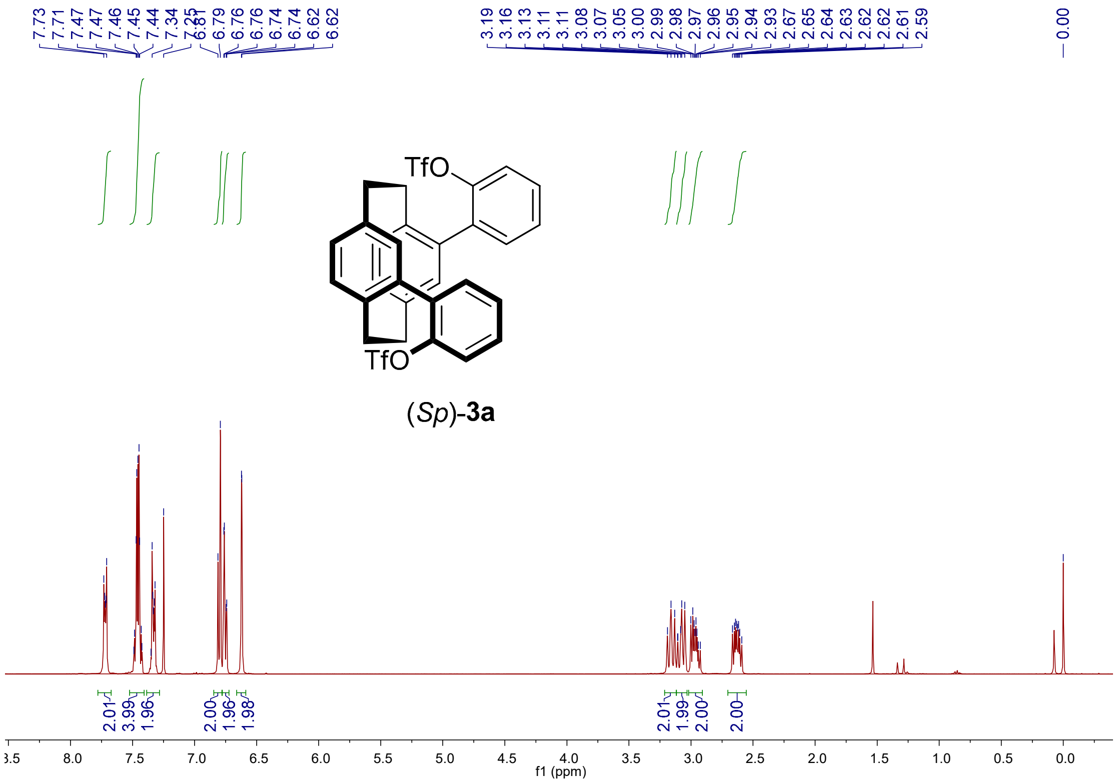

Figure S5. ^{1}H NMR (400 MHz, $CDCl_3$) spectrum of **(*S*$_p$)-3a**.

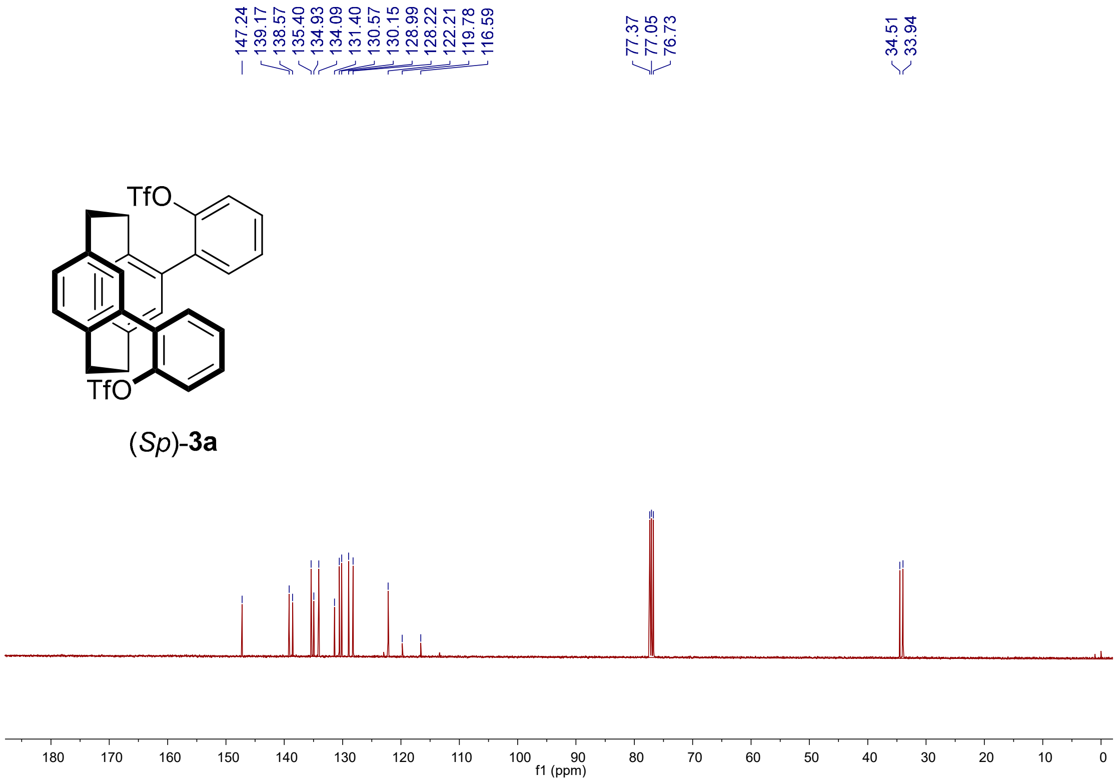

Figure S6. ^{13}C NMR (101 MHz, $CDCl_3$) spectrum of **(*S*$_p$)-3a**.

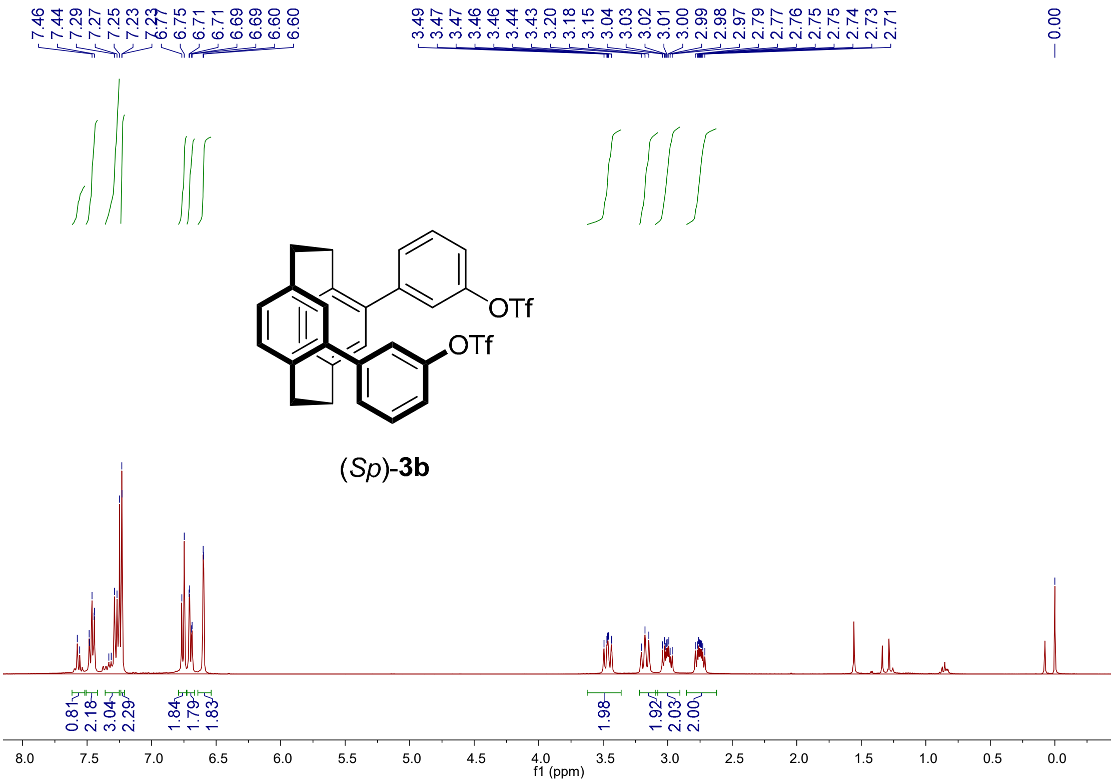

Figure S7. ^{1}H NMR spectrum of **(*S*$_p$)-3b**.

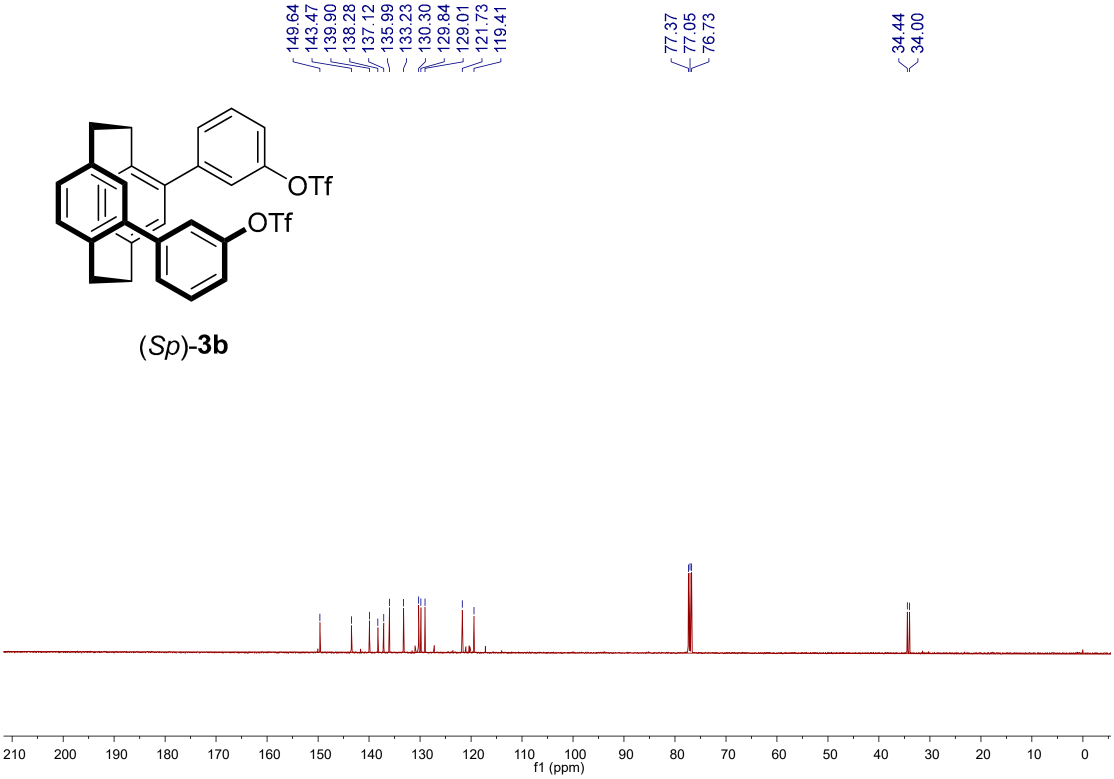

Figure S8. ^{13}C NMR (101 MHz, $CDCl_3$) spectrum of **(*S*$_p$)-3b**.

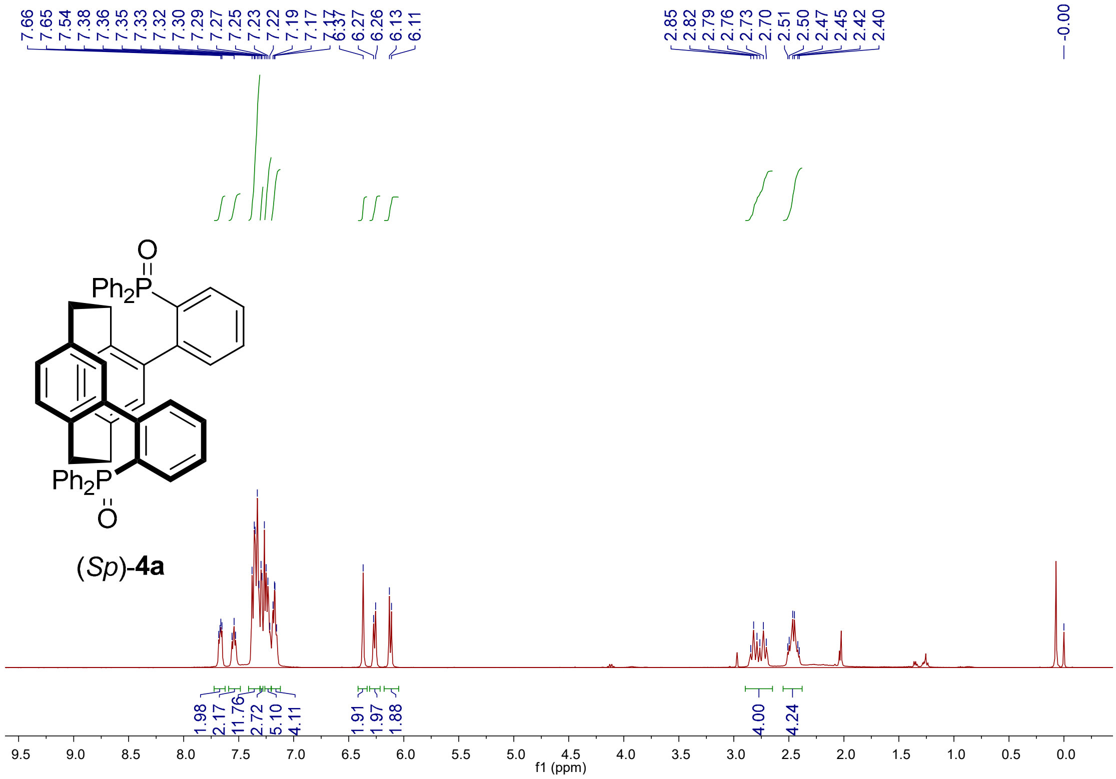

Figure S9. ^{1}H NMR (400 MHz, $CDCl_3$) spectrum of **(S_p)-4a**

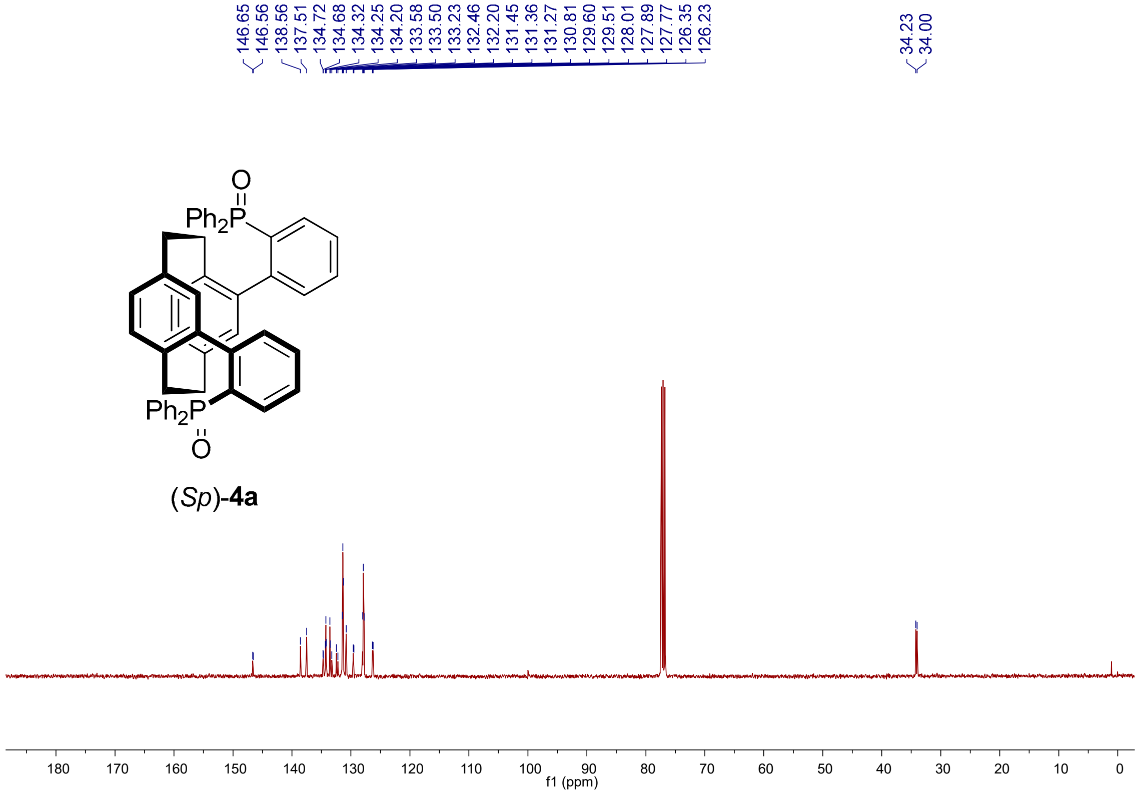

Figure S10. ^{13}C NMR (101 MHz, $CDCl_3$) spectrum of **(S_p)-4a**.

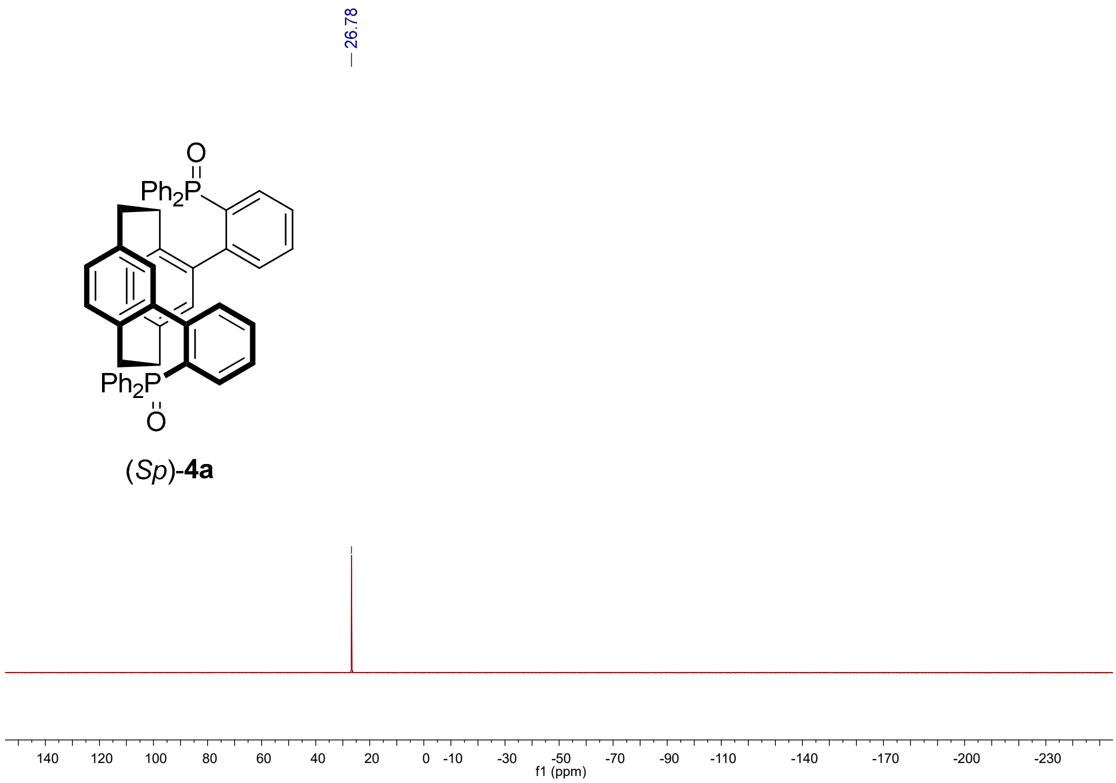

Figure S11. ^{31}P NMR (162 MHz, $CDCl_3$) spectrum of **(S_p)-4a**

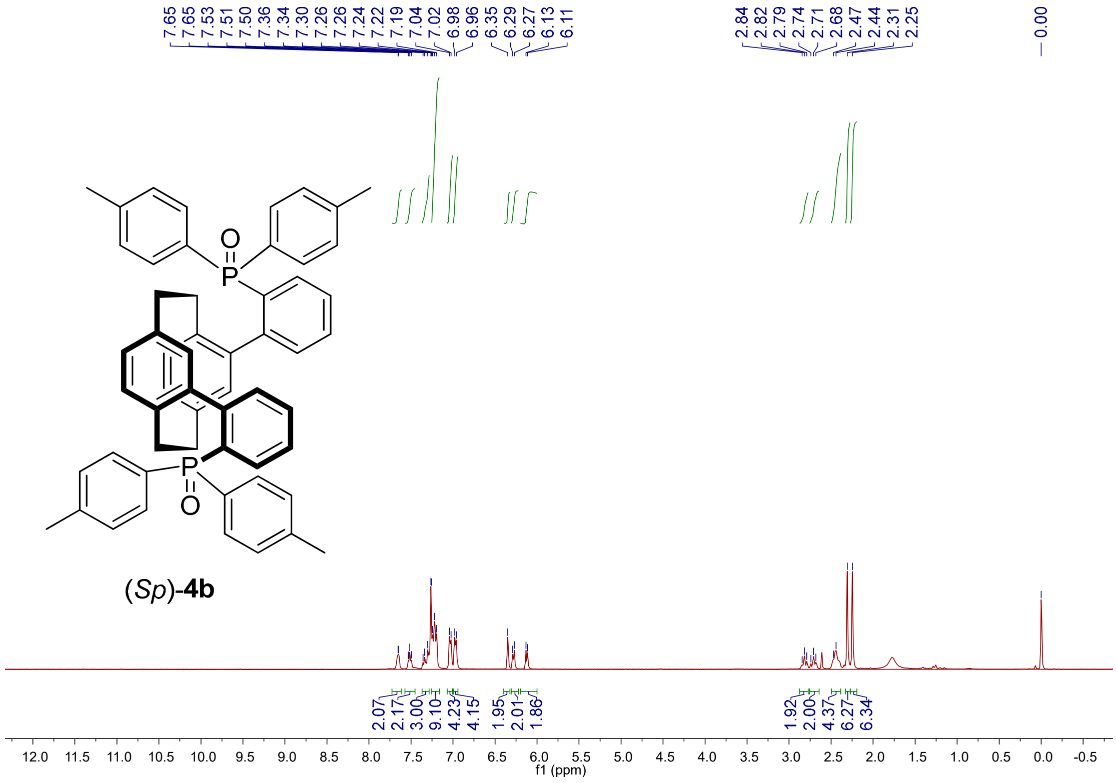

Figure S12. ^{1}H NMR (400 MHz, $CDCl_3$) spectrum of **(S_p)-4b**

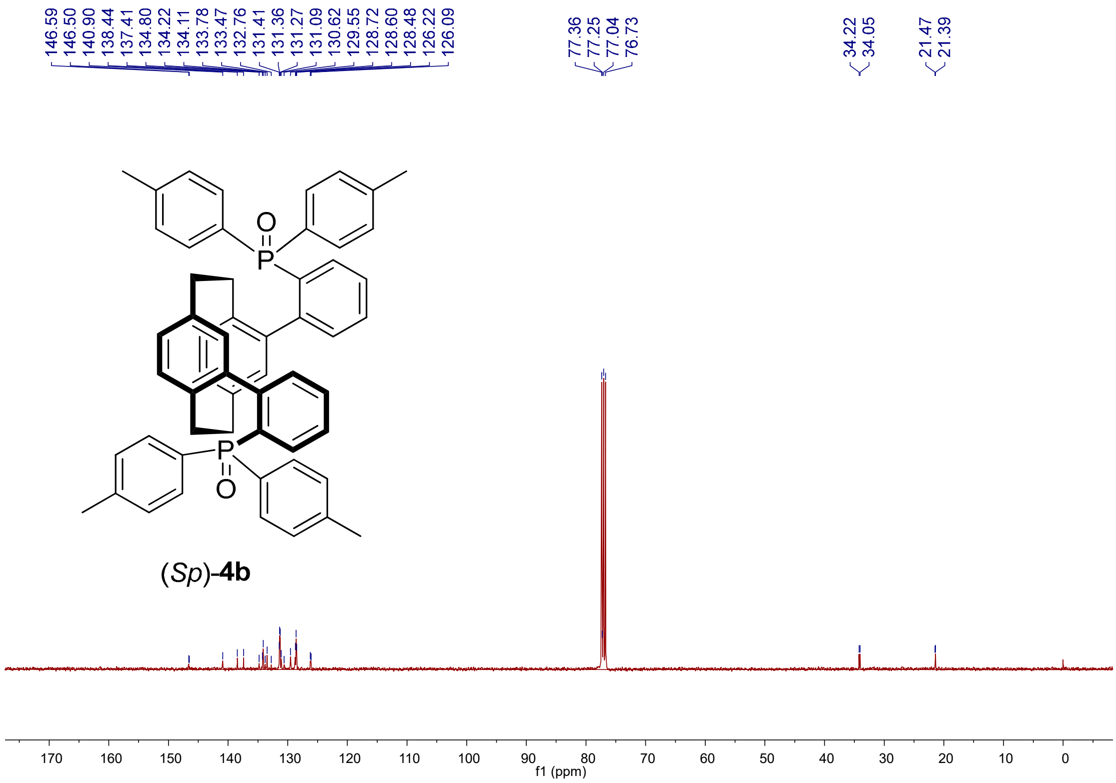

Figure S13. ^{13}C NMR (101 MHz, $CDCl_3$) spectrum of **(S_p)-4b**

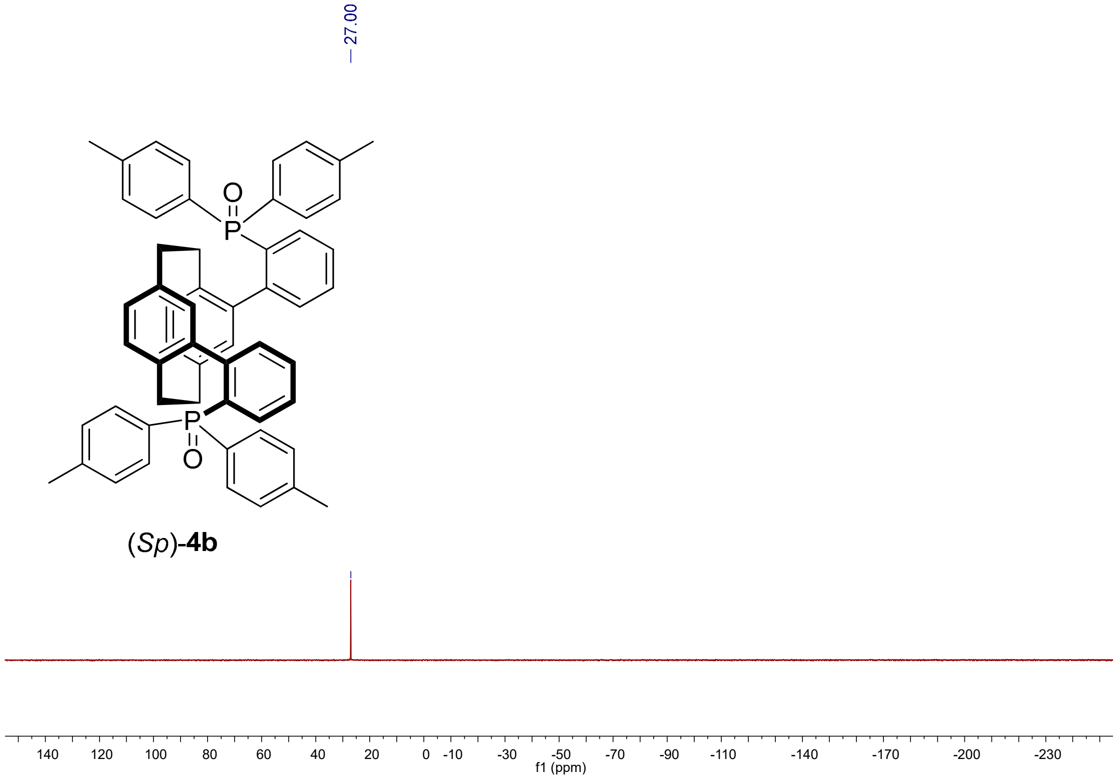

Figure S14. ^{31}P NMR (162 MHz, $CDCl_3$) spectrum of **(S_p)-4b**

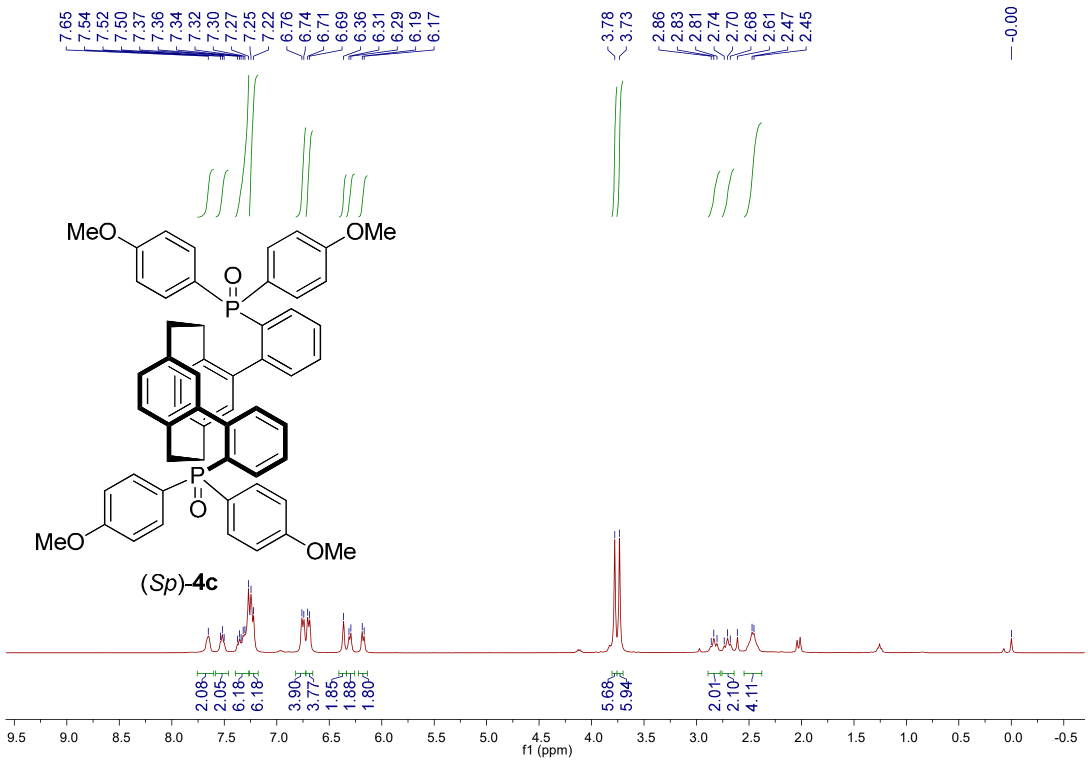

Figure S15. ^{1}H NMR (400 MHz, $CDCl_3$) spectrum of **(S_p)-4c**

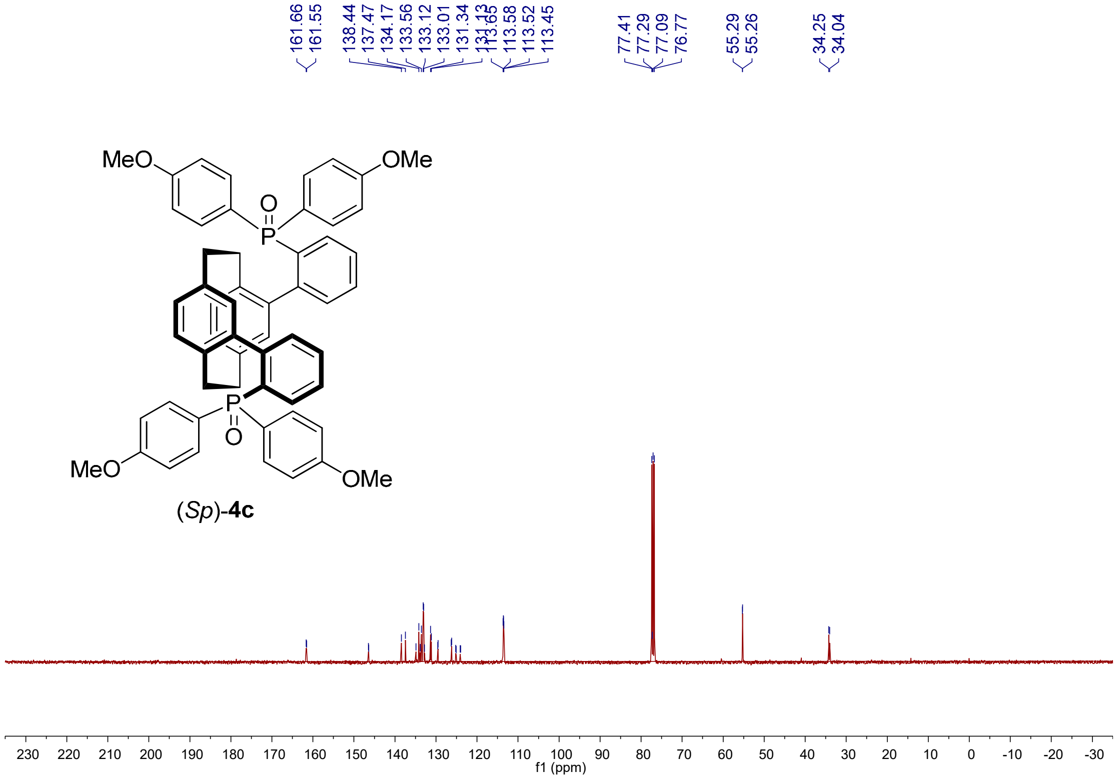

Figure S16. ^{13}C NMR (101 MHz, $CDCl_3$) spectrum of **(S_p)-4c**

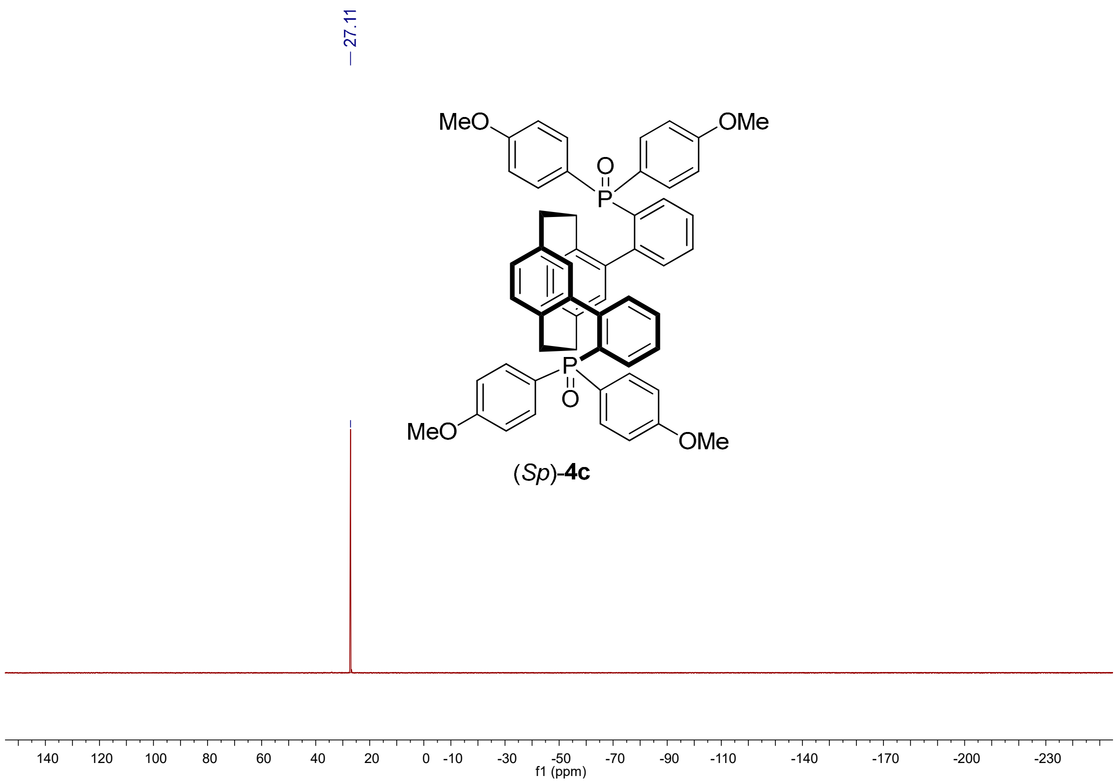

Figure S17. ^{31}P NMR (162 MHz, $CDCl_3$) spectrum of **(S_p)-4c**

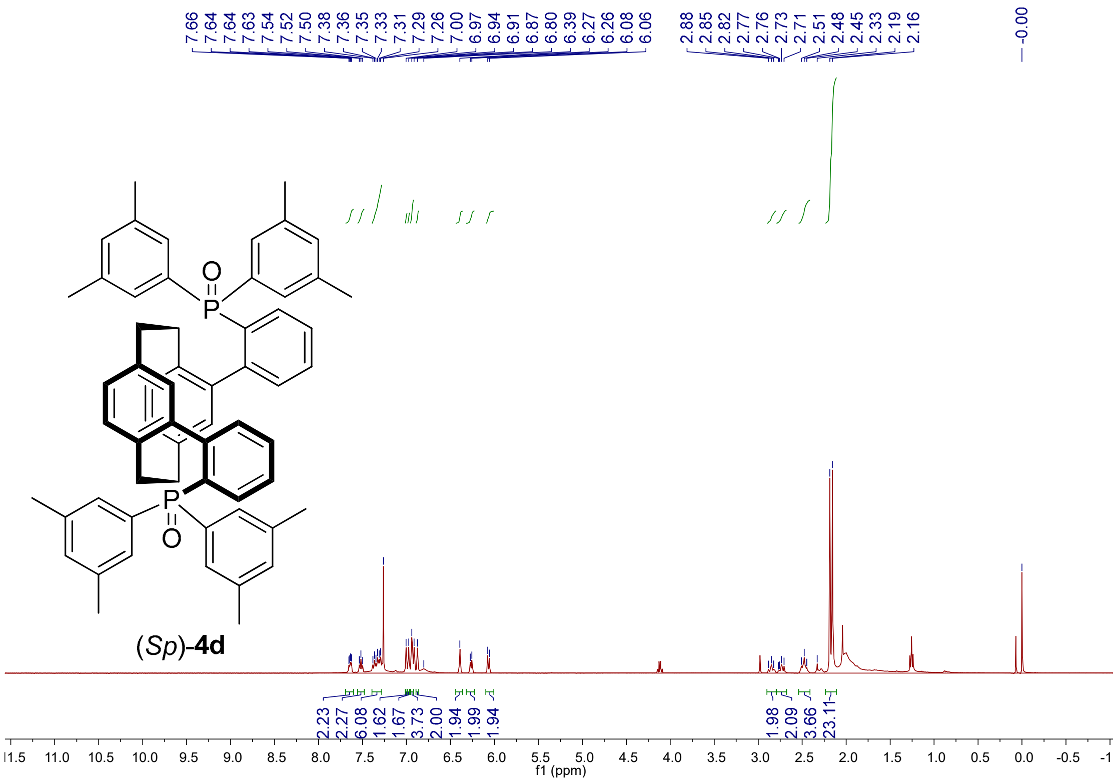

Figure S18. ^{1}H NMR (400 MHz, $CDCl_3$) spectrum of **(S_p)-4d**

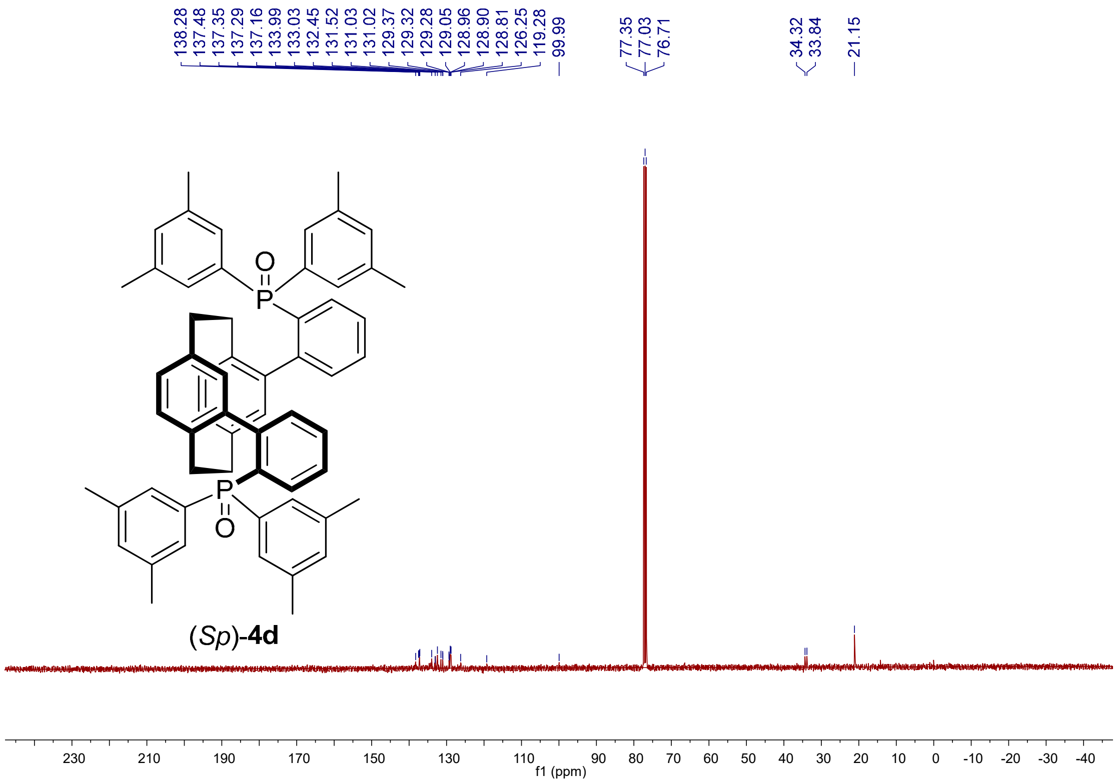

Figure S19. ^{13}C NMR (101 MHz, $CDCl_3$) spectrum of **(S_p)-4d**

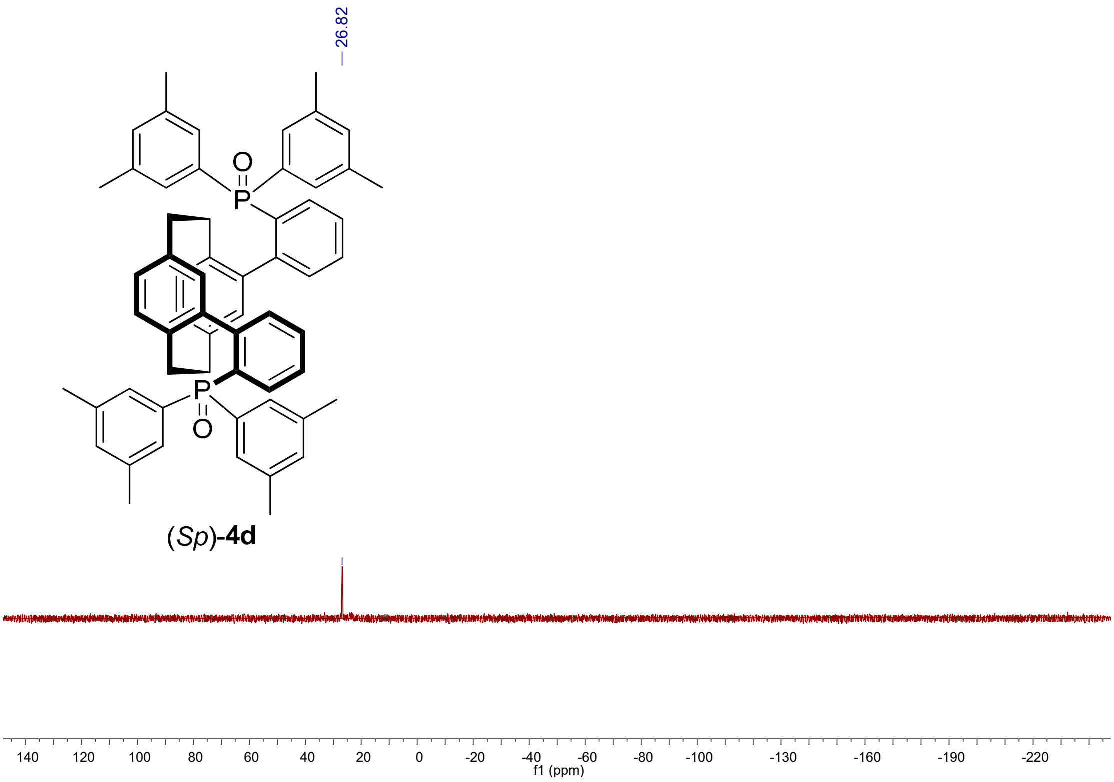

Figure S20. ^{31}P NMR (162 MHz, $CDCl_3$) spectrum of **(S_p)-4d**

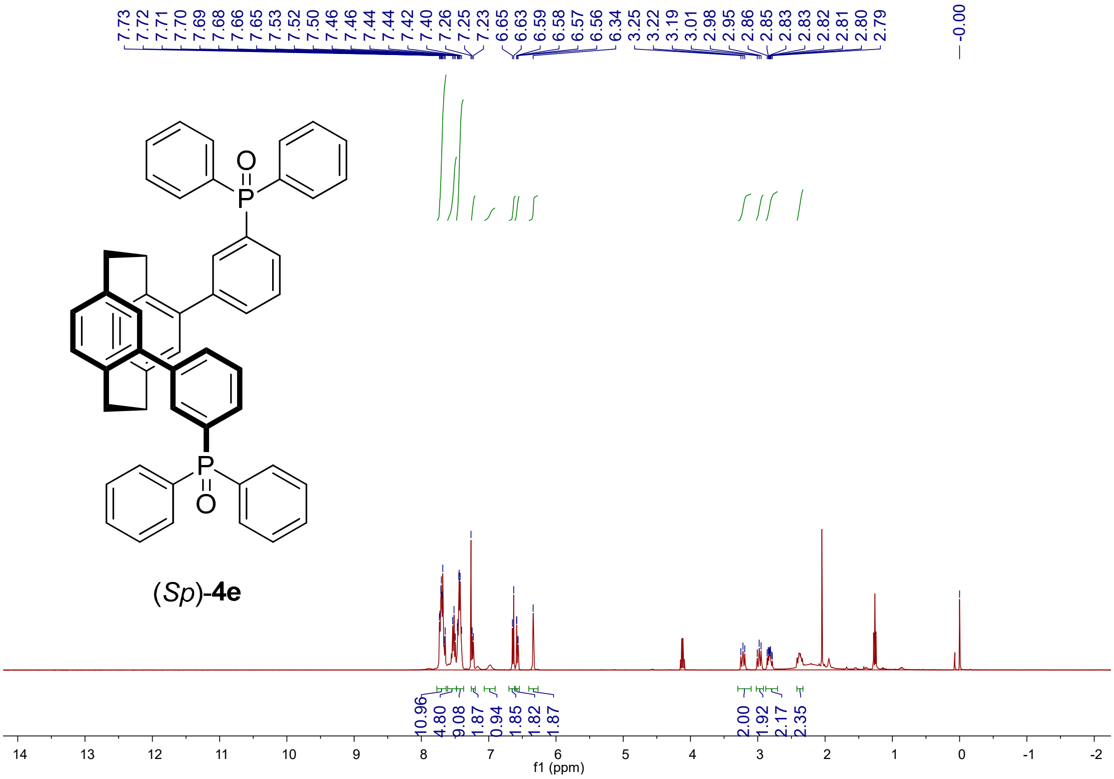

Figure S21. ^{1}H NMR (400 MHz, $CDCl_3$) spectrum of **(S_p)-4e**

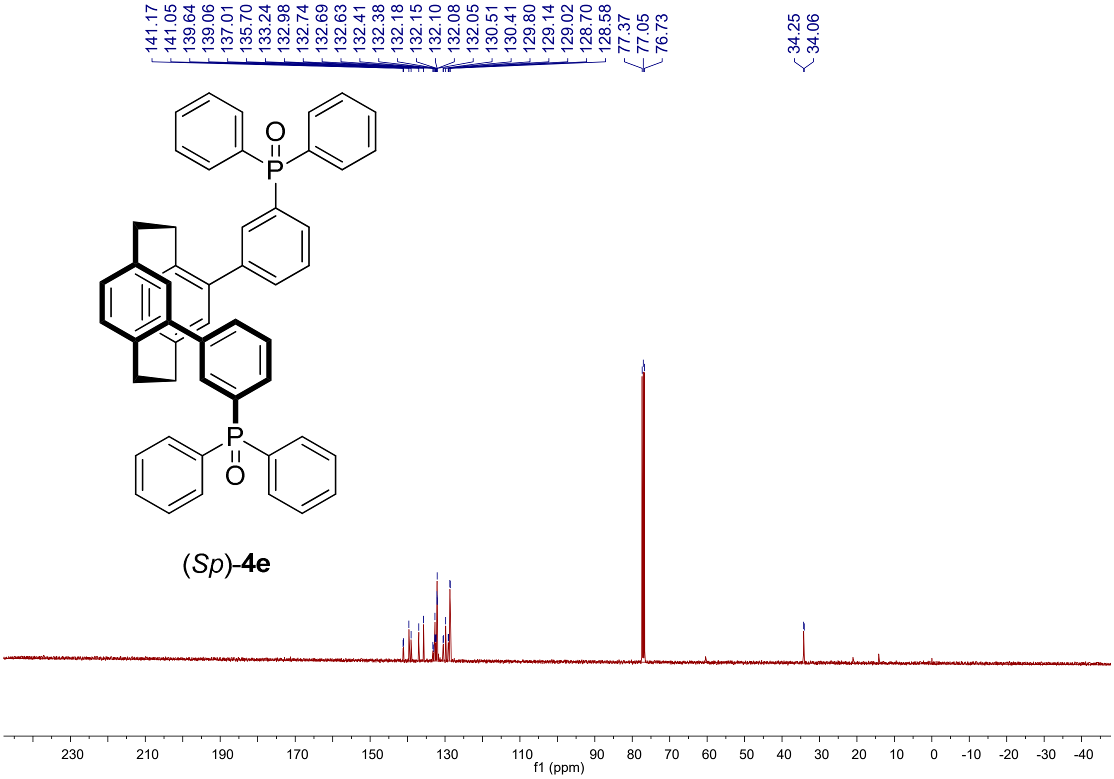

Figure S22. ^{13}C NMR (101 MHz, $CDCl_3$) spectrum of **(S_p)-4e**

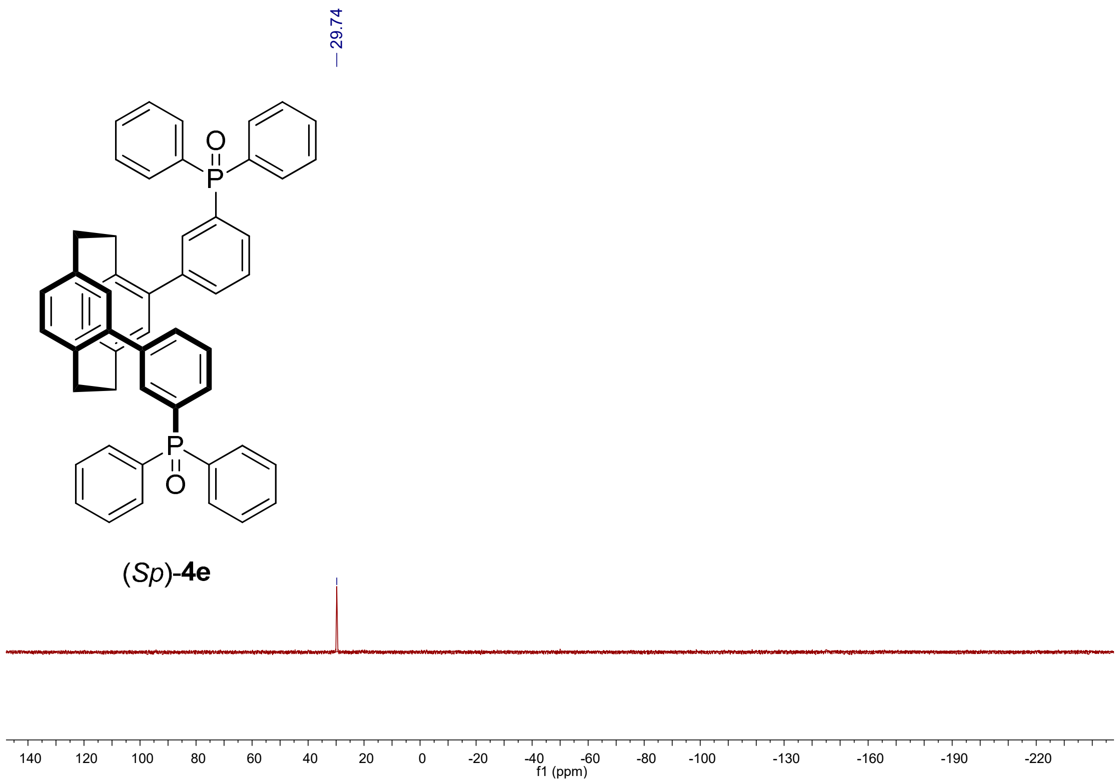

Figure S23. ^{31}P NMR (162 MHz, $CDCl_3$) spectrum of **(S_p)-4e**

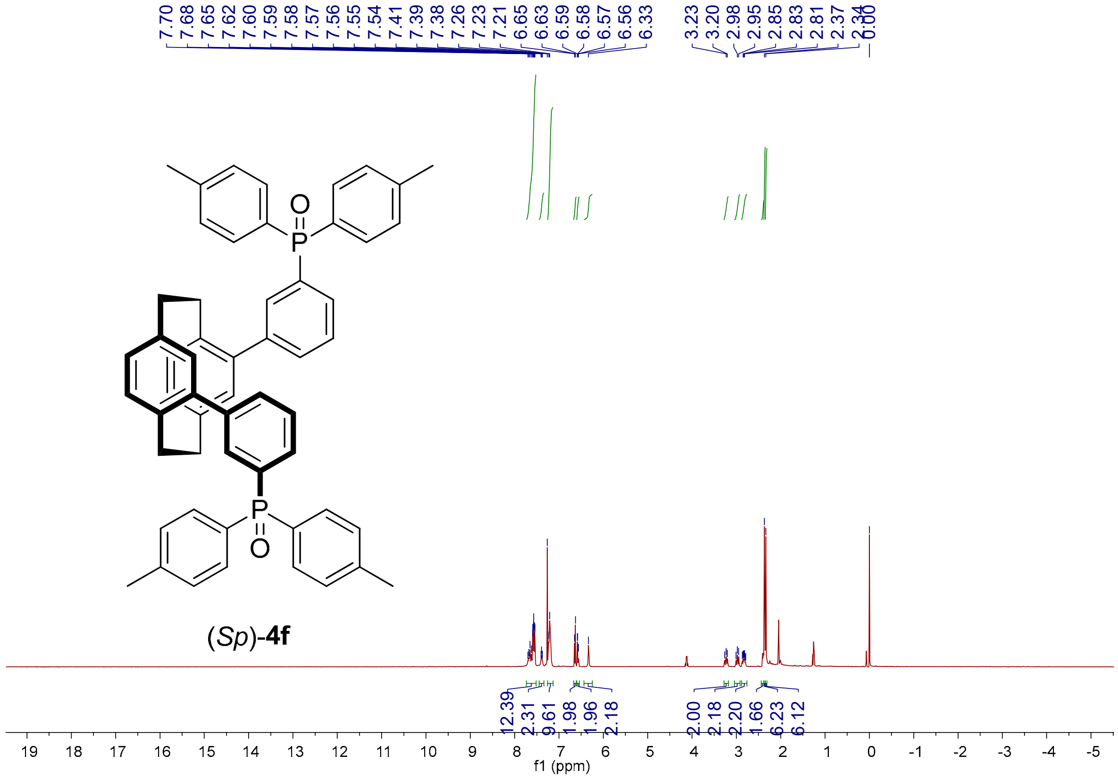

Figure S24. ^{1}H NMR (400 MHz, $CDCl_3$) spectrum of **(S_p)-4f**

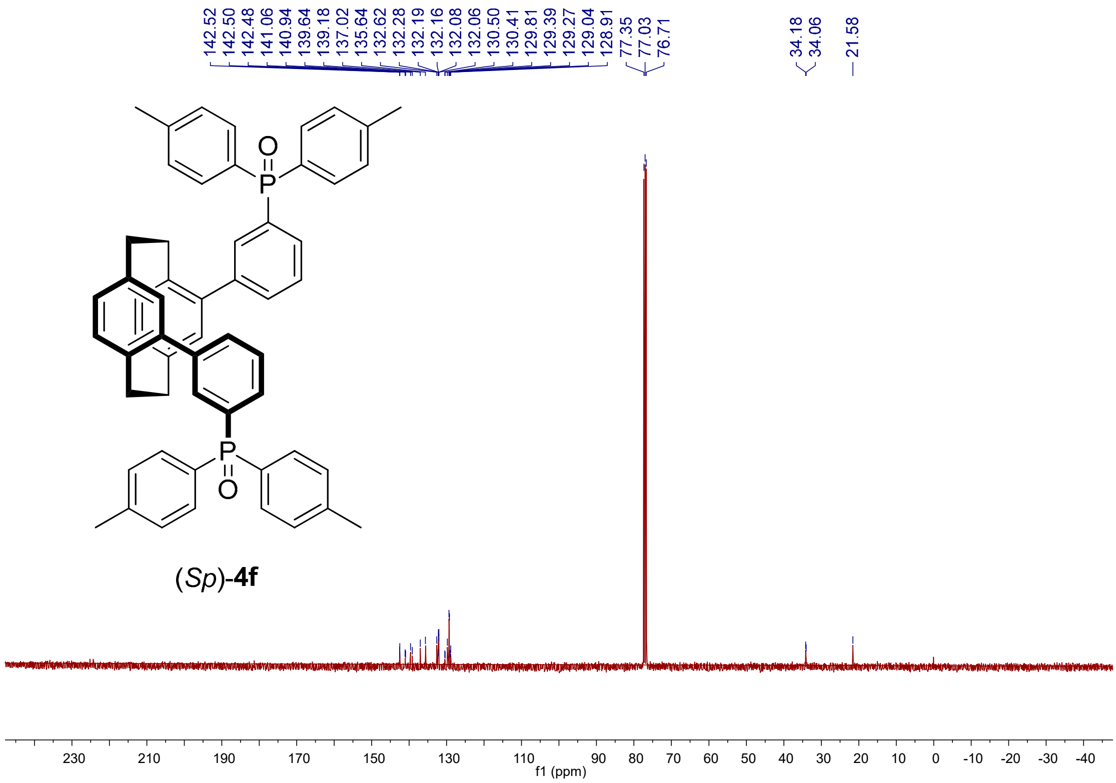

Figure S25. ^{13}C NMR (101 MHz, CDCl$_3$) spectrum of **(*S*$_p$)-4f**

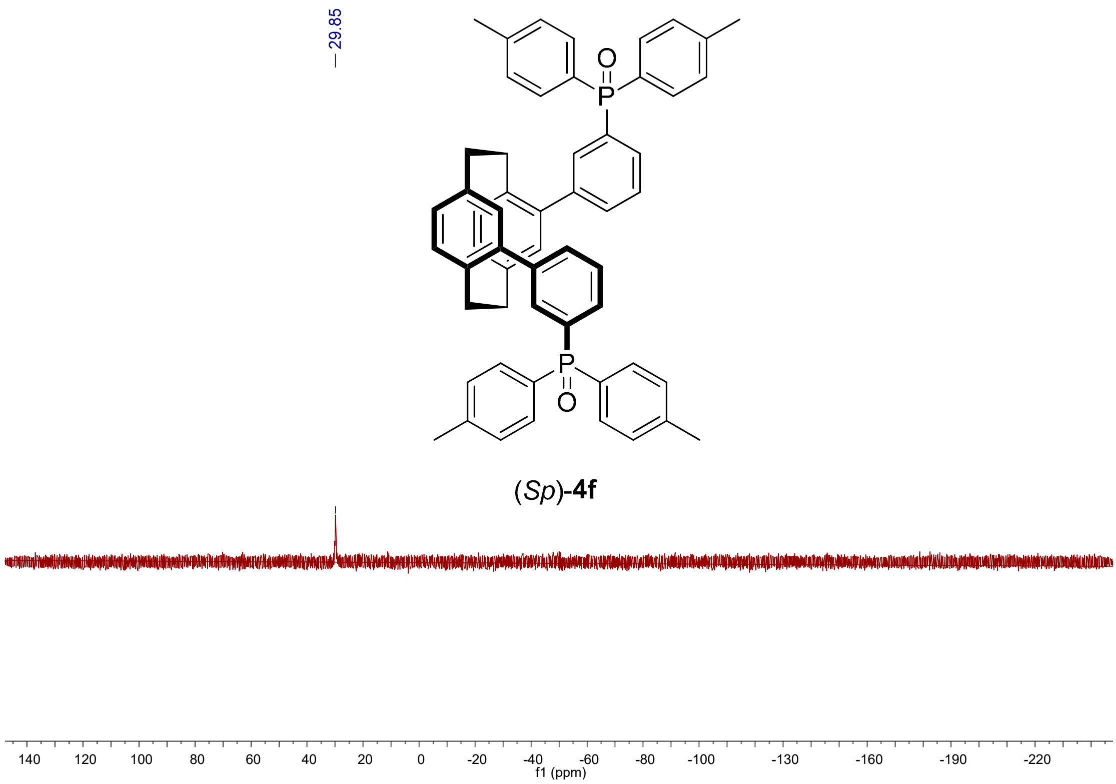

Figure S26. ^{31}P NMR (162 MHz, CDCl$_3$) spectrum of **(*S*$_p$)-4f**

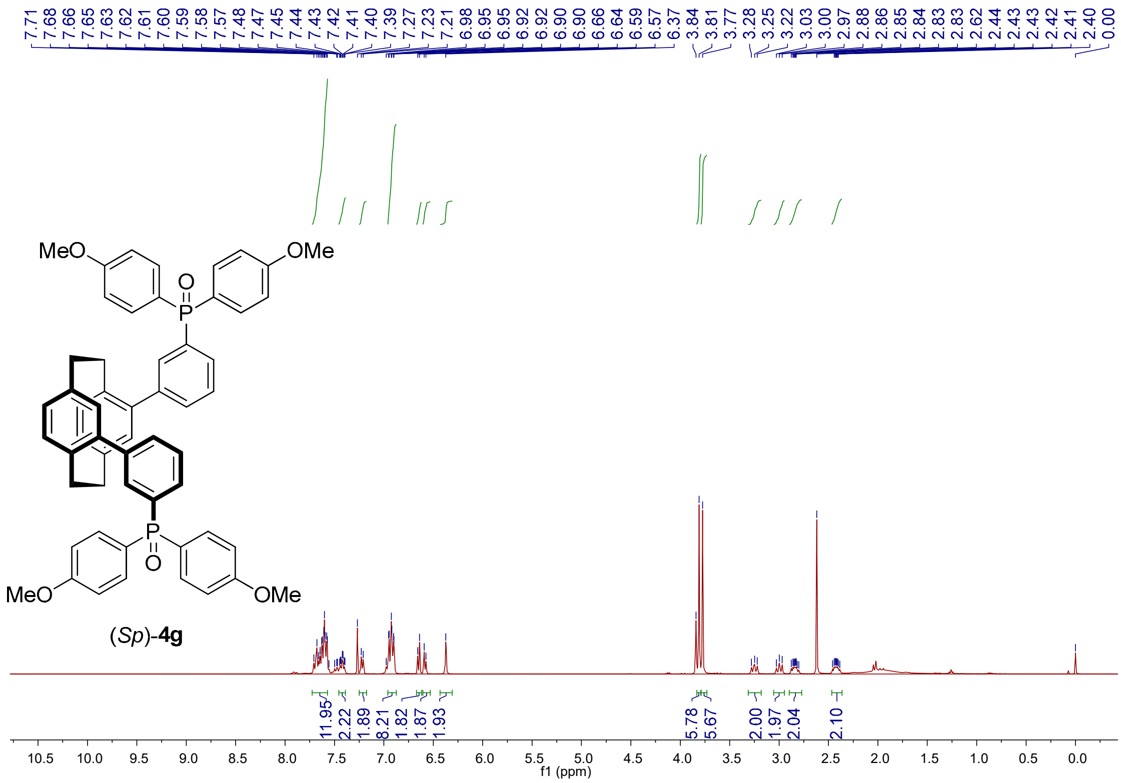

Figure S27. ^{1}H NMR (400 MHz, $CDCl_3$) spectrum of **(S_p)-4g**

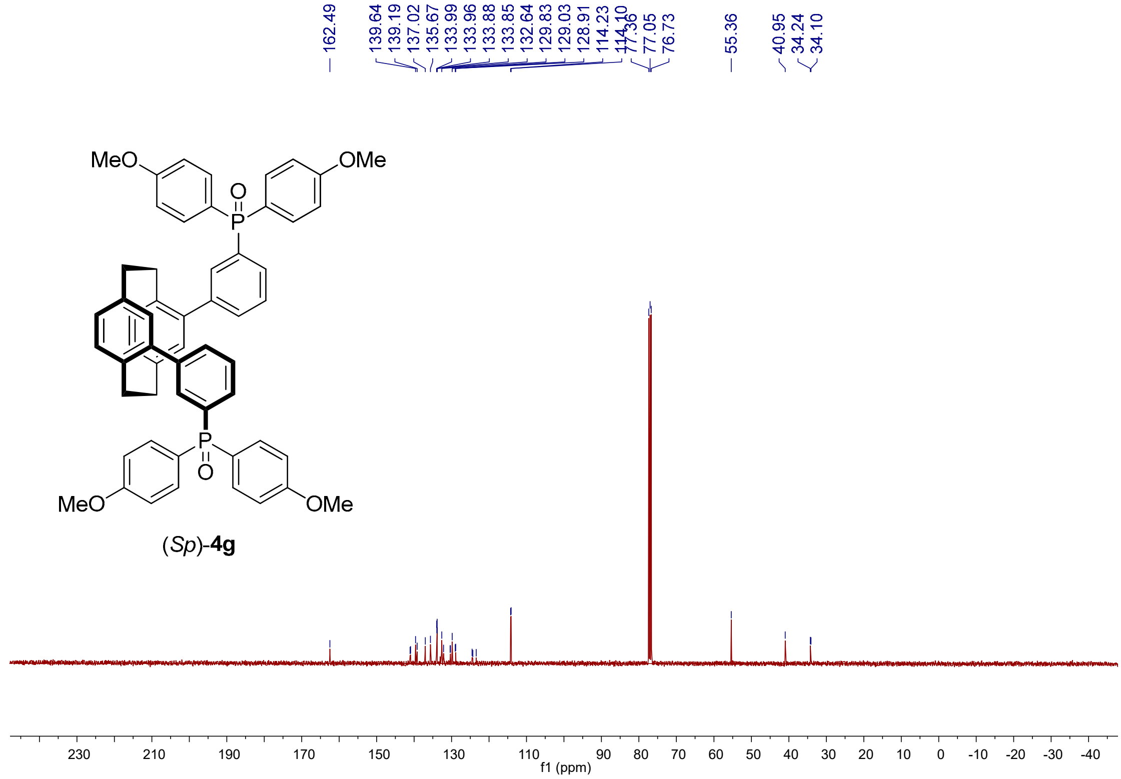

Figure S28. ^{13}C NMR (101 MHz, $CDCl_3$) spectrum of **(S_p)-4g**

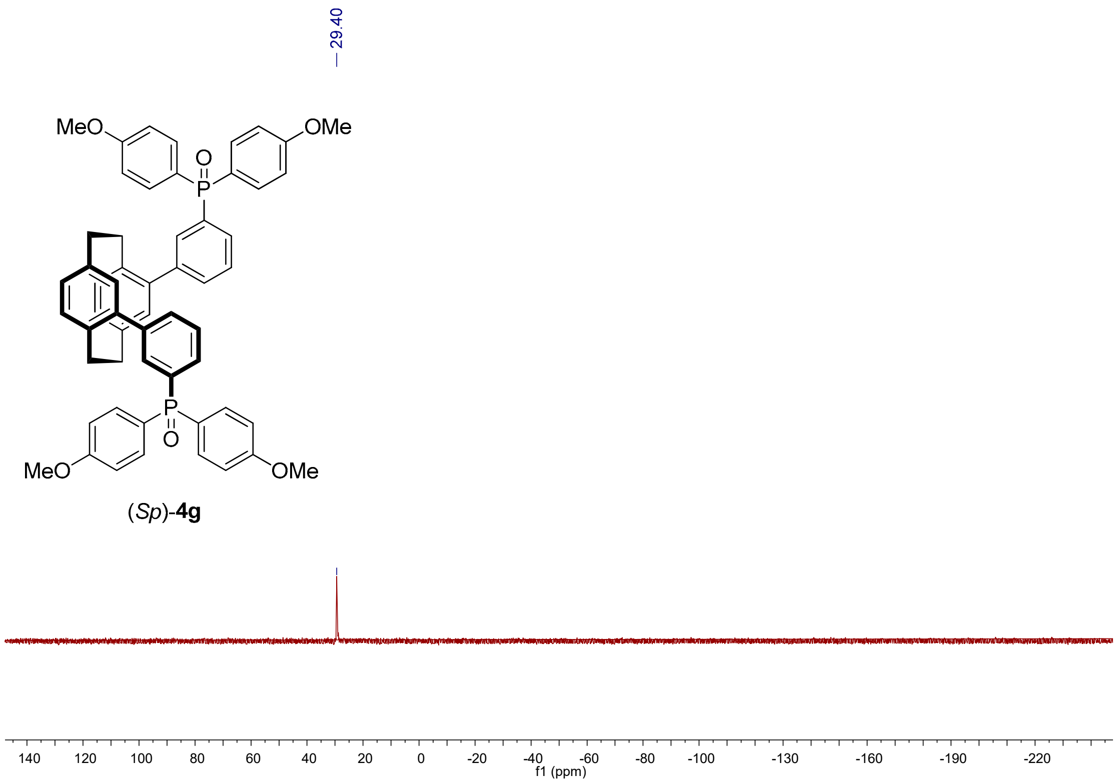

Figure S29. ^{31}P NMR (162 MHz, CDCl$_3$) spectrum of **(S_p)-4g**

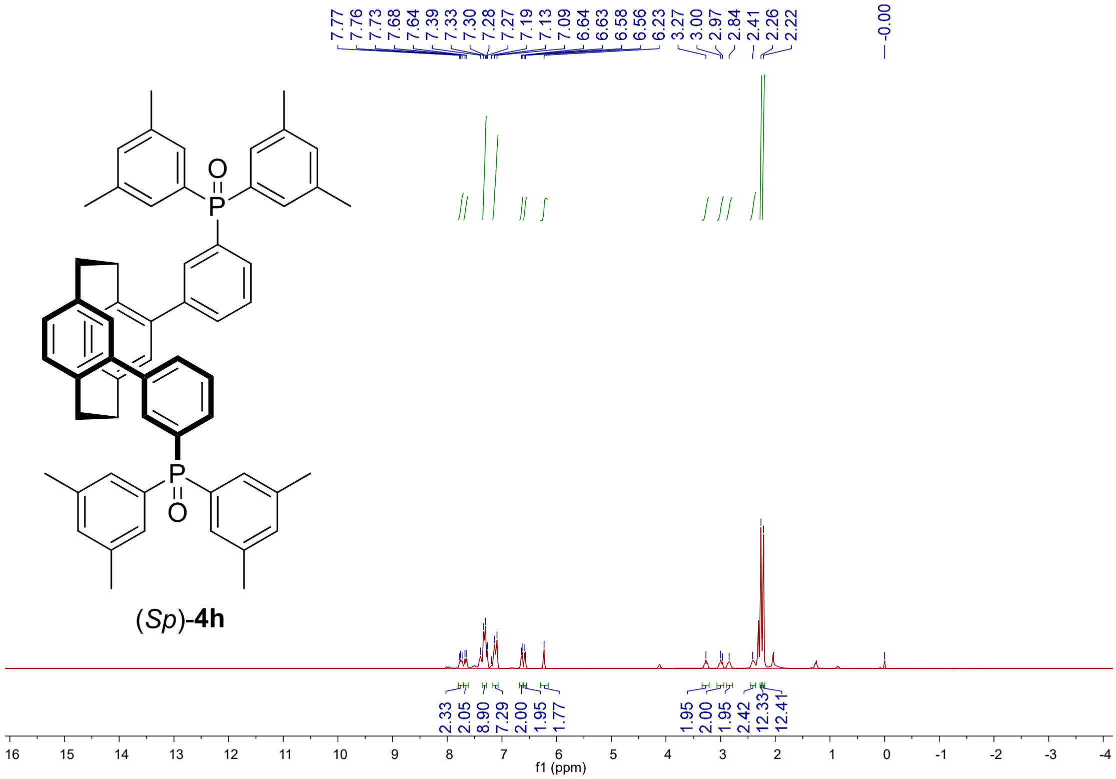

Figure S30. ^{1}H NMR (400 MHz, CDCl$_3$) spectrum of **(S_p)-4h**

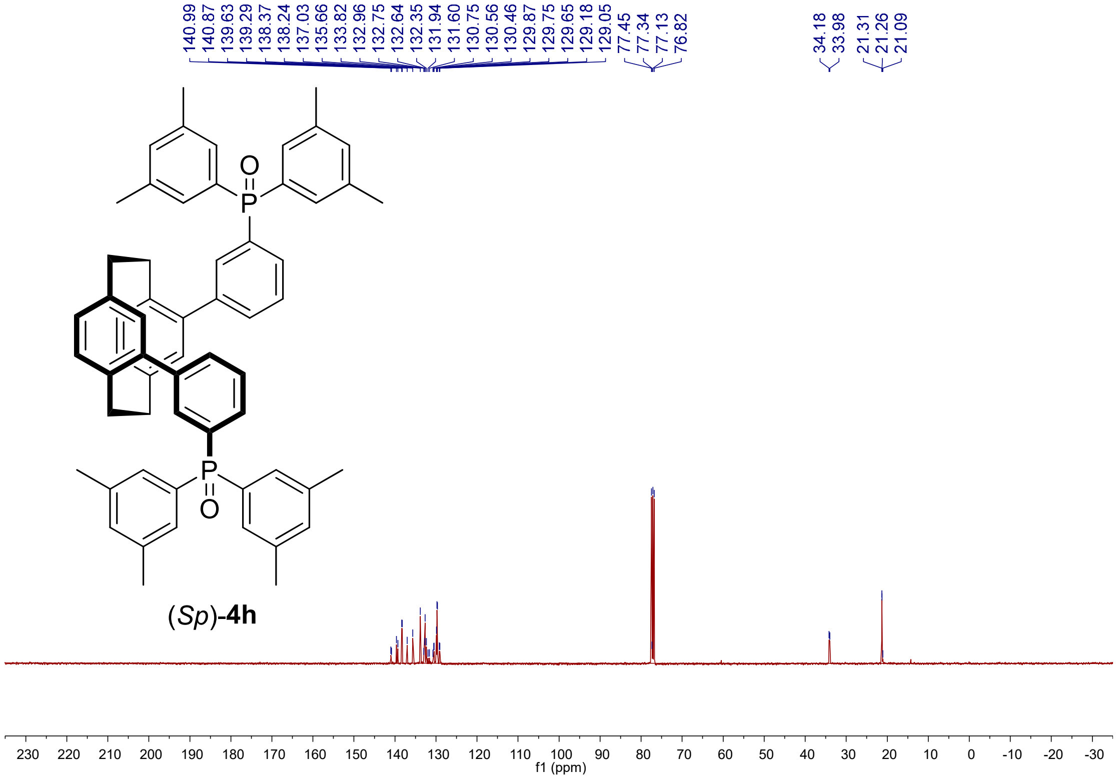

Figure S31. ^{13}C NMR (101 MHz, $CDCl_3$) spectrum of **(S_p)-4h**

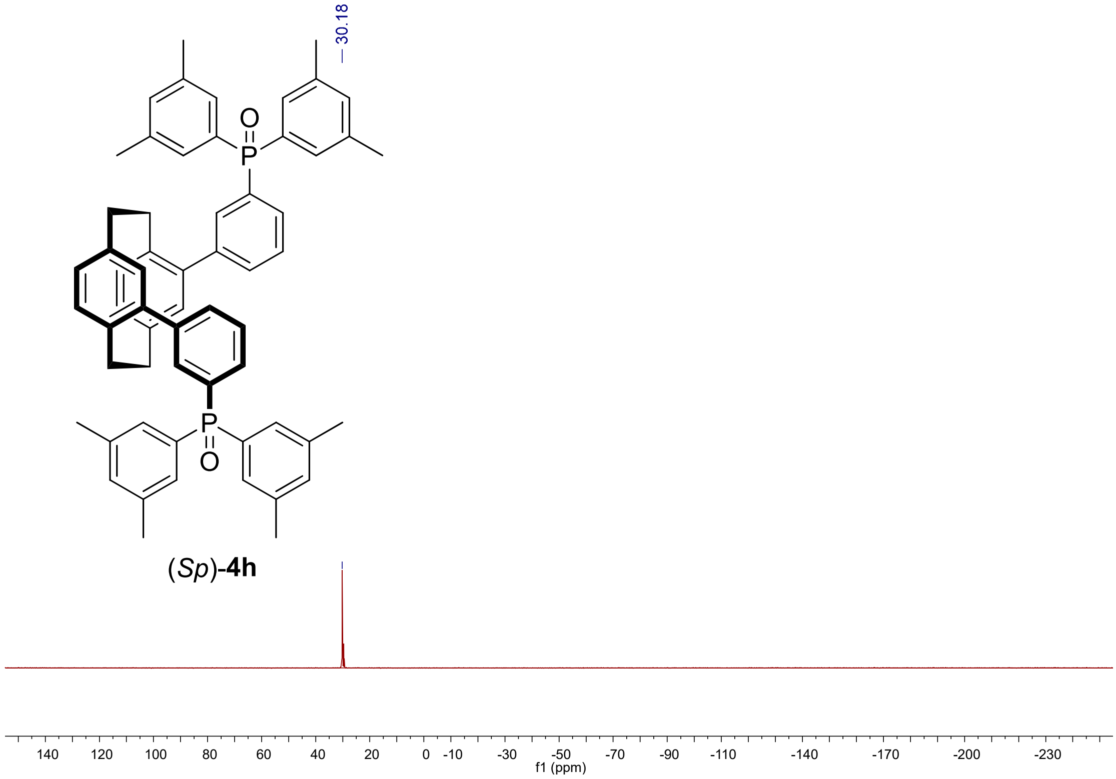

Figure S32. ^{31}P NMR (162 MHz, $CDCl_3$) spectrum of **(S_p)-4h**

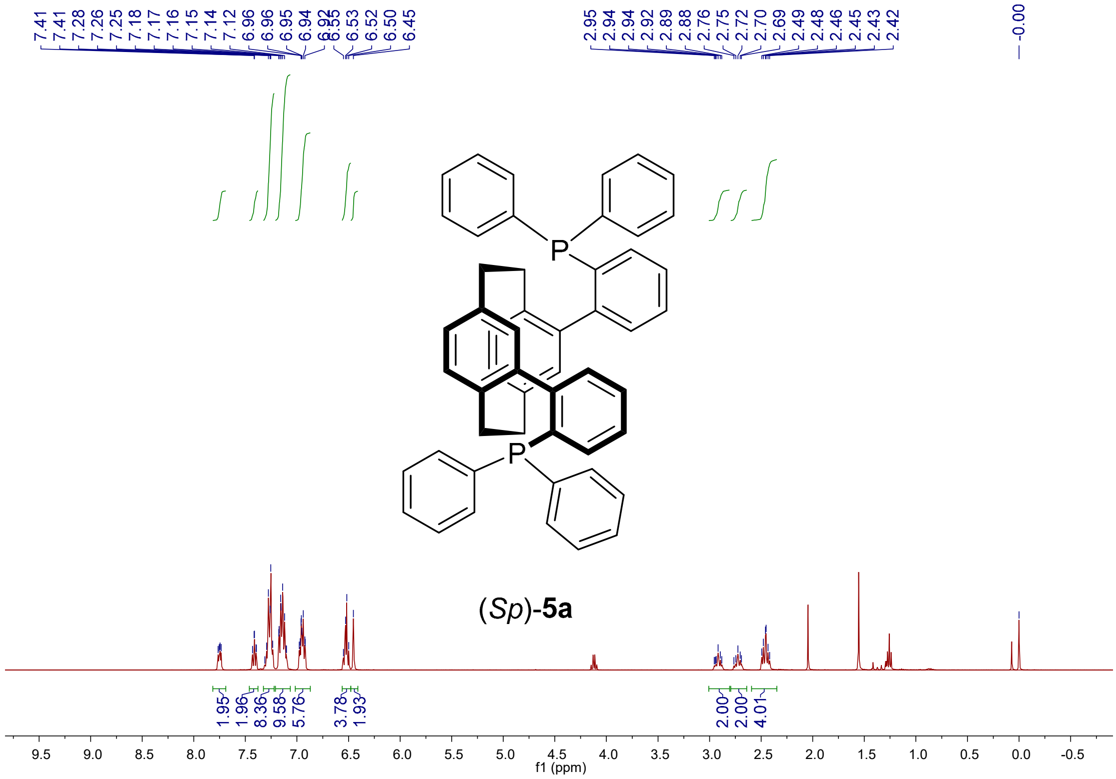

Figure S33. ^{1}H NMR (400 MHz, $CDCl_3$) spectrum of **(*S_p*)-5a**

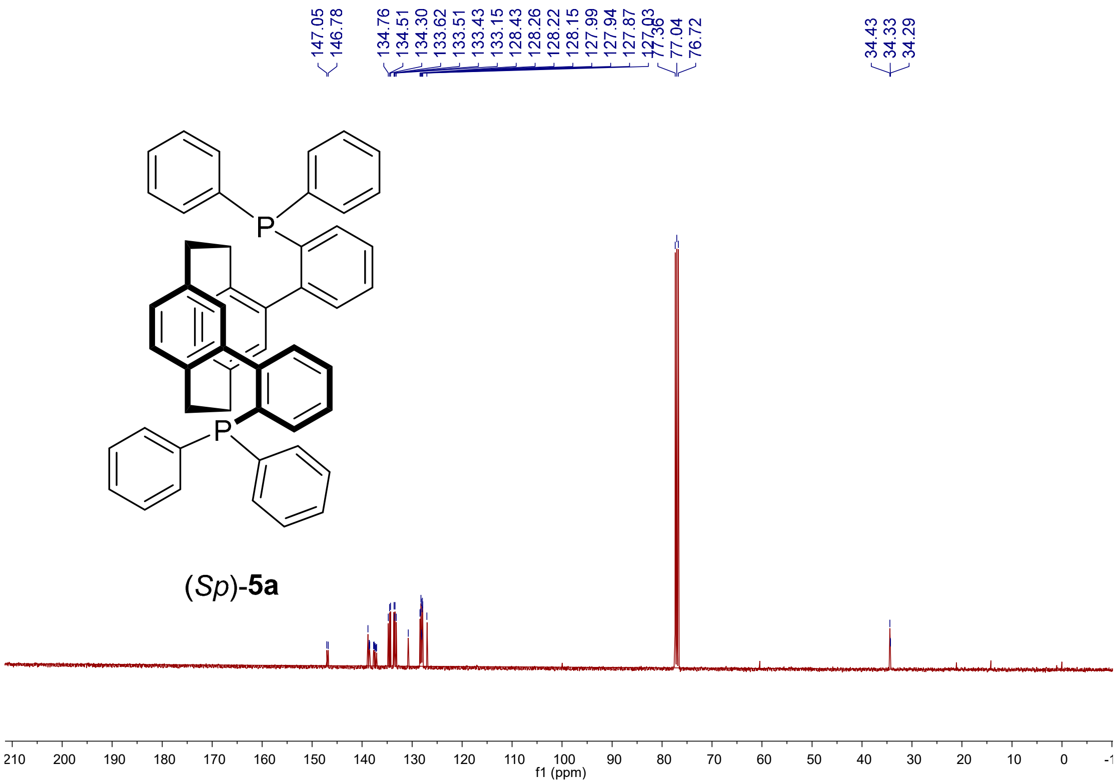

Figure S34. ^{13}C NMR (101 MHz, $CDCl_3$) spectrum of **(*S_p*)-5a**

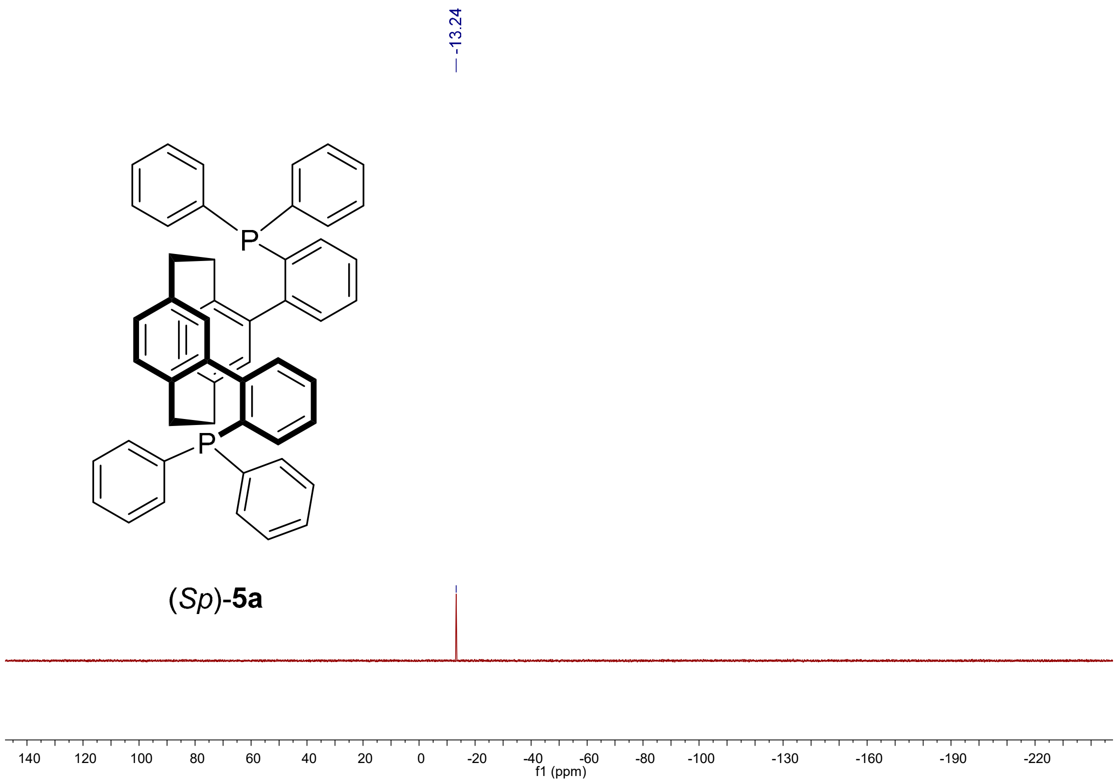

Figure S35. ^{31}P NMR (162 MHz, $CDCl_3$) spectrum of **(S_p)-5a**

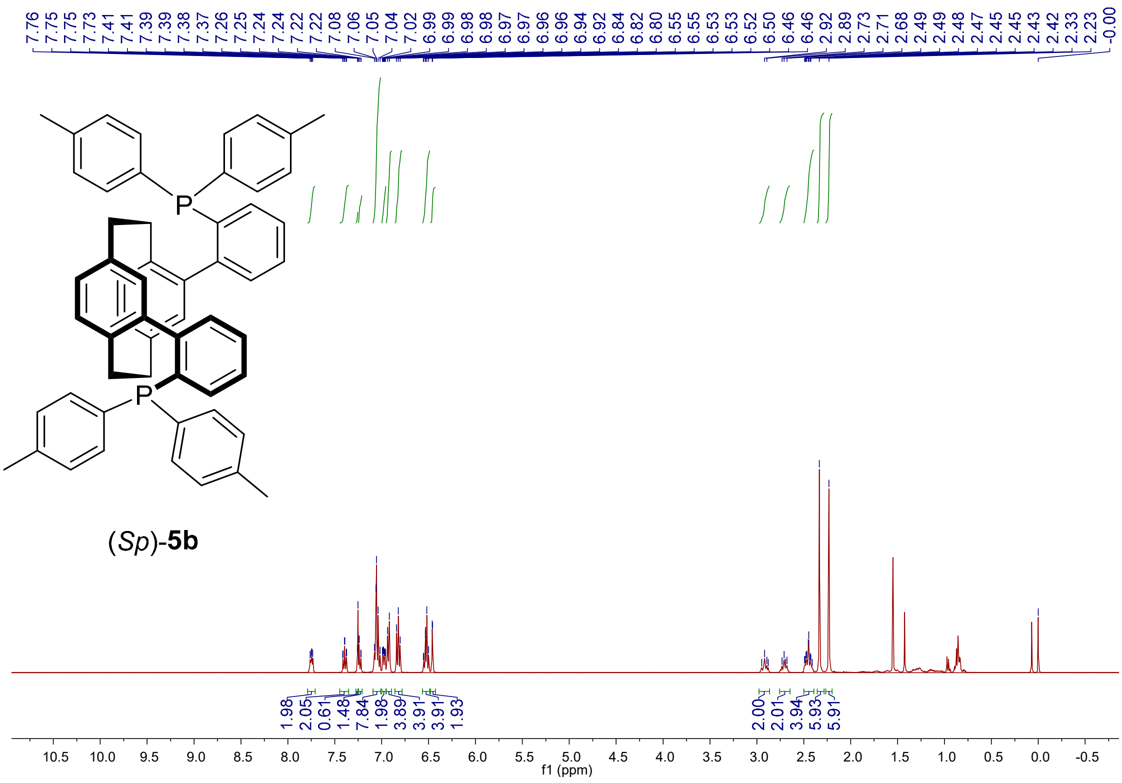

Figure S36. ^{1}H NMR (400 MHz, $CDCl_3$) spectrum of **(S_p)-5b**

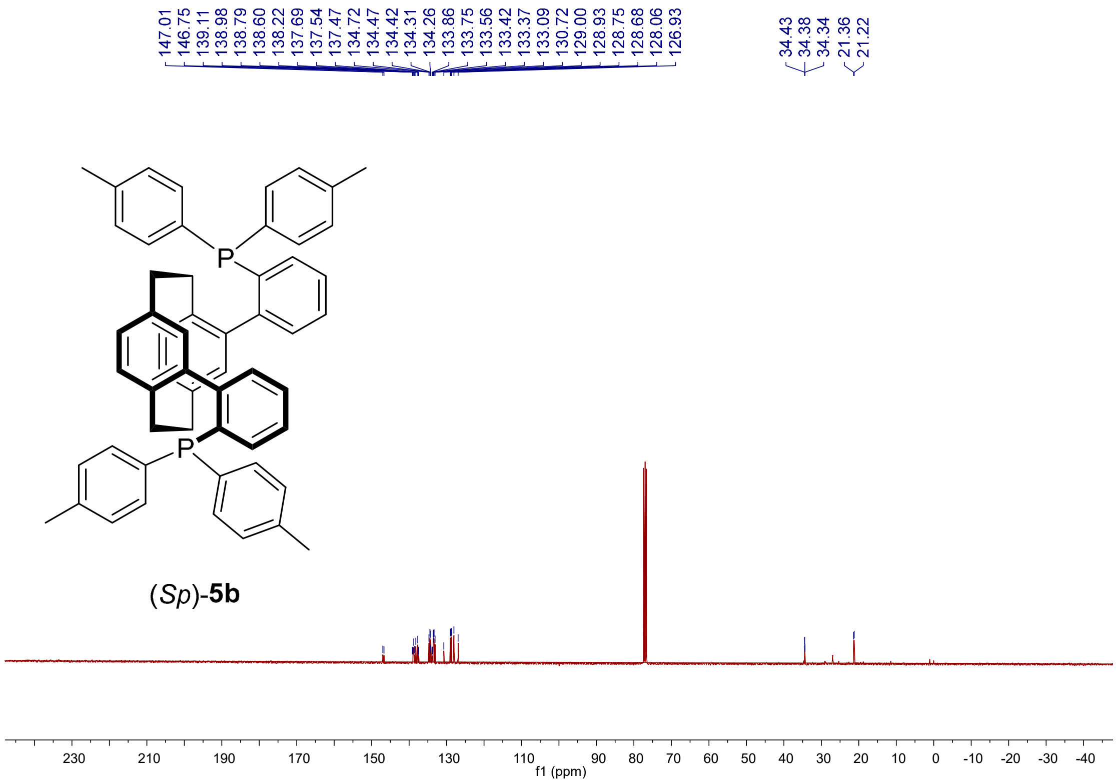

Figure S37. ^{13}C NMR (101 MHz, $CDCl_3$) spectrum of **(*S*$_p$)-5b**

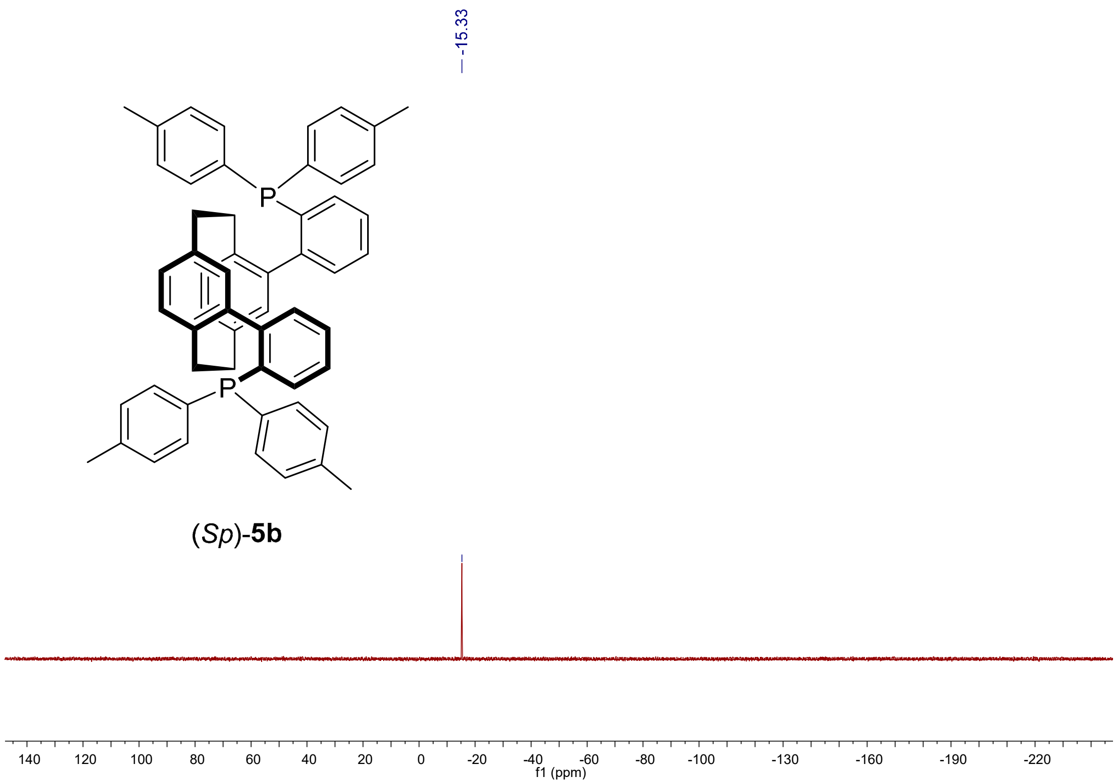

Figure S38. ^{31}P NMR (162 MHz, $CDCl_3$) spectrum of **(*S*$_p$)-5b**

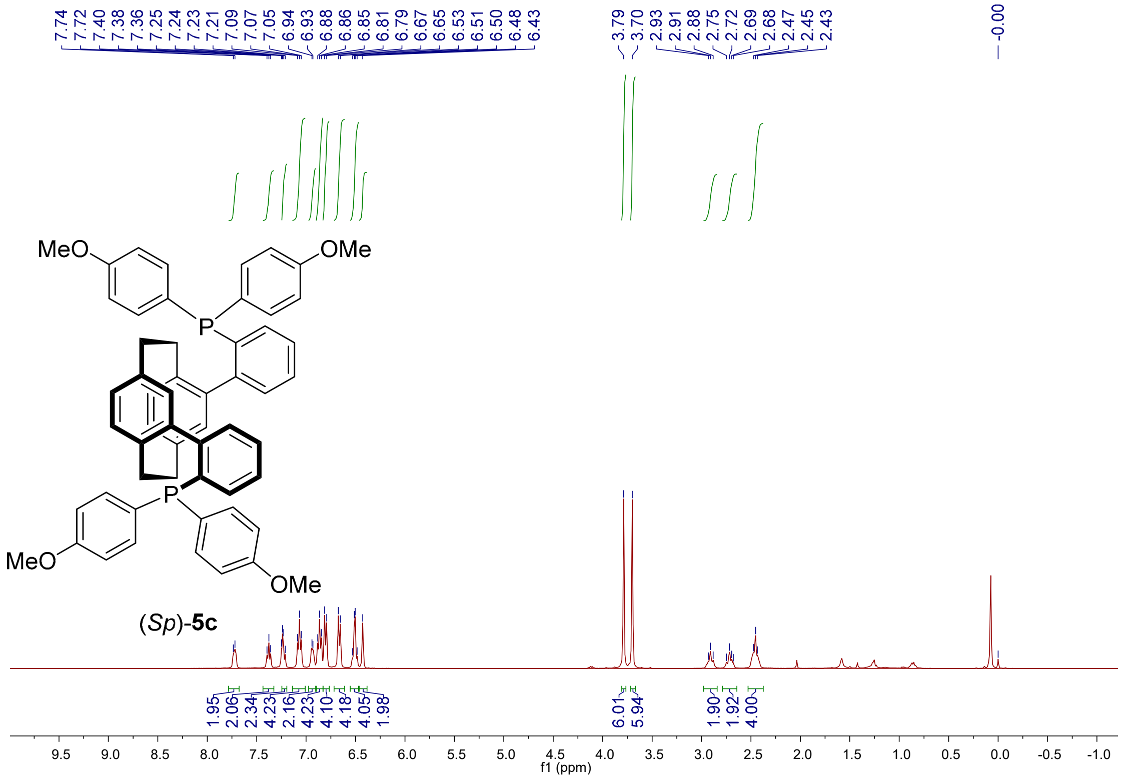

Figure S39. ^{1}H NMR (400 MHz, $CDCl_3$) spectrum of **(S_p)-5c**

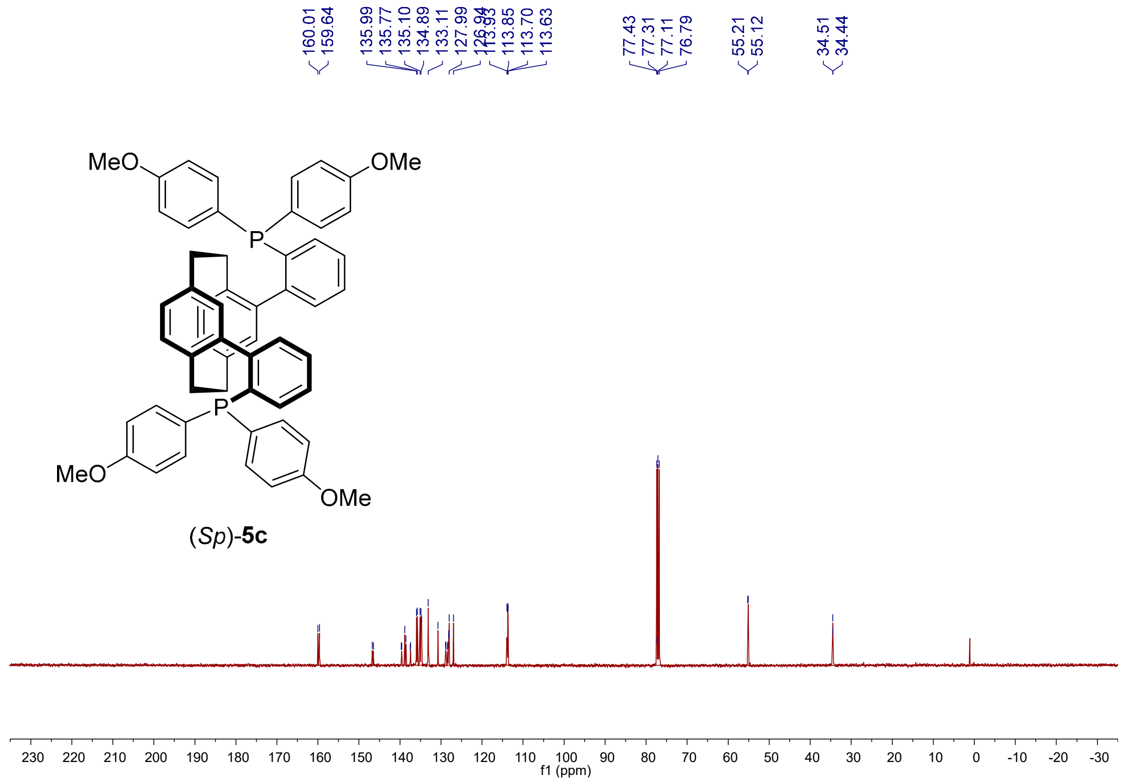

Figure S40. ^{13}C NMR (101 MHz, $CDCl_3$) spectrum of **(S_p)-5c**

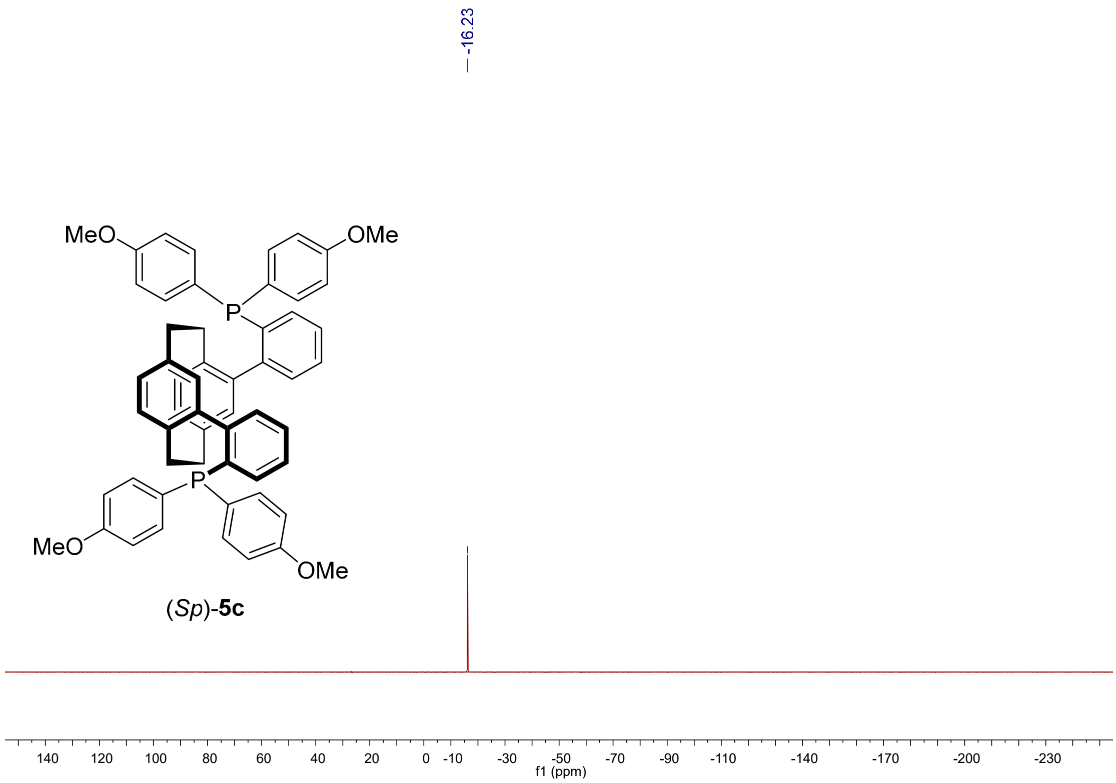

Figure S41. ^{31}P NMR (162 MHz, CDCl$_3$) spectrum of **(*S*$_p$)-5c**

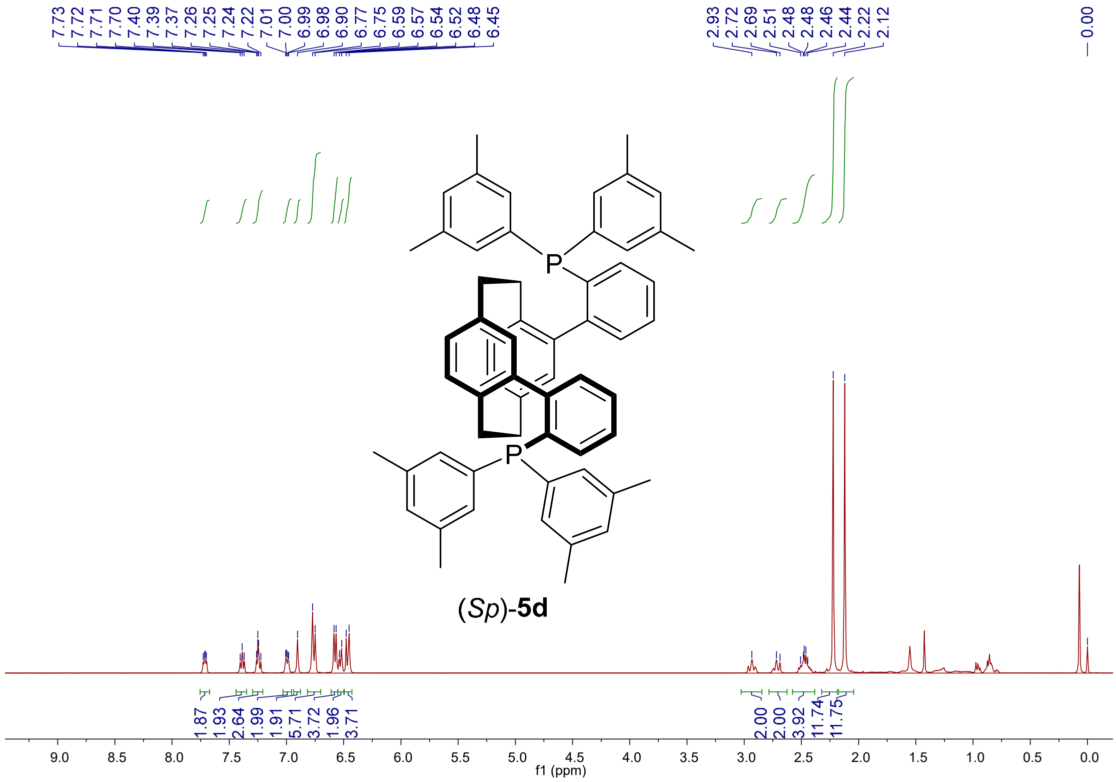

Figure S42. ^{1}H NMR (400 MHz, CDCl$_3$) spectrum of **(*S*$_p$)-5d**

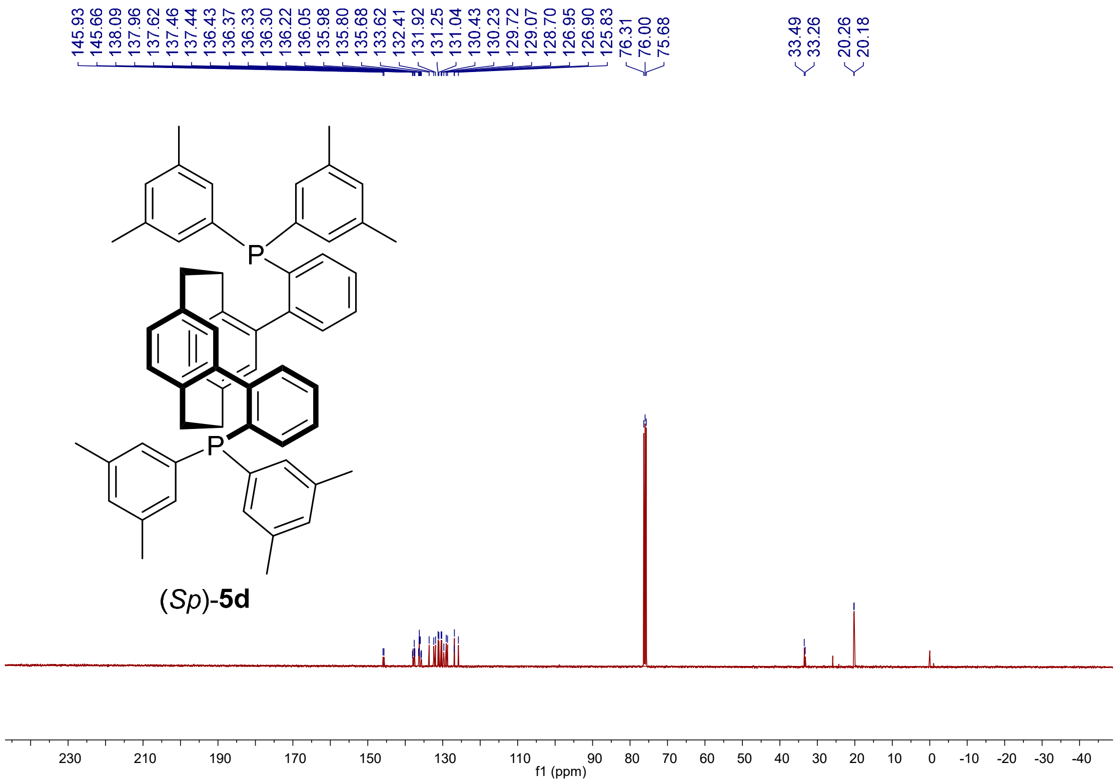

Figure S43. ^{13}C NMR (101 MHz, CDCl$_3$) spectrum of **(*S$_p$*)-5d**

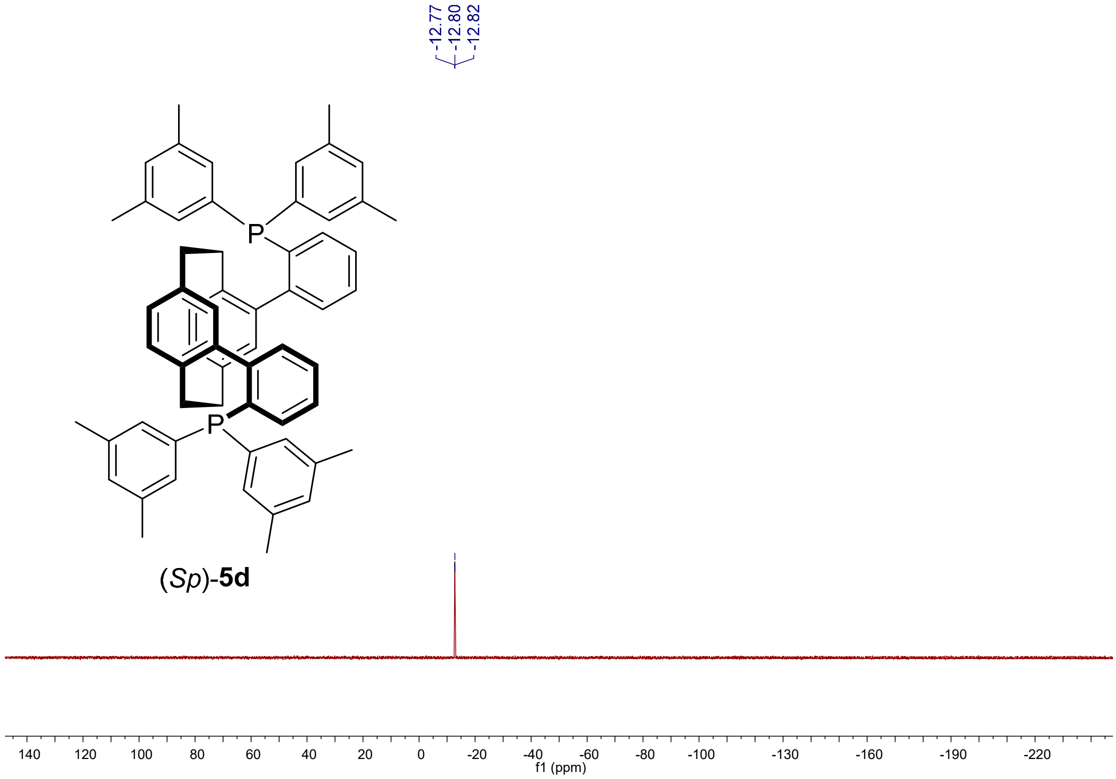

Figure S44. ^{31}P NMR (162 MHz, CDCl$_3$) spectrum of **(*S$_p$*)-5d**

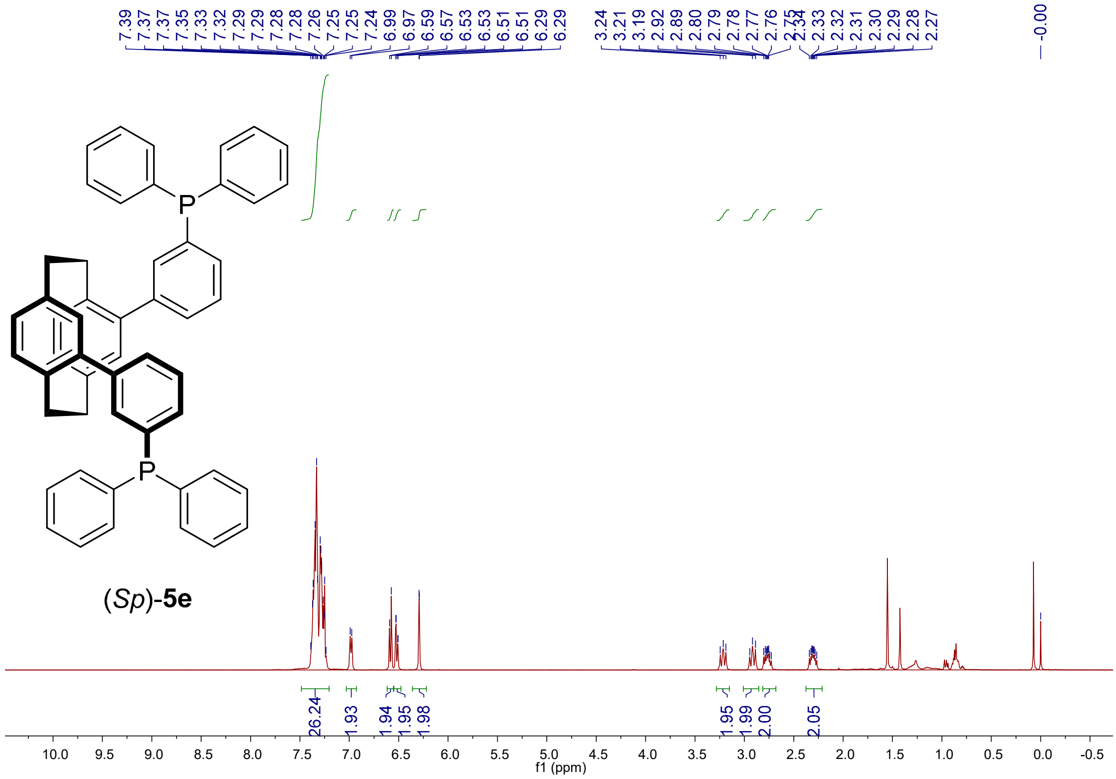

Figure S44. ^{1}H NMR (400 MHz, CDCl$_3$) spectrum of **(*S$_p$*)-5e**

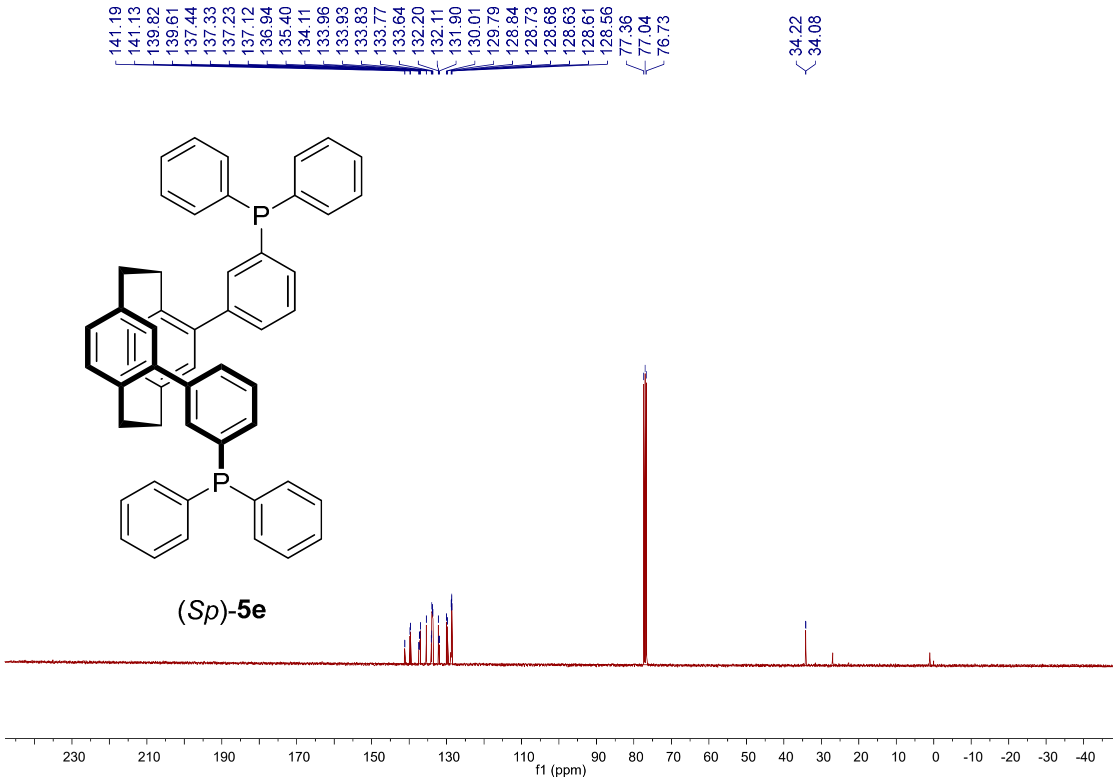

Figure S46. ^{13}C NMR (101 MHz, CDCl$_3$) spectrum of **(*S$_p$*)-5e**

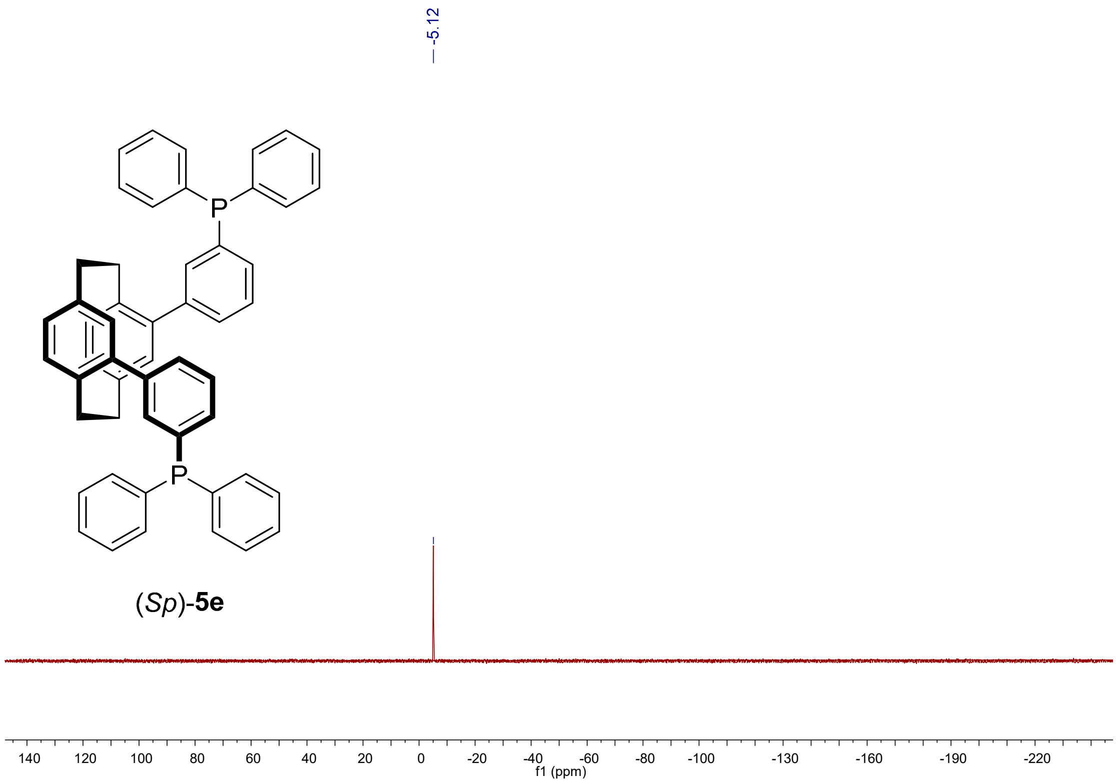

Figure S47. ^{31}P NMR (162 MHz, $CDCl_3$) spectrum of **(*S*$_p$)-5e**

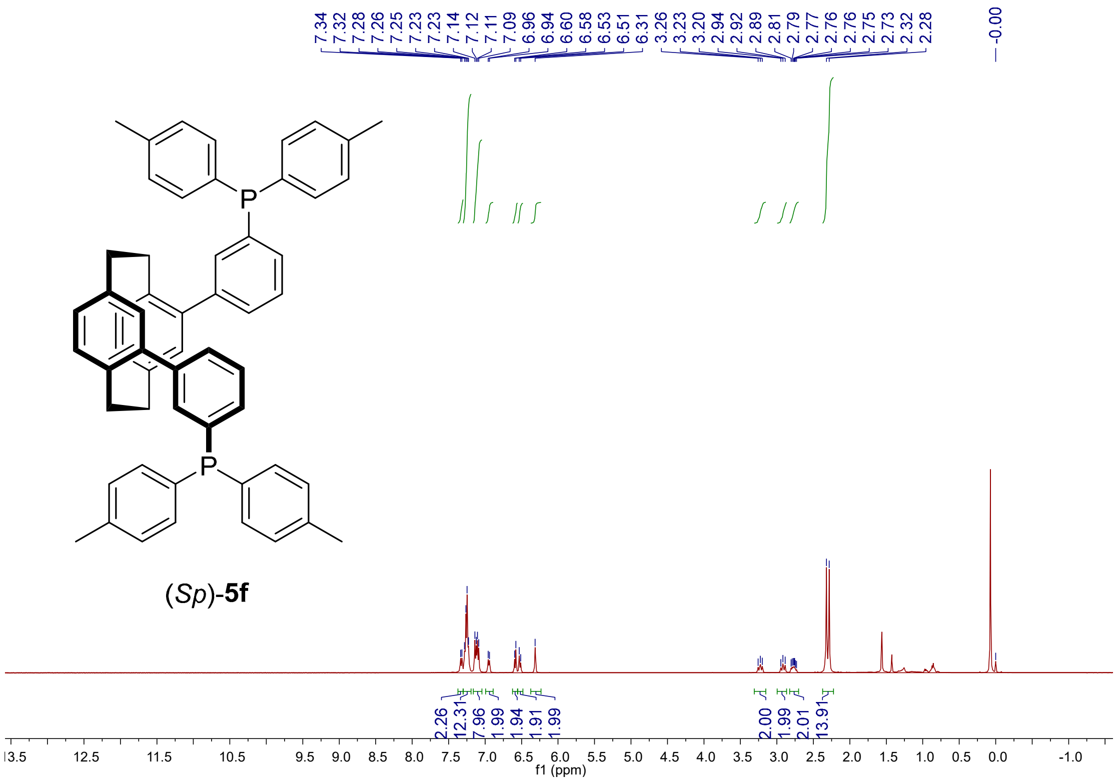

Figure S48. ^{1}H NMR (400 MHz, $CDCl_3$) spectrum of **(*S*$_p$)-5f**

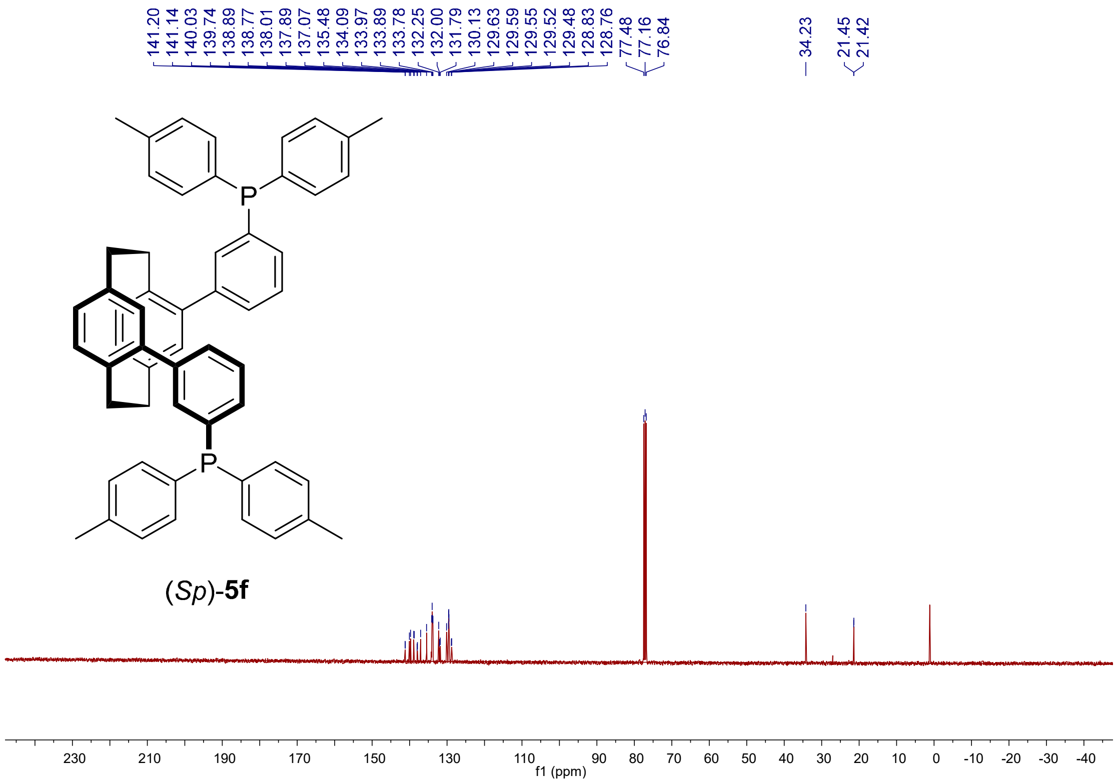

Figure S49. ^{13}C NMR (101 MHz, $CDCl_3$) spectrum of **(*S*$_p$)-5f**

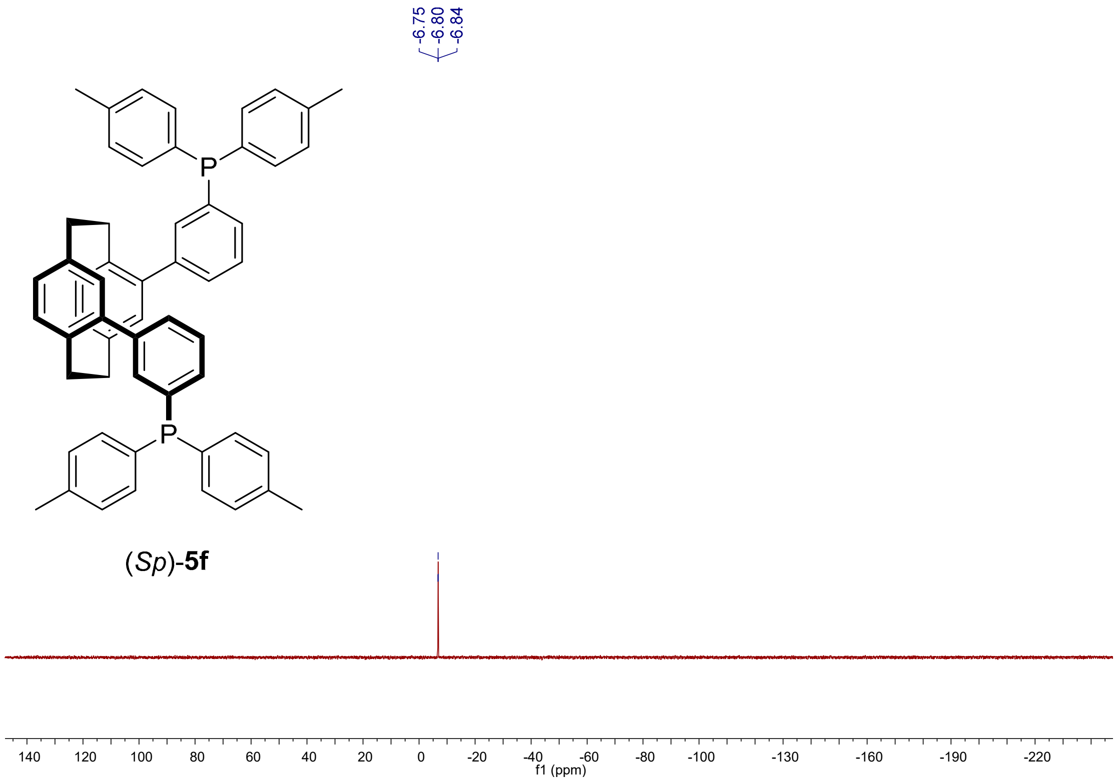

Figure S50. ^{31}P NMR (162 MHz, $CDCl_3$) spectrum of **(*S*$_p$)-5f**

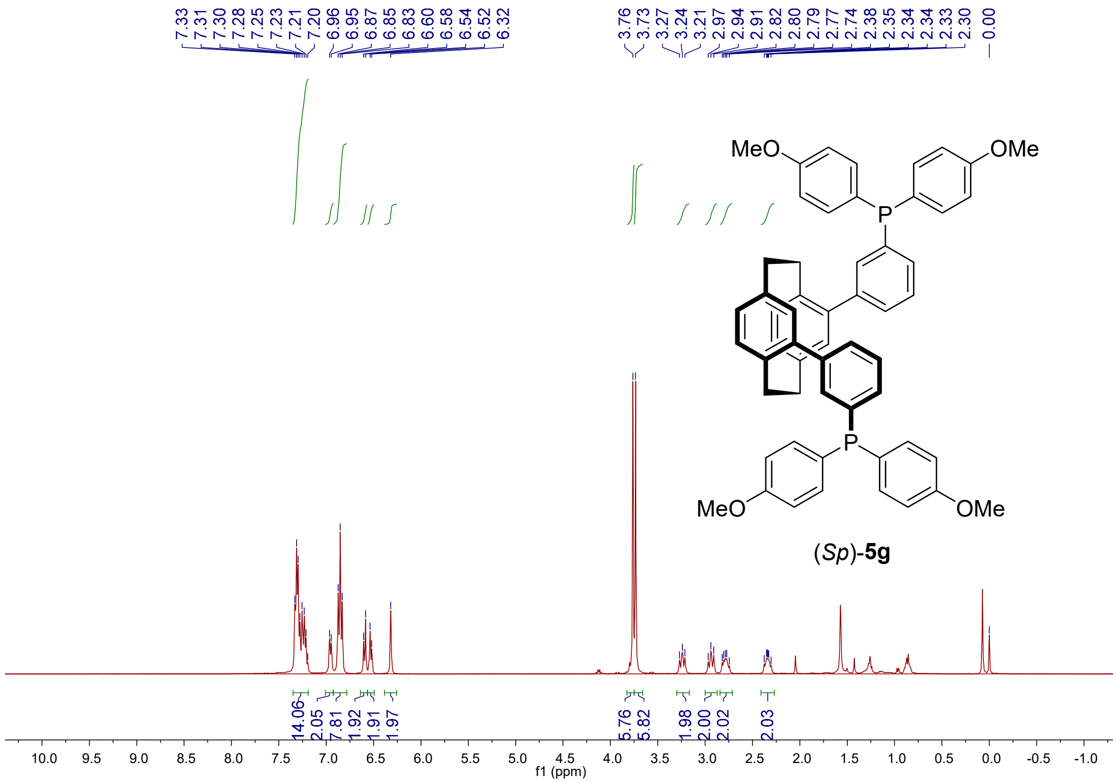

Figure S51. ^{1}H NMR (400 MHz, $CDCl_3$) spectrum of **(S_p)-5g**

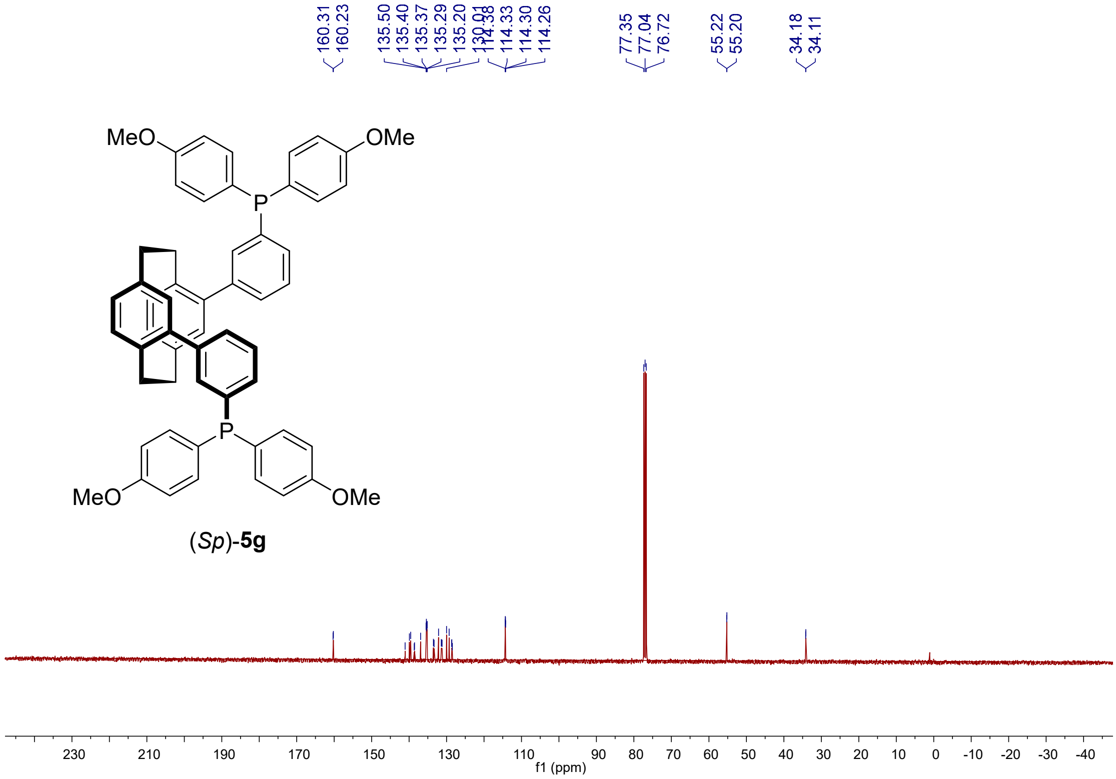

Figure S52. ^{13}C NMR (101 MHz, $CDCl_3$) spectrum of **(S_p)-5g**

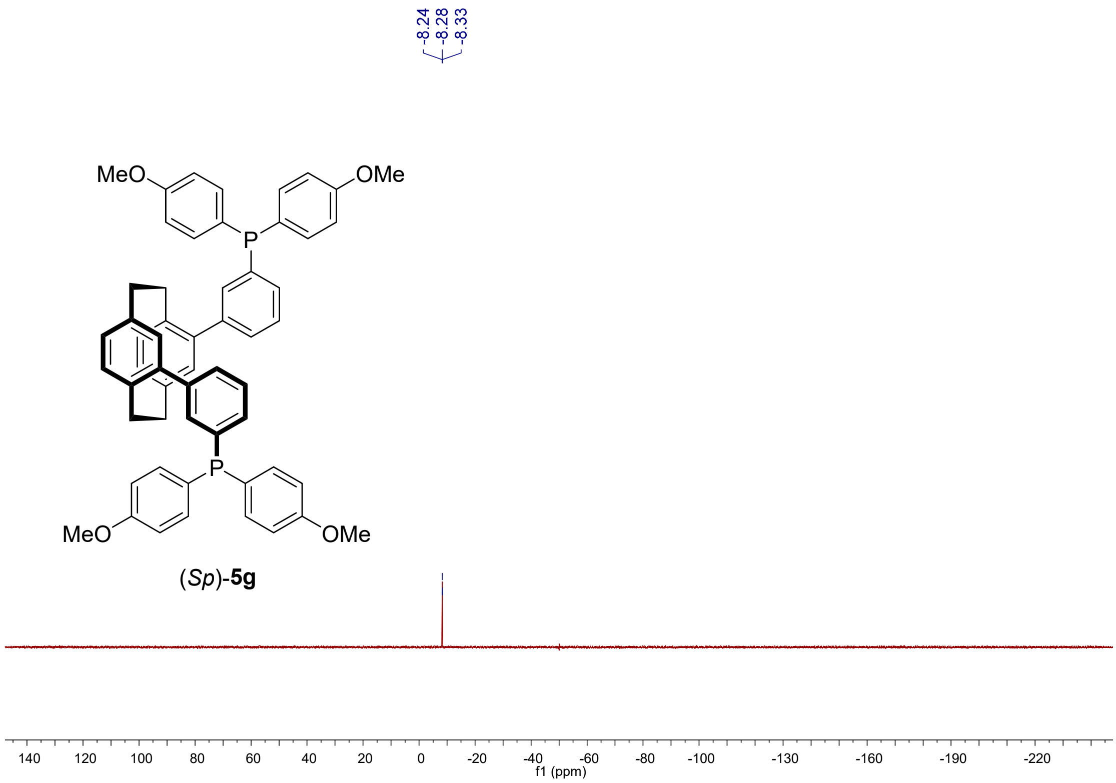

Figure S53. ^{31}P NMR (162 MHz, CDCl3) spectrum of **(*Sp*)-5g**

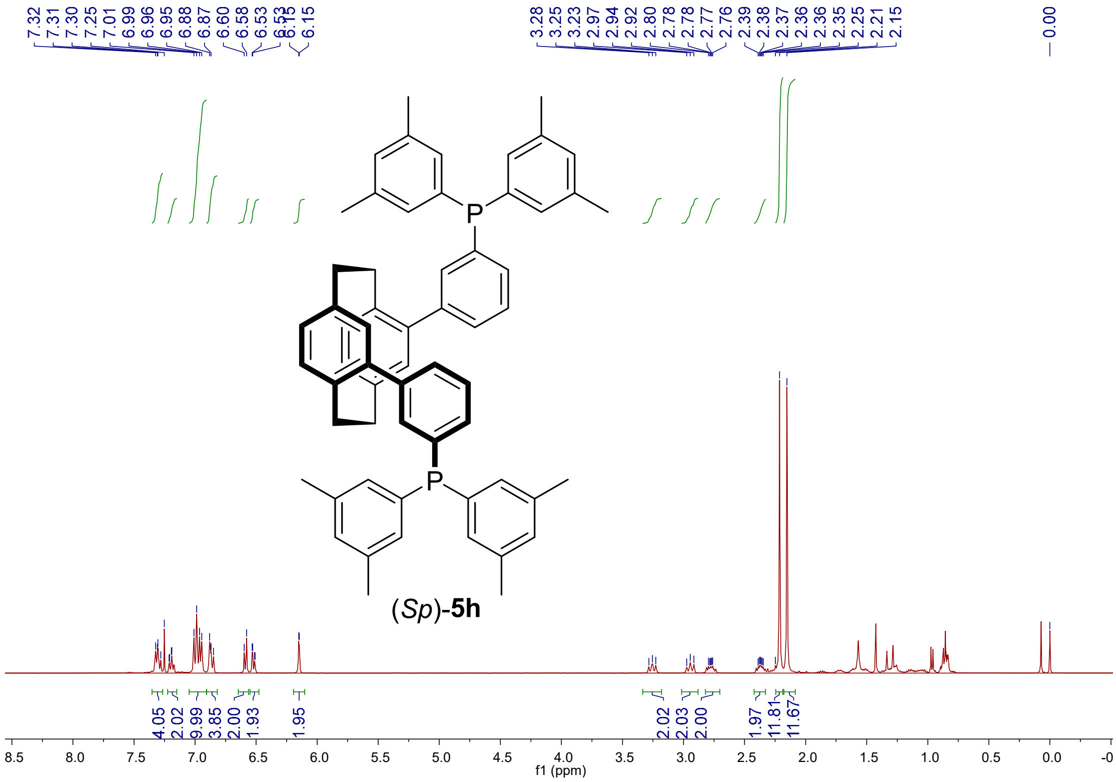

Figure S54. ^{1}H NMR (400 MHz, CDCl3) spectrum of **(*Sp*)-5h**

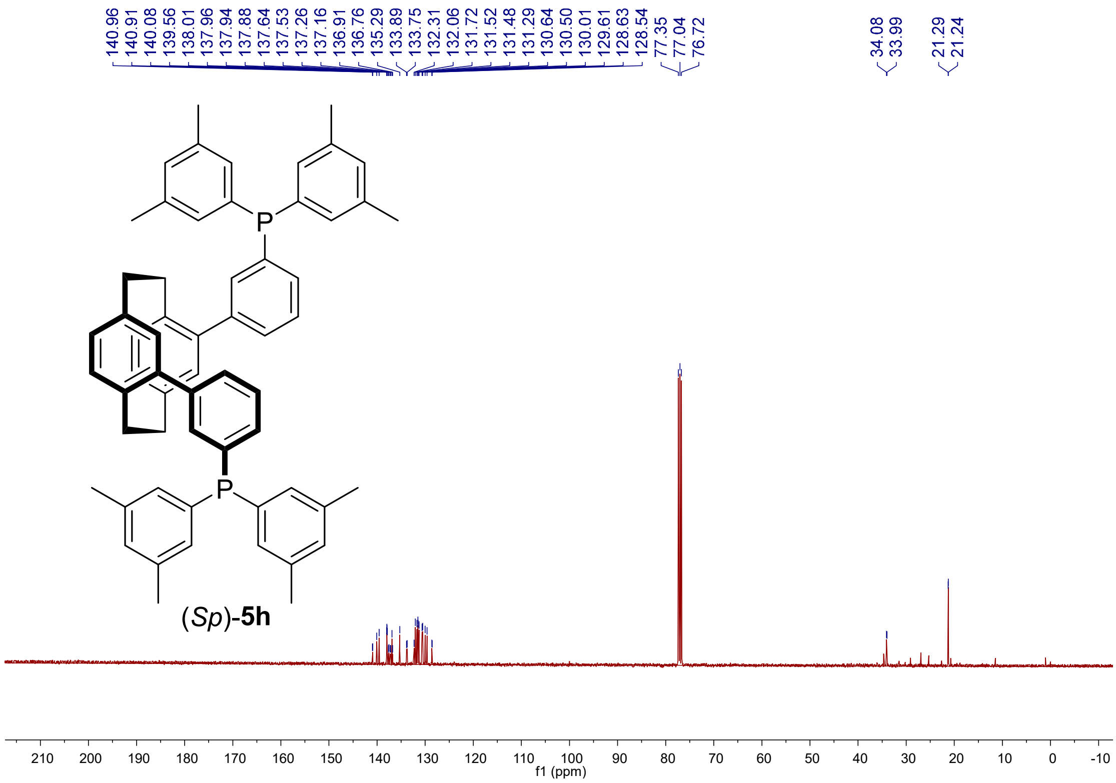

Figure S55. ^{13}C NMR (101 MHz, $CDCl_3$) spectrum of **(*S*$_p$)-5h**

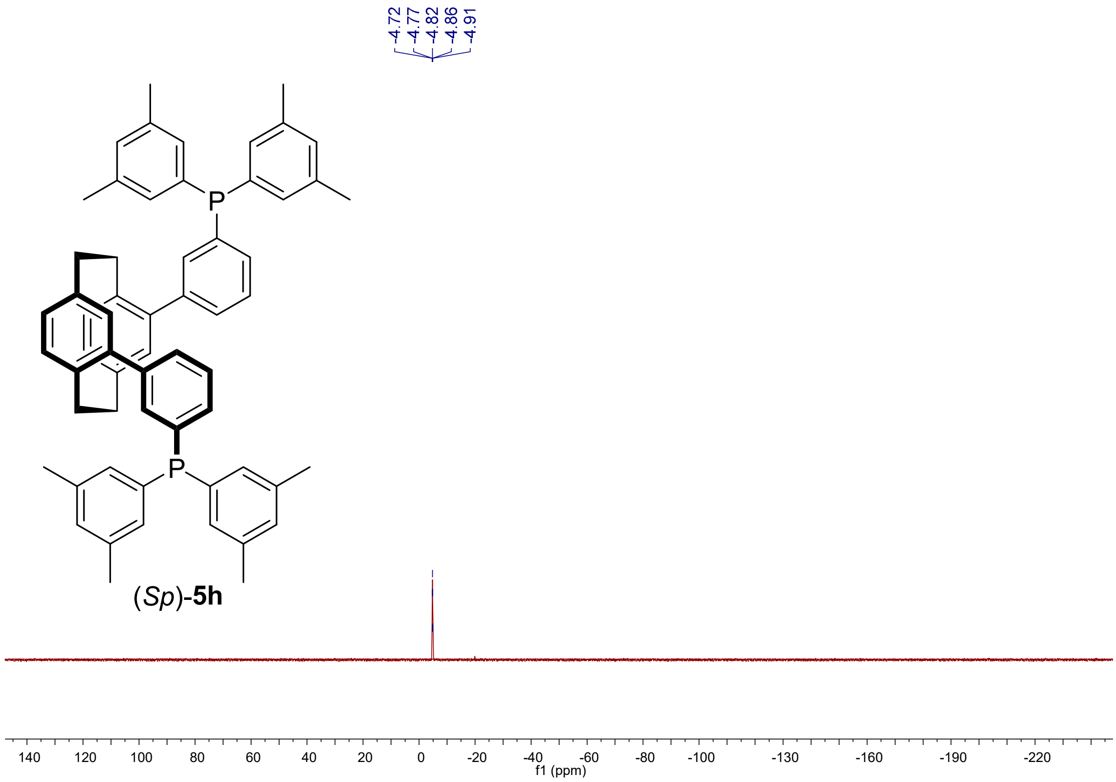

Figure S56. ^{31}P NMR (162 MHz, $CDCl_3$) spectrum of **(*S*$_p$)-5h**

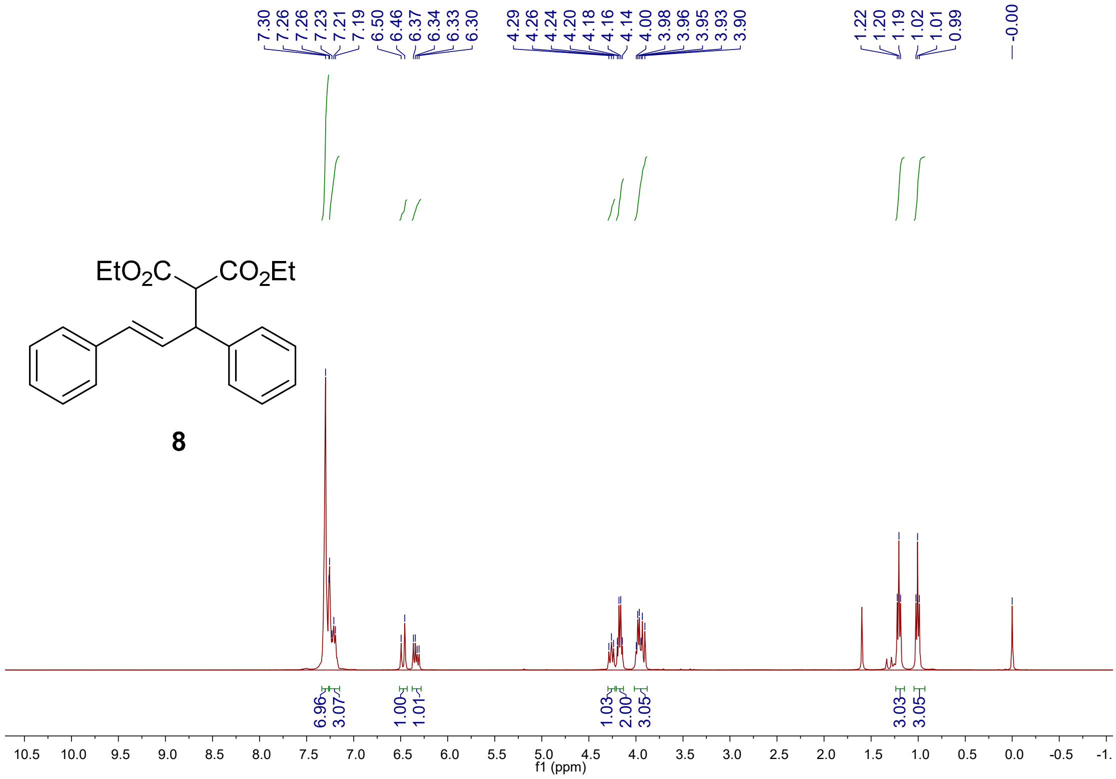

Figure S57. ^{1}H NMR (400 MHz, $CDCl_3$) spectrum of **8**

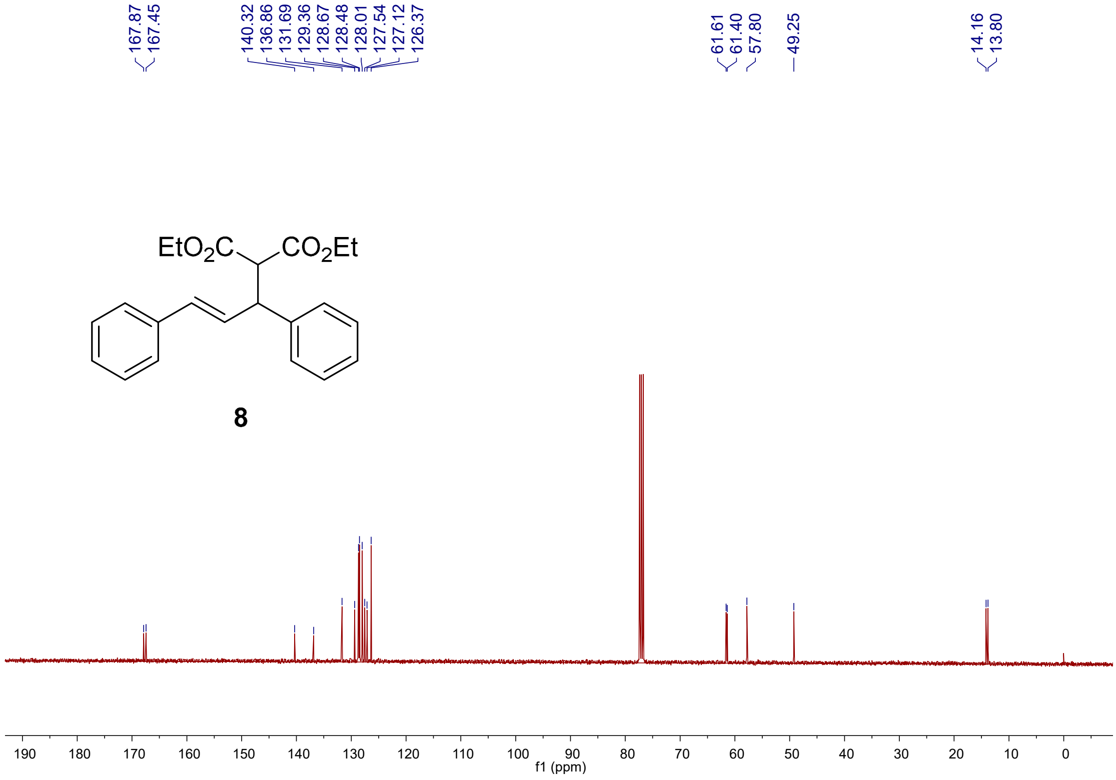

Figure S58. ^{1}H NMR (400 MHz, $CDCl_3$) spectrum of **8**

2. HPLC data

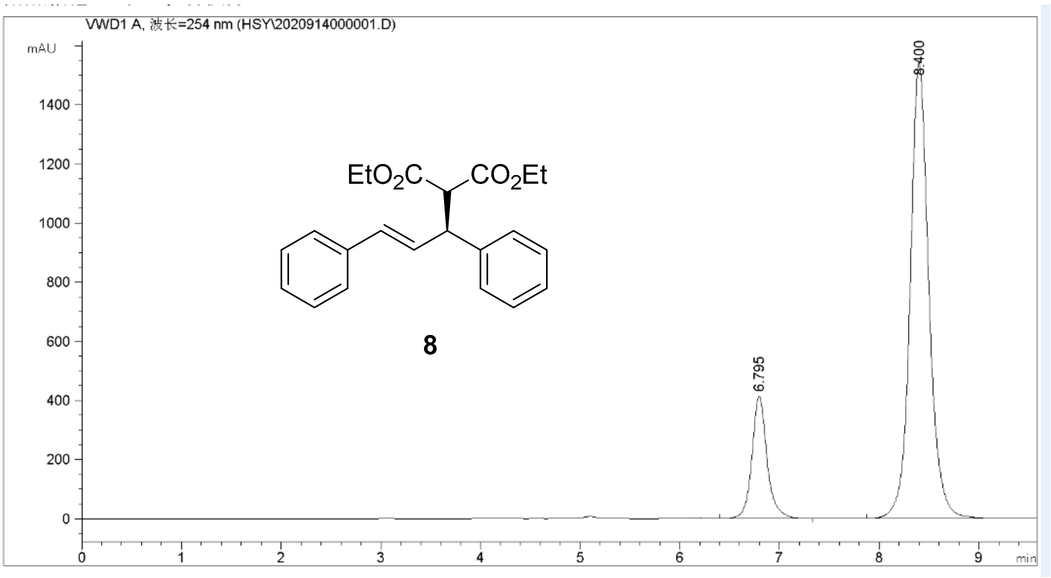

#	[min]		[min]	mAU *s	[mAU]	%
1	6.795	VB	0.1587	4398.60986	414.78192	17.8389
2	8.400	VBA	0.1985	2.02588e4	1541.42407	82.1611

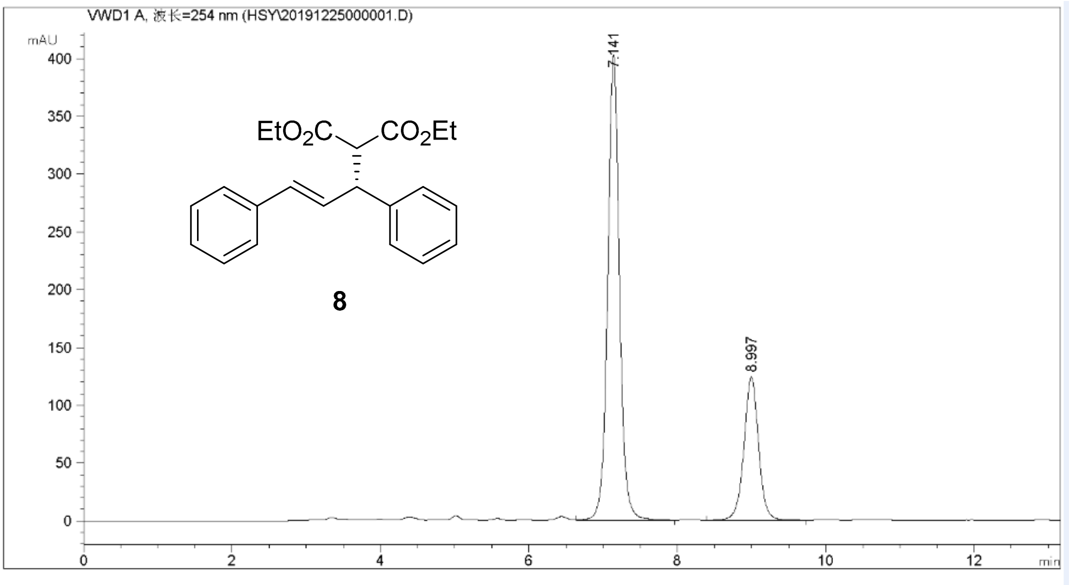

#	[min]		[min]	mAU *s	[mAU]	%
1	7.141	VB	0.1669	4451.15332	402.55063	72.3869
2	8.997	BB	0.2078	1697.96265	124.02995	27.6131

3. references

1 Chang, S.; Wang, L.; Lin, X. Synthesis and Application of a New Hexamethyl-1,1′-Spirobiindane-Based Chiral Bisphosphine (HMSI-PHOS) Ligand in Asymmetric Allylic Alkylation. *Org. Biomol. Chem.* **2018**, *16*, 2239–2247.
